# Nociceptor neurons affect cancer immunosurveillance

**DOI:** 10.1038/s41586-022-05374-w

**Published:** 2022-11-02

**Authors:** Mohammad Balood, Maryam Ahmadi, Tuany Eichwald, Ali Ahmadi, Abdelilah Majdoubi, Karine Roversi, Katiane Roversi, Christopher T. Lucido, Anthony C. Restaino, Siyi Huang, Lexiang Ji, Kai-Chih Huang, Elise Semerena, Sini C. Thomas, Alexandro E. Trevino, Hannah Merrison, Alexandre Parrin, Benjamin Doyle, Daniel W. Vermeer, William C. Spanos, Caitlin S. Williamson, Corey R. Seehus, Simmie L. Foster, Hongyue Dai, Chengyi J. Shu, Manu Rangachari, Jacques Thibodeau, Sonia V. Del Rincon, Ronny Drapkin, Moutih Rafei, Nader Ghasemlou, Paola D. Vermeer, Clifford J. Woolf, Sebastien Talbot

**Affiliations:** 1 Département de Pharmacologie et Physiologie, Université de Montréal, Montréal, Quebec Canada; 2 Département de Médecine Moléculaire, Faculté de Médecine, Université Laval, Québec, Quebec Canada; 3 Departamento de Bioquímica, Universidade Federal de Santa Catarina, Florianópolis, Brazil; 4 Département de Microbiologie, Infectiologie et Immunologie, Université de Montréal, Montréal, Quebec Canada; 5 Cancer Biology and Immunotherapies, Sanford Research, Sioux Falls, SD USA; 6 Cygnal Therapeutics, Cambridge, MA USA; 7 F.M. Kirby Neurobiology Center, Boston Children’s Hospital, Boston, MA USA; 8 Department of Neurobiology, Harvard Medical School, Boston, MA USA; 9 Depression Clinical Research Program, Massachusetts General Hospital, Boston, MA USA; 10 Department of Oncology, McGill University, Montréal, Quebec Canada; 11 Penn Ovarian Cancer Research Center, Perelman School of Medicine, University of Pennsylvania, Philadelphia, PA USA; 12 Department of Biomedical and Molecular Sciences, Queen’s University, Kingston, Ontario Canada

**Keywords:** Immunosurveillance, Neuroimmunology

## Abstract

Solid tumours are innervated by nerve fibres that arise from the autonomic and sensory peripheral nervous systems^1–5^. Whether the neo-innervation of tumours by pain-initiating sensory neurons affects cancer immunosurveillance remains unclear. Here we show that melanoma cells interact with nociceptor neurons, leading to increases in their neurite outgrowth, responsiveness to noxious ligands and neuropeptide release. Calcitonin gene-related peptide (CGRP)—one such nociceptor-produced neuropeptide—directly increases the exhaustion of cytotoxic CD8^+^ T cells, which limits their capacity to eliminate melanoma. Genetic ablation of the TRPV1 lineage, local pharmacological silencing of nociceptors and antagonism of the CGRP receptor RAMP1 all reduced the exhaustion of tumour-infiltrating leukocytes and decreased the growth of tumours, nearly tripling the survival rate of mice that were inoculated with B16F10 melanoma cells. Conversely, CD8^+^ T cell exhaustion was rescued in sensory-neuron-depleted mice that were treated with local recombinant CGRP. As compared with wild-type CD8^+^ T cells, *Ramp1*^−/^^−^ CD8^+^ T cells were protected against exhaustion when co-transplanted into tumour-bearing *Rag1*-deficient mice. Single-cell RNA sequencing of biopsies from patients with melanoma revealed that intratumoral *RAMP1*-expressing CD8^+^ T cells were more exhausted than their *RAMP1*-negative counterparts, whereas overexpression of *RAMP1* correlated with a poorer clinical prognosis. Overall, our results suggest that reducing the release of CGRP from tumour-innervating nociceptors could be a strategy to improve anti-tumour immunity by eliminating the immunomodulatory effects of CGRP on cytotoxic CD8^+^ T cells.

## Abstract (web summary)

Melanoma cells interact with pain-mediating sensory neurons by increasing their release of the neuropeptide CGRP, which increases the exhaustion of CD8^+^ T cells and thus promotes the survival of cancer cells.

## Main

Cytotoxic T cells express a variety of receptors, including PD-1 (programmed cell death protein 1), LAG3 (lymphocyte activation gene-3 protein) and TIM3 (T cell immunoglobulin and mucin domain-containing protein 3)^6–8^, which inhibit the function of T cells after being activated by their cognate ligands. These checkpoint receptors ensure that immune responses to damage or infection are kept in check, thus preventing overly intense responses that might damage healthy cells^9^. Tumour cells express ligands for these immune checkpoints, which, when activated, block the cytolytic functions of T cells, thereby favouring the survival of cancer cells^9,10^.

In prostate cancer, doublecortin-expressing neural progenitors initiate autonomic adrenergic neurogenesis^3^, which facilitates the development and dissemination of tumours^2^. In head and neck tumours, a loss of TP53 drives the reprogramming of tumour-innervating sensory nerves into adrenergic neurons that promote tumour growth^1^. The presence of such neo-innervation in cancer, together with the diverse actions of neuropeptides on immune cells^11–18^, led us to examine whether the local release of neuropeptides from activated nociceptors could favour cancer growth by suppressing immune surveillance.

## Melanomas are innervated

**Extended Data Fig. 1:**
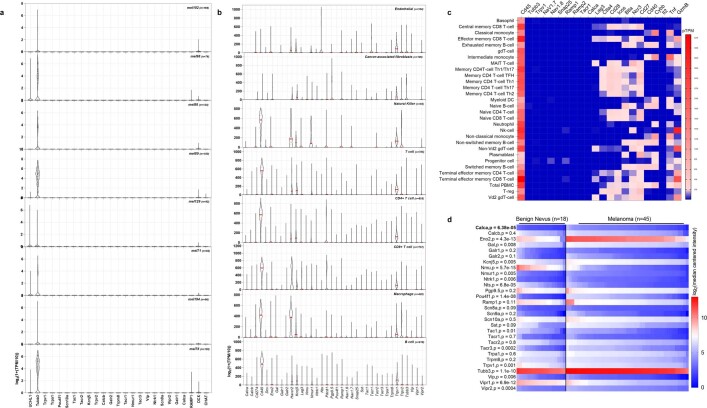
*TRPV1*, *NAV1.8*, *SNAP25* or *RAMP1* transcripts are expressed in patient melanoma biopsies but are not detected in human immune cells or malignant cells. (**a–b**) In silico analysis of single-cell RNA sequencing of human melanoma-infiltrating cells revealed that *Trpv1*, *Nav1.8* (Scn10a), *Snap25* (the molecular target of BoNT/A), *Calca* (gene encoding for CGRP) transcripts are not detected in malignant melanoma cells (defined as CD90^–^CD45^–^) from ten different patients’ biopsies (**a**) nor in cancer-associated fibroblasts, macrophage, endothelial, natural killer, T, and B cells (**b**). Individual cell data are shown as a log_2_ of 1 + (transcript per million / 10). Experimental details and cell clustering were defined in Jerby-Arnon et al^41^. N are defined in the figures. (**c**) *In silico* analysis of human immune cells revealed their basal expression of *Cd45*. Using RNA sequencing approaches, *Calca*, *Snap25*, *Trpv1* or *NaV1.8* are not detected in these cells. Heat maps show the read counts normalized to transcripts per million protein-coding genes (pTPM) for each of the single-cell clusters. Experimental details and cell clustering were defined in Monaco et al^73^. (**d**) Forty-five cutaneous melanomas and 18 benign melanocytic skin nevus biopsies transcriptomes were profiled using Affymetrix U133A microarrays^19^. In silico analysis of this dataset revealed that cutaneous melanoma heightened expression levels of *Calca (1.4-fold)*, *Pouf4f1 (2-fold)*, *Eno2 (1.4-fold)*, and *Tubb3 (1.1-fold)*, as well as other neuronal-enriched genes. Heat map data are shown as log_2_ (median centred intensity); two-sided unpaired Student’s t-test; p-values and n are shown in the figure. Experimental details were defined in Haqq et al^19^. Source Data

**Extended Data Fig. 2:**
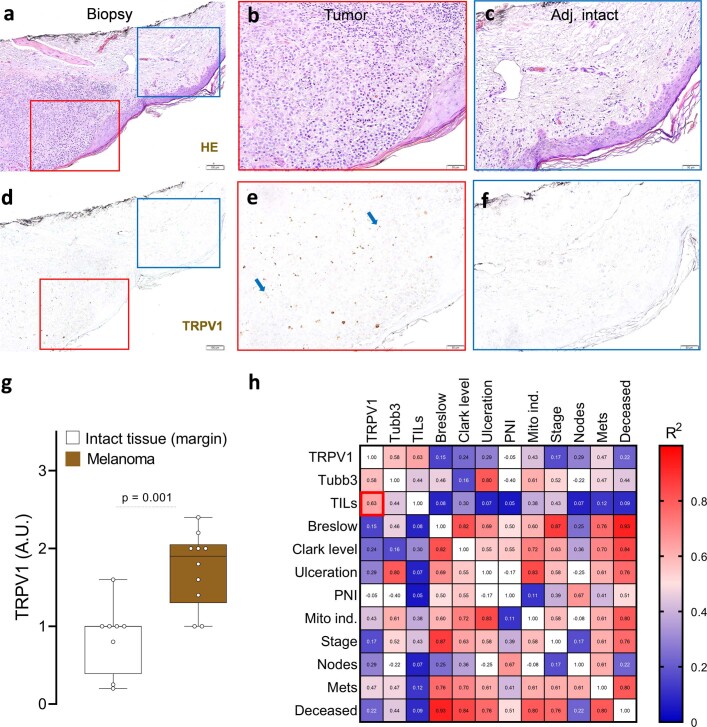
TRPV1^+^ neurons innervate patient melanomas. Patients’ melanoma sections were stained with hematoxylin eosin (**a–f**), and the presence of TRPV1 (**d–f**; brown) neurons was analysed by immunohistochemistry. Increased levels of TRPV1^+^ neurons (**g**) were found in the tumour (delimited by red square; **a-b**, **d-e**) compared to adjacent healthy skin (delimited by blue square; **a**,**c**,**d**,**f**). Increased TRPV1 immunolabelling in tumour sections primarily correlated with enhanced levels of tumour-infiltrating leukocytes (**h**) as scored from a retrospective correlation analysis performed on the patients’ pathology reports. Data are shown as representative immunohistochemistry images (**a–f**), box-and-whisker plots (runs from minimal to maximal values; the box extends from 25^th^ to 75^th^ percentile and the middle line indicates the median), for which individual data points are given (**g**) or as a heat map (**h**) displaying Pearson’s correlation (R^2^). N are as follows: **a–f**: n=10, **g**: intact (n = 8), tumour (n = 10), **h**: n = 10. Slides were scored blindly by two experienced medical pathologists. P-values are shown in the figure and determined by two-sided unpaired Student’s t-test (**g**). Scale = 100 μm (**a**,**d**), 50 μm (**b**,**c**,**e**,**f**). Source Data

Although the expression of genes of neuronal origin is not detected by RNA-sequencing approaches in human malignant cells or immune cells (Extended Data Fig. 1a–c), we observed a significant increase in their expression in biopsies from patients with melanoma^19–22^ (Extended Data Fig. 1d). As these clinical data suggested increased innervation of melanomas, we tested for the presence of nociceptor neurons by assessing TRPV1^+^ neurons in biopsies from patients with melanoma. TRPV1 immunolabelling was increased by around twofold in the tumour compared to adjacent healthy tissue in each of the ten biopsies examined. The numbers of tumour-infiltrating lymphocytes (TILs) correlated (*R*^2^ = 0.63) with increased TRPV1 immunolabelling (Extended Data Fig. 2). These data indicate that melanomas are innervated by sensory neurons and that these neurons may affect the intratumoral numbers of immune cells.

**Fig. 1:**
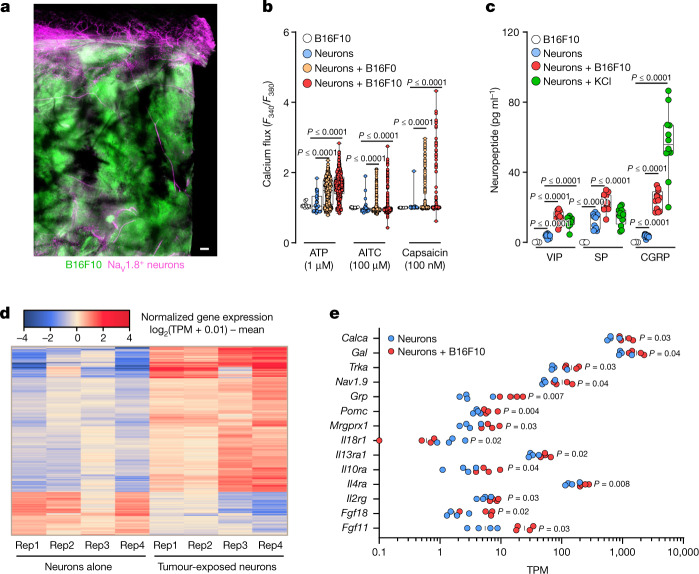
Melanoma cells sensitize nociceptors. **a**, Nociceptor (*Nav1.8*^*cre*^*::tdTomato*^*fl/WT*^; magenta) reporter mice were inoculated in the hindpaw with B16F10-eGFP cancer cells (i.d., 2 × 10^5^ cells; green). Representative image of Na_V_1.8^+^ nerve fibres (magenta) innervating B16F10-eGFP-inoculated mouse skin after 22 days. Scale bar, 200 μm. **b**, In co-culture, B16F0 or B16F10 cells sensitize the response of nociceptors to capsaicin (100 nM), allyl isothiocyanate (AITC, 100 μM) and ATP (1 μM), as measured by calcium flux. A low concentration of the ligands induces a minimal response in control neurons, whereas B16F10 cells show marginal sensitivity to ATP. **c**, Dorsal root ganglion (DRG) neurons co-cultured (96 h) with B16F10 cells release substance P (SP), vasoactive intestinal peptide (VIP) and CGRP. B16F10 cells alone do not release neuropeptides. Stimulation with KCl (40 mM; 30 min) induced a significant release of neuropeptides from cultured neurons. **d**,**e**, Naive DRG neurons (*Trpv1*^*cre*^*::-CheRiff-eGFP*^*fl/WT*^) were cultured alone or in combination with B16F10-mCherry-OVA cells. After 48 h, the cells were collected, FACS purified and RNA sequenced. Hierarchical clustering of DEGs from the sorted neurons shows distinct groups of transcripts enriched in cancer-exposed TRPV1^+^ neurons (**d**), including *Calca* (the gene encoding CGRP; **e**). Data are shown as a representative image (**a**), as box-and-whisker plots (running from minimal to maximal values; the box extends from 25th to 75th percentile and the middle line indicates the median), for which individual data points are given (**b**,**c**), as a heat map showing normalized gene expression (log_2_(0.01 + transcripts per million reads (TPM)) − mean (**d**) or as a scatter dot plot with medians (**e**). Experiments were independently repeated two (**a**) or three (**b**,**c**) times with similar results. The sequencing experiment was not repeated (**d**,**e**). *n* as follows: **a**: *n* = 4; **b**: neurons (29 neurons from 10 mice), B16F10 (16 cells from 10 dishes), neurons + B16F0 (387 neurons from 12 mice), neurons + B16F10 (409 neurons from 12 mice); **c**: neurons (*n* = 12), neurons + B16F10 (*n* = 12), neurons + KCl (*n* = 12), B16F10 (*n* = 3); **d**,**e**: *n* = 4 per group. *P* values were determined by one-way ANOVA with post-hoc Bonferroni (**b**,**c**) or two-sided unpaired Student’s *t*-test (**e**). Source Data

**Extended Data Fig. 3:**
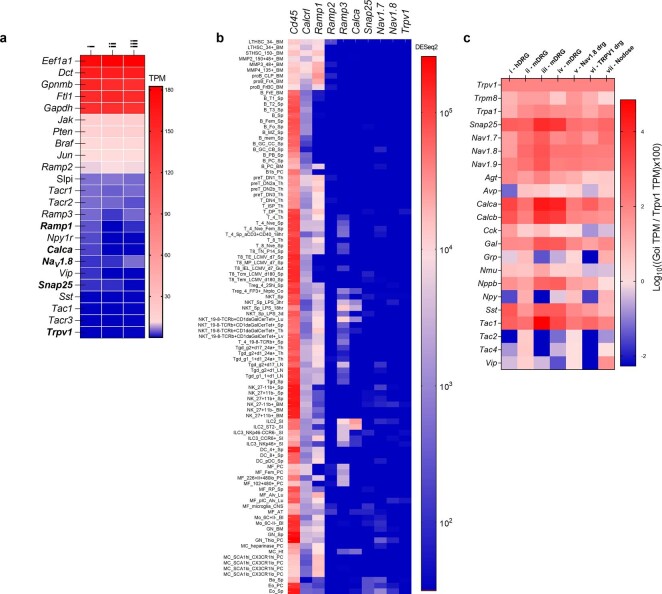
*Trpv1*, *Nav1.8*, *Snap25* or *Ramp1 transcripts* are not detected in B16F10 cancer cells or mouse immune cells. (**a**) *In silico* analysis of three different B16F10 cells cultures (labelled as i, ii, iii)^74^ revealed their basal expression of *Braf* and *Pten*. In contrast, *Calca*, *Snap25*, *Trpv1* or *NaV1.8* transcripts are not detected in B16F10 cells. Heat map data are shown as transcript per million (TPM) on a linear scale. Experimental details were defined in Castle et al^74^. N=3/group. (**b**) ImmGen RNA sequencing of leukocyte subpopulations^76^ reveals their basal expression of *Cd45* and *Ramp1*. In contrast, *Snap25*, *Trpv1*, or *Nav1.8* transcripts are not detected in mouse immune cells. Heat map data are shown as DESeq_2_ on a logarithmic scale. (**c**) A meta-analysis of seven published nociceptor neuron expression profiling datasets^66^ revealed the basal expression of sensory neuron markers (*Trpv1*, *Trpa1*) and neuropeptides (*Sp*, *Vip*, *Nmu*, *Calca*). Expression across datasets was ratioed over Trpv1 and multiplied by 100. The log_10_ of these values is presented as a heat map. i) RNA sequencing of human lumbar neurons;^72^ ii) microarrays of mouse FACS-sorted Na_V_1.8^+^ neurons;^70^ iii) and iv) single-cell RNA sequencing of mouse lumbar neurons;^68,69^ v) microarray profiling of mouse Na_V_1.8^+^ DRG neurons;^70^ vi) performed RNA sequencing of mouse TRPV1^+^ neurons;^71^ and vii) single-cell RNA sequencing of mouse vagal ganglia^67^. Source Data

**Extended Data Fig. 4:**
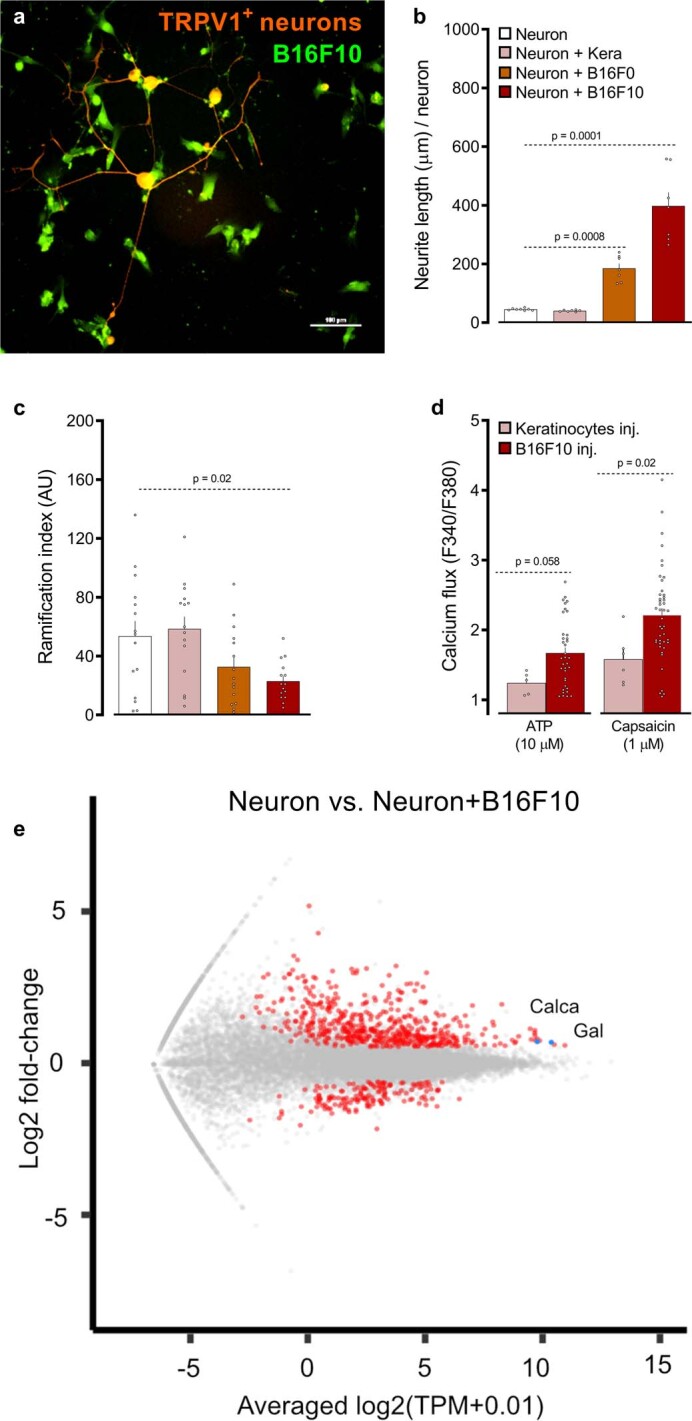
B16F10 cells interact with nociceptor neurons. (**a–c**) When co-cultured with B16F10-eGFP cells (green), TRPV1^+^ nociceptor (*Trpv1*^*cre*^*::tdTomato*^*fl/WT*^; orange) neurons form neuro-neoplasic contacts (**a**), show longer neurites (**b**), and exhibit reduced arborization (**c**) than when cultured alone or with non-tumorigenic keratinocytes (**b–c**). (**d**) L3–L5 DRG neurons were collected from mice 2-weeks after they were inoculated (left hindpaw; i.d.) with B16F10- or non-tumorigenic keratinocytes, cultured and calcium flux to ligands tested (ATP (10 μM), and capsaicin (1 μM)). Compared to neurons from keratinocytes-injected mice, the one from tumour-bearing mice showed increased sensitivity to capsaicin. (**e**) Naive DRG neurons (*Trpv1*^*cre*^*::CheRiff-eGFP*^*fl/WT*^) were cultured alone or in combination with B16F10-mCherry-OVA. After 48h, the cells were collected, FACS purified, and RNA sequenced. Hierarchical clustering of sorted neuron DEG show distinct groups of transcripts enriched in TRPV1^+^ neuron vs cancer-exposed TRPV1^+^ neuron populations. Pairwise comparison of naive TRPV1^+^ neuron vs cancer-exposed TRPV1^+^ neuron populations showing differentially expressed transcripts as a volcano plot (p<0.05). Among others, *Calca* (gene encoding for CGRP) was overexpressed in TRPV1^+^ (FACS-purified eGFP-expressing cells) neurons when co-cultured with B16F10-mCherry-OVA. Data are shown as representative image (**a**), mean ± S.E.M (**b–d**), or volcano plot (**e**). N are as follows: **a**: n = 4, **b**: neuron (n=8), neuron + keratinocytes (n = 7), neuron + B16F0 (n = 7), neuron + B16F10 (n = 7), **c**: n = 15/groups, **d**: keratinocytes inj. + ATP (n=5), B16F10 inj. + ATP (n=36), Keratinocytes inj. + caps (n = 6), B16F10 inj. + caps (n = 44), **e**: n = 4/groups. Experiments were independently repeated two (**d**) or three (**a–c**) times with similar results. Sequencing experiment was not repeated (**e**). P-values are shown in the figure and determined by one-way ANOVA post-hoc Bonferroni (**b–c**) or two-sided unpaired Student’s t-test (**d**). Scale bar = 100 μm (**a**). Source Data

To investigate this in more detail, we inoculated a GFP-expressing melanoma (B16F10-eGFP) cell line into *Nav1.8*^*cre*^*::tdTomato*^*fl/WT*^ mice (*Nav1.8* is also known as *Scn10a*). Twenty-two days after implantation, we found abundant Na_V_1.8^+^ nociceptor neurons around and within the tumour (Fig. 1a). RNA sequencing of samples from B16F10-bearing mice revealed that malignant and melanoma-infiltrating immune cells had no detectable levels of neuronal markers (*Nav1.8* or *Trpv1*), indicating that the Na_V_1.8 signal could be ascribed to tumour-infiltrating nerves (Extended Data Fig. 3). We next used an in vitro co-culture approach to assess whether malignant cells modulate the function of nociceptor neurons. When co-cultured, TRPV1^+^ nociceptors directly extended neurites towards the B16F10-eGFP melanoma cells, and the average length of neurites increased, whereas the overall neuronal arborization or branching decreased (Extended Data Fig. 4a–c). Together, these data indicate that nociceptor outgrowth is enhanced when in proximity to melanoma cells and that skin sensory neuron collaterals sprout directly into the tumour bed. Such tumour neo-innervation may be akin to cancer’s neoangiogenesis.

## Melanoma cells sensitize nociceptors

Given that melanoma promotes axonogenesis, leading to tumour innervation (Fig. 1a and Extended Data Fig. 2), we examined whether this physical proximity allows melanomas to modulate the sensitivity of the nociceptor. As nociceptor neurons detect signals from the local environment, we measured changes in calcium flux in response to sub-threshold concentrations of various noxious ligands. When nociceptors were cultured without melanoma cells, few responded to the ligands at the concentrations selected. However, the number of responsive neurons increased when they were co-cultured with B16F10 cells (Fig. 1b). Similarly, the amplitude of calcium flux responses to the ligands was greater in lumbar DRG neurons (L3–L5) that were collected ipsilateral to a 14-day tumour inoculation in mice, as compared to those collected from mice that were injected with non-tumorigenic keratinocytes (Extended Data Fig. 4d). Signals released from melanoma, therefore, heighten nociceptor sensitivity.

We next tested whether this neuronal hypersensitivity would lead to an increased release of immunomodulatory neuropeptides. In contrast to B16F10 cells alone, DRG neurons co-cultured with B16F10 cells (5 × 10^4^ cells, 96 h) actively release CGRP in the medium (Fig. 1c). These data prompted us to test whether exposure to melanoma alters the transcriptome of nociceptor neurons. To do so, we cultured naive DRG neurons (*Trpv1*^*cre*^*::-CheRiff-eGFP*^*fl/WT*^) alone or in combination with B16F10-mCherry-OVA cells. After 48 h, TRPV1^+^ nociceptors were purified by fluorescence-activated cell sorting (FACS) and RNA sequenced. Differentially expressed genes (DEGs) were calculated, and *Calca*—the gene that encodes CGRP—and the NGF receptor *Trka* (also known as *Ntrk1*)were found to be overexpressed in nociceptors that were exposed to cancer (Fig. 1d–e and Extended Data Fig. 4e). Overexpression of *Trka* may help to drive melanoma-induced hypersensitivity to pain, whereas CGRP, when released from activated nociceptors, may immunomodulate TILs.

**Fig. 2:**
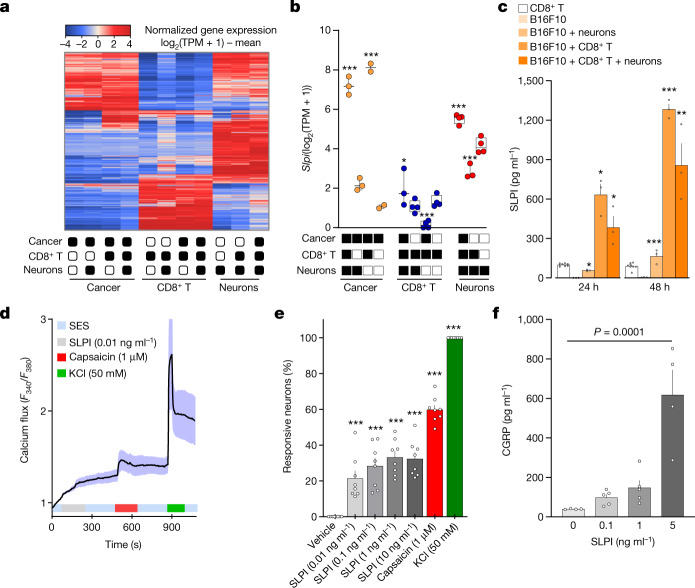
Cancer-secreted SLPI drives the release of CGRP by nociceptor neurons. **a**–**c**, Naive DRG neurons (*Trpv1*^*cre*^*::-CheRiff-eGFP*^*fl/WT*^), B16F10-mCherry-OVA cells and OVA-specific cytotoxic CD8^+^ T cells were cultured alone or in combination. After 48 h, the cells were collected, FACS purified and RNA sequenced. **a**, Hierarchical clustering of sorted neuron molecular profiles depicts distinct groups of transcripts enriched in each group. **b**, DEGs were calculated, and *Slpi* was found to be overexpressed in cancer cells when co-cultured with OVA-specific cytotoxic CD8^+^ T cells, DRG neurons or both populations. **c**, SLPI is secreted by B16F10-mCherry-OVA cells when co-cultured (24 h or 48 h) with naive DRG neurons and OVA-specific cytotoxic CD8^+^ T cells, with a maximal effect after 48 h. **d**–**f**, Using calcium microscopy, we found that SLPI (10 pg ml^−1^–10 ng ml^−1^) activated around 20% of cultured naive DRG neurons (**d**,**e**). Activation of cultured neurons (3 h) with SLPI also leads to significant release of CGRP (**f**). Data are shown as a heat map showing normalized gene expression (log_2_(1 + TPM) − mean (**a**), as box-and-whisters plots (as defined in Fig. 1b,c) (**b**) or as mean ± s.e.m. (**c**–**f**). *n* as follows: **a**,**b**: *n* = 2–4 per groups; **c**: *n* = 3 for all groups except CD8^+^ T cells (*n* = 8); **d**: *n* = 17; **e**: *n* = 8 per group; **f**: 0 ng ml^−1^ (*n* = 4), 0.1 ng ml^−1^ (*n* = 5), 1 ng ml^−1^ (*n* = 5), 5 ng ml^−1^ (*n* = 4). Experiments in **c**–**f** were independently repeated three times with similar results. The sequencing experiment was not repeated (**a**,**b**). *P* values were determined by one-way ANOVA with post-hoc Bonferroni (**b**,**e**,**f**) or two-sided unpaired Student’s *t*-test (**c**). *P ≤ 0.05, **P ≤ 0.01, and ***P ≤ 0.001. Source Data

**Extended Data Fig. 5:**
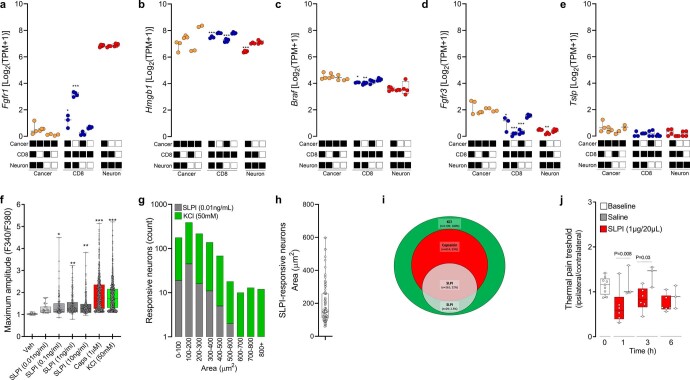
B16F10-secreted SLPI activates nociceptor neurons. (**a–e**) Naive DRG neurons (*Trpv1*^*cre*^*::CheRiff-eGFP*^*fl/WT*^), B16F10-mCherry-OVA, and OVA-specific cytotoxic CD8^+^ T cells were cultured alone or in combination. After 48h, the cells were collected, FACS purified, and RNA sequenced. DEGs were calculated, and *Fgfr1* (fibroblast growth factor receptor 1) was found to be overexpressed in OVA-specific cytotoxic CD8^+^ T cells when co-cultured with cancer cells and DRG neurons (**a**). Conversely, OVA-specific cytotoxic CD8^+^ T cells downregulates the expression of the pro-nociceptive factor *Hmgb1* (High–mobility group box 1; **b**), *Braf (****c****)*, as well as *Fgfr3 (****d****)* when co-cultured with B16F10-mCherry-OVA and DRG neurons. *Tslp* expression level was not affected in any of tested groups (**e**). (**f–i**) Using calcium microscopy, we probed whether SLPI directly activates cultured DRG neurons. We found that SLPI (0.01-10 ng/mL) induces a significant calcium influx in DRG neurons (**f**). SLPI-responsive neurons are mostly small-sized neurons (**g-h**; mean area = 151 μm^2^) and largely capsaicin-responsive (**i**; ~42%). (**j**) The right hindpaw of naive mice was injected with saline (20 μL) or SLPI (i.d., 1 μg/20 μL), and the mice’s noxious thermal nociceptive threshold was measured (0-6h). The ipsilateral paw injected with SLPI showed thermal hypersensitivity in contrast with the contralateral paw. Saline had no effect on the mice’s thermal sensitivity. Data are shown as box-and-whisker plots (runs from minimal to maximal values; the box extends from 25^th^ to 75^th^ percentile and the middle line indicates the median), for which individual data points are given (**a–f**, **h**, **j**), stacked bar graph on a logarithmic scale (**g**), and Venn Diagram (**i**). N are as follows: **a–e**: n = 2–4/groups, **f**: vehicle (n = 28), 10pg/ml (n = 28), 100 pg/ml (n = 132), 1,000 pg/ml (n = 191), 10 ng/ml (n = 260), capsaicin (n = 613), KCl (n = 1,139), **g**: 0-100 (SLPI=19; KCl=177), 100-200 (SLPI = 45; KCl = 390), 200-300 (SLPI = 16; KCl =216), 300-400 (SLPI=11; KCl = 138), 400-500 (SLPI = 5; KCl = 68), 500-600 (SLPI=2, KCl = 18), 600-700 (SLPI = 0; KCl = 10), 700-800 (SLPI=0; KCl=13), 800+ (SLPI = 0; KCl = 12), **h**: n = 98, **i**: KCl^+^=1139, KCl^+^Caps^+^=614, KCl^+^Caps^+^SLPI^+^=261, KCl^+^Caps^-^SLPI^+^=29, **j**: 0h (n = 9), SLPI at 1h (n = 6), saline at 1h (n = 3), SLPI at 3h (n = 6), saline at 3h (n=3), SLPI at 6h (n = 6), saline at 6h (n = 3). Experiments were independently repeated two (**j**) or three (**f–i**) times with similar results. Sequencing experiment was not repeated (**a–e**). P-values were determined by one-way ANOVA post-hoc Bonferroni (**a–f**); or two-sided unpaired Student’s t-test (**j**). P-values are shown in the figure or indicated by * for p ≤ 0.05; ** for p ≤ 0.01; *** for p ≤ 0.001. Source Data

To identify the mechanism through which melanoma sensitizes nociceptor neurons, we used a co-culture system designed to mimic the interactions that take place in the melanoma microenvironment. Type 1 (T_c1_)-stimulated (ex-vivo-activated by CD3 and CD28, IL-12 and anti-IL-4 for 48 h) OVA-specific cytotoxic CD8^+^ T cells (OT-I mice), naive DRG neurons (*Trpv1*^*cre*^*::CheRiff-eGFP*^*fl/WT*^) and B16F10-mCherry-OVA melanoma cancer cells were cultured alone or in combination. After 48 h, the cells were collected, purified by FACS and RNA sequenced, and DEGs were calculated. Among others, we found that *Slpi* (secretory leukocyte protease inhibitor) was overexpressed in the melanoma cancer cells when co-cultured with either DRG neurons (around 3.6-fold) or OVA-specific cytotoxic CD8^+^ T cells (around 270-fold), and when exposed to both populations (around 150-fold) (Fig. 2a,b and Extended Data Fig. 5a–e). We also found that B16F10-mCherry-OVA cells, when co-cultured with naive DRG neurons and OVA-specific cytotoxic CD8^+^ T cells, increased the secretion of SLPI into the culture medium, with this effect being maximal after 48 h (around 200-fold; Fig. 2c).

In addition to protecting epithelial cells from the activity of serine proteases, SLPI enhances the regeneration of transected retinal ganglion cell axons^23^ and the proliferation of neural stem cells^24^. Although these data provide evidence of the effect of SLPI on neurons, its role in nociception is unclear. To address this, we measured whether SLPI directly activates cultured DRG neurons using calcium microscopy. We found that SLPI (0.01–10 ng ml^−1^) activates around 20% of DRG neurons and that—consistent with these neurons being nociceptors—SLPI-sensitive neurons were mostly small (with a mean area of 151 µm^2^) capsaicin-responsive (around 90%) neurons (Fig. 2d,e and Extended Data Fig. 5f–i). Given that SLPI triggered calcium influx, we investigated whether this is the means by which B16F10 cells drive the release of CGRP from neurons (Fig. 1c). SLPI, when used at a concentration similar to that secreted by melanoma cells (Fig. 2c), induced the release of CGRP from cultured naive DRG neurons (Fig. 2f). Finally, we sought to test whether SLPI can drive pain hypersensitivity in vivo. When administered into the right hindpaw of naive mice, SLPI generated transient thermal hypersensitivity (Extended Data Fig. 5j).

**Fig. 3:**
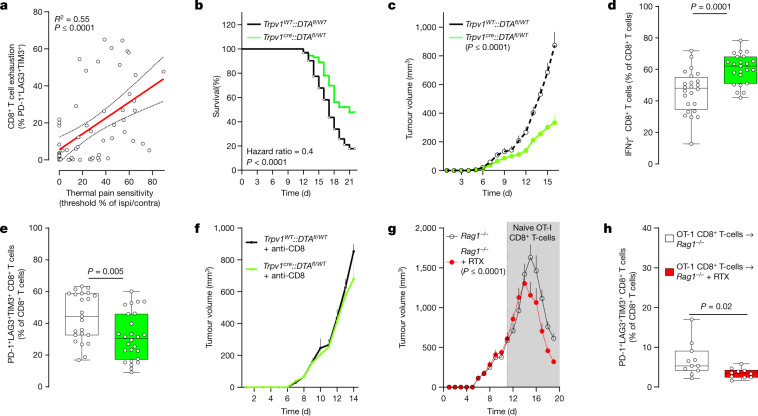
Genetic ablation of nociceptors safeguards anti-tumour immunity. **a**, Orthotopic B16F10-mCherry-OVA cells (2 × 10^5^ cells, i.d.) were injected into the left hindpaw of wild-type mice. As measured on day 13 after tumour inoculation, intratumoral CD8^+^ T cell exhaustion positively correlated with thermal hypersensitivity (*R*^2^ = 0.55, *P* ≤ 0.0001). The thermal pain hypersensitivity represents the withdrawal latency ratio of the ipsilateral paw (tumour-inoculated) to the contralateral paw. **b**, Orthotopic B16F10-mCherry-OVA (5 × 10^5^ cells, i.d.) were inoculated into the flank of eight-week-old male and female mice with sensory neurons intact (*Trpv1*^*WT*^*::DTA*^*fl/WT*^) or ablated (*Trpv1*^*cre*^*::DTA*^*fl/WT*^). The median length of survival was increased by around 250% in nociceptor-ablated mice (measured until 22 days after inoculation). **c**–**f**, Sixteen days after tumour inoculation, sensory-neuron-ablated mice have reduced tumour growth (**c**) and increased tumour infiltration of IFNγ^+^ CD8^+^ T cells (**d**), and the proportion of PD-1^+^LAG3^+^TIM3^+^ CD8^+^ T cells is decreased (**e**). This reduction in B16F10-mCherry-OVA (5 × 10^5^ cells, i.d.) tumour volume was absent in nociceptor-ablated mice whose CD8^+^ T cells were systemically depleted (**f**; assessed until day 14; anti-CD8, 200 μg per mouse, i.p., every 3 days). **g**,**h**, To chemically deplete their nociceptor neurons, *Rag1*^−/^^−^ mice were injected with RTX. Twenty-eight days later, the mice were inoculated with B16F10-mCherry-OVA (5 × 10^5^ cells, i.d.). RTX-injected mice that were adoptively transferred with naive OVA-specific CD8^+^ T cells (i.v., 1 × 10^6^ cells, when tumour reached around 500 mm^3^) showed reduced tumour growth (**g**; assessed until day 19) and exhaustion (**h**) compared to vehicle-exposed *Rag1*^−/^^−^ mice. Data are shown as a linear regression analysis ± s.e. (**a**), as a Mantel–Cox regression (**b**), as mean ± s.e.m. (**c**,**f**,**g**) or as box-and-whisker plots (as defined in Fig. 1b,c), for which individual data points are given (**d**,**e**,**h**). *n* as follows: **a**: *n* = 60; **b**: intact (*n* = 62), ablated (*n* = 73); **c**: intact (*n* = 20), ablated (*n* = 25); **d**: intact (*n* = 24), ablated (*n* = 23); **e**: intact (*n* = 23), ablated (*n* = 26); **f**: intact + anti-CD8 (*n* = 10), ablated + anti-CD8 (*n* = 8); **g**: vehicle (*n* = 12), RTX (*n* = 10); **h**: vehicle (*n* = 11), RTX (*n* = 10). Experiments were independently repeated two (**a**,**f**–**h**) or six (**b**–**e**) times with similar results. *P* values were determined by simple linear regression analysis (**a**), Mantel–Cox regression (**b**), two-way ANOVA with post-hoc Bonferroni (**c**,**f**,**g**) or two-sided unpaired Student’s *t*-test (**d**,**e**,**h**). Source Data

Melanoma-secreted SLPI acts on nociceptors to trigger calcium influx, neuropeptide release and thermal hypersensitivity, which indicates that these sensory neurons detect and react to the presence of cancer cells. Whether this gives the malignant cells a functional advantage over the host cells remains unknown. To assess this, we implanted B16F10-mCherry-OVA cells (intradermally (i.d.), 2 × 10^5^ cells) into the hindpaw of eight-week-old male and female mice. We found that mice with larger tumours had a higher proportion of intratumoral PD-1^+^LAG3^+^TIM3^+^ CD8^+^ T cells and greater hypersensitivity to thermal pain (not shown). Notably, heightened sensitivity to thermal pain positively correlated (*n* = 60; *R*^2^ = 0.55, *P* < 0.0001) with increased frequency in intratumoral PD-1^+^LAG3^+^TIM3^+^ CD8^+^ T cells (Fig. 3a; measured on day 13 after implantation).

## Melanoma-innervating nociceptors control tumour growth

**Extended Data Fig. 6:**
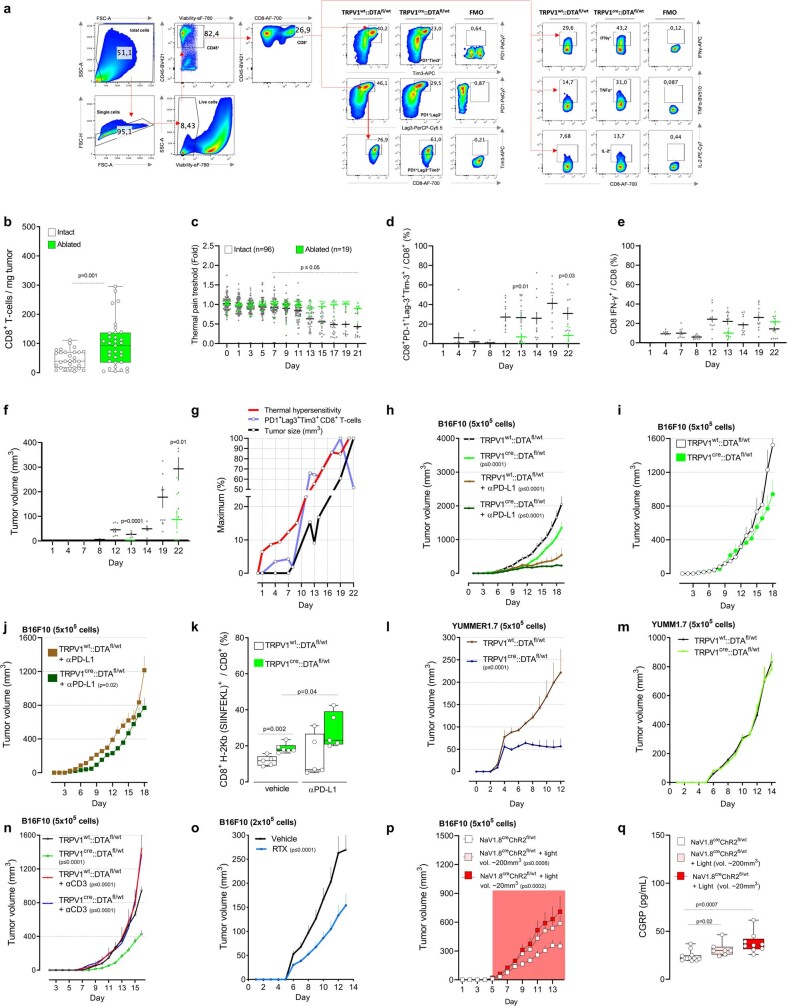
Nociceptor ablation reduces the exhaustion of intratumoral CD8^+^ T cells. (**a-b**) Orthotopic B16F10-mCherry-OVA (5x10^5^ cells; i.d.) cells were injected to nociceptor intact (*Trpv1*^*WT*^*::DTA*^*fl/WT*^) and ablated (*Trpv1*^*cre*^*::DTA*^*fl/WT*^) mice. Sixteen days post-B16F10-mCherry-OVA cells inoculation (5x10^5^ cells; i.d.), tumour-infiltrating CD8^+^ T cells were immunophenotyped (**a**) and were found to be more numerous in sensory neuron depleted tumours (**b**). (**c-g**) Orthotopic B16F10-mCherry-OVA (2x10^5^ cells; i.d.) cells were injected into the left hindpaw paw of nociceptor intact (n = 96; *Trpv1*^*WT*^*::DTA*^*fl/WT*^) or ablated (n = 18; *Trpv1*^*cre*^*::DTA*^*fl/WT*^) mice. When compared to their baseline threshold, littermate control mice showed significant thermal hypersensitivity on day 7, an effect that peaks on day 21 (**c**). In these mice, intratumoral frequency of PD-1^+^LAG3^+^TIM3^+^ (**d**) and IFNγ^+^ (**e**) CD8^+^ T cells increased 12 days post tumour inoculation, an effect that peaked on day 19. Finally, B16F10 tumour volume peaked on day 22 (**f**). When compared with littermate control mice, sensory neuron ablated mice inoculated with B16F10 cells showed no thermal pain hypersensitivity (**c**), reduced intratumoral frequency of PD-1^+^LAG3^+^TIM3^+^ CD8^+^ T cells (**d**) and tumour volume (**f**). In littermate control mice, thermal pain hypersensitivity (day 7) precedes the increase in intratumoral frequency of PD-1^+^LAG3^+^TIM3^+^ CD8^+^ T cells (day 12), and significant tumour growth (day 12; **g**). (**h**) Orthotopic B16F10-mCherry-OVA cells (5x10^5^ cells; i.d.) were inoculated into 8-week-old male and female sensory neuron intact or ablated mice. The mice were treated with αPD-L1 (6 mg/kg, i.p.; days 7, 10, 13, 16 post tumour inoculation) or its isotype control. On day 19, αPD-L1 potentiated the nociceptor ablation mediated reduction in B16F10-OVA tumour volume. (**i–k**) Orthotropic B16F10-mCherry-OVA cells (5x10^5^ cells, i.d.) were injected into a cohort of nociceptor neuron-ablated mice 3 days prior to the injection given to nociceptor intact mice. Mice from each group with similar tumour size (~85mm^3^) were selected and exposed to αPD-L1 (6 mg/kg, i.p.) once every 3 days for a total of 9 days. Eighteen days post tumour inoculation, we found that αPD-L1-reduced tumour growth was higher (~47%) in nociceptor-ablated mice than was observed in nociceptor-intact mice (~32%; **i–j**). In addition, nociceptor ablation increased the proportion of intratumoral tumour-specific (**k**; defined as H-2Kb^+^) CD8^+^ T cells. These differences were further enhanced by αPD-L1 treatment (**i–k**). (**l–m**) Sensory neurons ablation (*Trpv1*^*cre*^*::DTA*^*fl/WT*^) decreased growth of YUMMER1.7 cells (5×10^5^ cells; i.d.) an immunogenic version of a Braf^V600E^Cdkn2a^−/−^Pten^−/−^ melanoma cell line (**l**; assessed until day 12). The non-immunogenic YUMM1.7 cell line (5×10^5^ cells; i.d.; assessed until day 14) cells were injected to nociceptor intact (*Trpv1*^*WT*^*::DTA*^*fl/WT*^) and ablated mice (*Trpv1*^*cre*^*::DTA*^*fl/WT*^). Nociceptor ablation had no effect on YUMM1.7 growth (**m**). (**n**) Orthotopic B16F10-mCherry-OVA (5x10^5^ cells; i.d.) cells were injected to nociceptor intact (*Trpv1*^*WT*^*::DTA*^*fl/WT*^) and ablated mice (*Trpv1*^*cre*^*::DTA*^*fl/WT*^). The reduction in B16F10-mCherry-OVA (5×10^5^ cells; i.d.) tumour growth observed in nociceptors ablated mice was absent following systemic CD3 depletion (assessed until day 15; αCD3, 200 μg/mouse; i.p.; every 3 days). (**o**) To deplete their nociceptor neurons, C57BL6J mice were injected with RTX (s.c., 30, 70, 100 μg/kg) and were subsequently (28 days later) inoculated with B16F10-mCherry-OVA (2×10^5^ cells). RTX-injected mice showed reduced tumour growth when compared to vehicle-exposed mice (assessed until day 13). (**p–q**) Orthotopic B16F10-mCherry-OVA (5×10^5^ cells; i.d.) cells were injected to light-sensitive mice (*Nav1.8*^*cre*^*::ChR2*^*fl/WT*^). As opposed to unstimulated mice, the optogenetic activation (3.5 ms, 10Hz, 478nm, 60 mW, giving approx. 2-6 mW/mm^2^ with a 0.39-NA fibre placed 5–10 mm from the skin, 20 min) of tumour-innervating nociceptor neurons, when started once B16F10 tumours were visible (~20 mm^3^) or well established (~200 mm^3^), resulted in enhanced tumour growth (**p**, as measured until day 14) and intratumoral CGRP release (**q**). Data are shown as FACS plot (**a**; depict the gating strategy used in fig. 3d,e), as box-and-whisker plots (runs from minimal to maximal values; the box extends from 25^th^ to 75^th^ percentile and the middle line indicates the median) for which individual data points are given (**b**,**k**,**q**), scatter dot plot (**c–f**), percentage change from maximal thermal hypersensitivity, intratumoral frequency of PD-1^+^LAG3^+^TIM3^+^ CD8^+^ T cells and tumour volume (**g**), or mean ± S.E.M (**h–j**, **l–p**). N are as follows: **a–b**: intact (n = 29), ablated (n = 33), **c**: intact (n = 96), ablated (n = 19), **d**: intact (n = 92), ablated (n = 15), **e**: intact (n = 96), ablated (n = 15), **f**: intact (n = 96), ablated (n = 16), **g**: n=96, **h**: intact (n = 9), ablated (n = 10), intact+αPD-L1 (n = 9), ablated+αPD-L1 (n = 8), **i**: intact (n = 14), ablated (n = 4), **j**: intact+αPD-L1 (n = 12), ablated+αPD-L1 (n = 12), **k**: intact (n = 5), ablated (n = 6), intact+αPD-L1 (n = 5), ablated+αPD-L1 (n = 5), **l**: intact (n = 8), ablated (n = 11), **m**: intact (n = 6), ablated (n = 13), **n**: intact (n = 5), ablated (n = 5), intact+αCD3 (n = 6), ablated+αCD3 (n = 5), **o**: vehicle (n = 11), RTX (n = 10), **p**: *Nav1.8*^*cre*^*::ChR2*^*fl/WT*^ (n = 12), *Nav1.8*^*cre*^*::ChR2*^*fl/WT*^ + Light (vol. ~200 mm^3^) (n = 8), *Nav1.8*^*cre*^*::ChR2*^*fl/WT*^ + Light (vol. ~20 mm^3^) (n = 8), **q**: *Nav1.8*^*cre*^*::ChR2*^*fl/WT*^ (n = 12), *Nav1.8*^*cre*^*::ChR2*^*fl/WT*^ + Light (vol. ~200 mm^3^) (n = 7), *Nav1.8*^*cre*^*::ChR2*^*fl/WT*^ + Light (vol. ~20 mm^3^) (n = 9). Experiments were independently repeated two (**c–g**), three (**h–q**) or six (**a**,**b**) times with similar results. P-values are shown in the figure and determined by two-sided unpaired Student’s t-test (**b–f**, **k**,**q**), or two-way ANOVA post-hoc Bonferroni (**h–j**, **l–p**). Source Data

The expression of adrenergic and cholinergic axon markers in tumours correlates with poor clinical outcome^2^. Gastric tumour denervation limits growth and patients who have undergone vagotomy have lower rates of mortality from intestinal cancer^16,25,26^. To investigate the nature of the three-way interaction between cancer, nociceptors and CD8^+^ T cells, we next used a syngeneic mouse model of triple-negative melanoma, which is an established model of immunosurveillance^9^. B16F10-mCherry-OVA cells were inoculated (i.d., 5 × 10^5^ cells) into eight-week-old male and female nociceptor-ablated (*Trpv1*^*cre*^*::DTA*^*fl/WT*^) or intact (littermate control; *Trpv1*^*WT*^*::DTA*^*fl/WT*^) mice. In nociceptor-ablated male and female mice, the median length of survival increased by 2.5-fold (evaluated until day 22; Fig. 3b). In another set of mice that were analysed 16 days after tumour inoculation, we found that genetic ablation of nociceptors reduced tumour growth (Fig 3c). In addition, nociceptor-ablated mice showed an increase in the total number and relative frequency of cytotoxic (IFNγ^+^, TNF^+^or IL-2^+^) tumour-infiltrating CD8^+^ T cells, but a reduced proportion of PD-1^+^LAG3^+^TIM3^+^ CD8^+^ T cells (Fig. 3d,e and Extended Data Fig. 6a,b).

Up to this point, our data suggest that nociceptor neurons are an upstream driver of intratumoral PD-1^+^LAG3^+^TIM3^+^ CD8^+^ T cells. To assess whether this is indeed the case, we mapped out the kinetics of thermal pain hypersensitivity, increased frequency in intratumoral PD-1^+^LAG3^+^TIM3^+^ CD8^+^ T cells and tumour growth. When compared to their baseline threshold and to that of sensory-neuron-ablated mice (*Trpv1*^*cre*^*::DTA*^*fl/WT*^; *n* = 19), eight-week-old littermate control mice (*Trpv1*^*WT*^*::DTA*^*fl/WT*^; *n* = 96) that were inoculated with B16F10-mCherry-OVA (left hindpaw, i.d., 2 × 10^5^ cells) showed significant thermal hypersensitivity on day 7, an effect that peaked on day 21 (Extended Data Fig. 6c). In these mice, the intratumoral frequency of PD-1^+^LAG3^+^TIM3^+^ (Extended Data Fig. 6d) or IFNγ^+^ (Extended Data Fig. 6e) CD8^+^ T cells was significantly increased 12 days after tumour inoculation and peaked on day 19. Finally, B16F10-mCherry-OVA tumour volume peaked on day 22 (Extended Data Fig. 6f). Altogether, these data show that thermal hypersensitivity precedes any significant exhaustion of intratumoral CD8^+^ T cells by around five days and that pain hypersensitivity develops before the tumour is measurable using a digital caliper (Extended Data Fig. 6g).

Blocking the activity of immune checkpoint proteins releases a cancer-cell-induced ‘brake’ on the immune system, thereby increasing its ability to eliminate tumours^6,8–10^. Immune checkpoint inhibitors (ICIs), including those that target PD-L1, improve clinical outcomes in patients with metastatic melanoma^8^; however, the efficacy of ICIs varies considerably among patients, half of whom will not benefit^27^. We set out to assess whether the presence (*Trpv1*^*WT*^*::DTA*^*fl/WT*^) or absence (*Trpv1*^*cre*^*::DTA*^*fl/WT*^) of tumour-innervating nociceptor neurons would affect responsiveness to treatment with anti-PD-L1. Anti-PD-L1 (intraperitoneally (i.p.), days 7, 10, 13 and 16) was given either to mice whose tumour cells (B16F10-mCherry-OVA, i.d., 5 × 10^5^ cells) were inoculated on the same day, or to mice with established tumours (around 85 mm^3^; achieved by inoculating *Trpv1*^*cre*^*::DTA*^*fl/WT*^ around 3 days before). In both scenarios, ablation of nociceptors increased the anti-PD-L1-mediated reduction in tumours and the infiltration of tumour-specific CD8^+^ T cells (Extended Data Fig. 6h–k).

To test whether the reduction in tumour growth that was observed in the absence of nociceptor neurons depends on their action on immune cells, we compared the respective effects of nociceptors on the growth of an immunogenic and a non-immunogenic isogenic melanoma model. YUMMER1.7 is a highly immunogenic derivative of the *Braf*^*V600E*^*Cdkn2a*^−/−^*Pten*^−/−^ cell line modified by ultraviolet (UV) exposure, and provides a clinically relevant model of melanoma^28^. As in the case of B16F10-OVA, ablation of nociceptors decreased the growth of tumours (Extended Data Fig. 6l) and reduced their frequency in intratumoral PD-1^+^LAG3^+^TIM3^+^ CD8 T cells. while increasing their number and cytotoxic potential (IFNγ^+^ or TNF^+^; not shown). By contrast, YUMM1.7 (the parental and non-immunogenic^29^ counterpart of YUMMER1.7) showed similar tumour growth (Extended Data Fig. 6m) and a similar frequency of intratumoral PD-1^+^LAG3^+^TIM3^+^ CD8^+^ T cells in both the presence and the absence of nociceptors (not shown).

Next, we assessed whether these differences were due to nociceptor neurons directly modulating intratumoral T cells. We observed no major changes in tumour growth between nociceptor-intact and nociceptor-ablated mice after systemic depletion of CD8^+^ (Fig. 3f) or CD3^+^ (Extended Data Fig. 6n) T cells. Although chemoablation of nociceptor neurons with resiniferatoxin (RTX) reduced tumour growth in B16F10-inoculated wild-type mice (Extended Data Fig. 6o), we found that naive OT-I CD8^+^ T cells enhanced tumour shrinkage when transplanted in RTX-exposed *Rag1*^−/^^−^ mice (Fig. 3g). In doing so, the chemoablation of nociceptor neurons shielded the naive OT-I CD8^+^ T cells from undergoing exhaustion (Fig. 3h). These data imply that the slower tumour growth found in *Trpv1*^*cre*^*::DTA*^*fl/WT*^ and RTX-exposed mice depends on the modulation of CD8^+^ T cells by nociceptors.

Optogenetic activation of skin nociceptor neurons triggers the antidromic release of neuropeptides that mediate anticipatory immunity against microorganisms^30^ and potentiate skin immunity^31^. We used transdermal illumination to stimulate tumour-innervating Na_V_1.8^+^ channelrhodopsin-expressing neurons (*Nav1.8*^*cre*^*::ChR2*^*fl/WT*^). Daily stimulation with blue light enhanced the growth of B16F10 when exposure began in mice bearing visible (around 20 mm^3^) or well-established (around 200 mm^3^) tumours (Extended Data Fig. 6p). This increase in tumour volume was also linked to an increase in the intratumoral levels of CGRP, confirming the engagement of pain-transmitting neurons (Extended Data Fig. 6q). Laser exposure had no effect on tumour growth in light-insensitive mice (*Nav1.8*^*WT*^*::ChR2*^*fl/WT*^; not shown).

**Extended Data Fig. 7:**
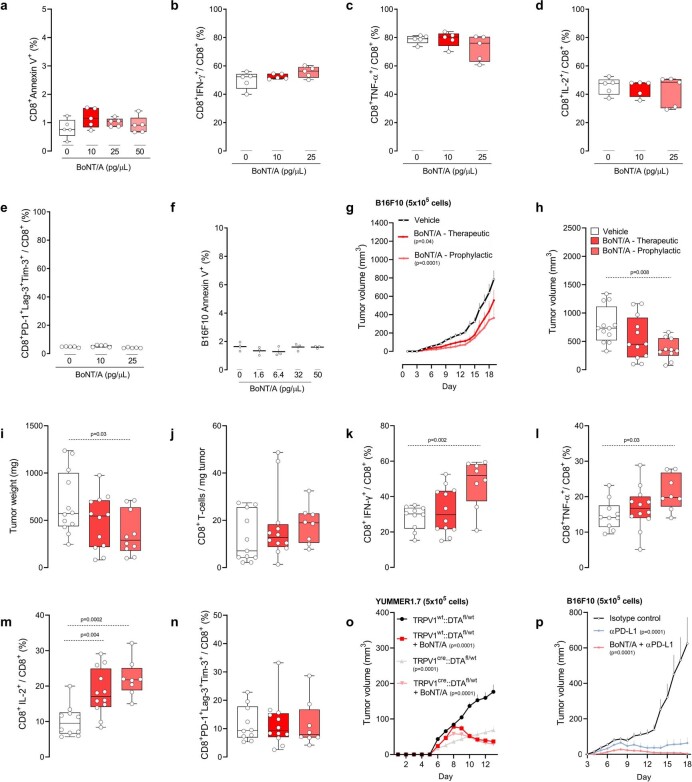
BoNT/A silencing of B16F10-innervating neurons decreases tumour growth. (**a–e**) Splenocytes-isolated CD8^+^ T cells from naive C57BL6J mice were cultured under T_c1_-stimulating conditions (*ex vivo* activated by CD3 and CD28, IL-12, and anti-IL4) for 48h. The cells were then exposed to BoNT/A (10–50 pg/μL) for 24h; effects on apoptosis, exhaustion, and activation were measured by flow cytometry. When compared to vehicle-exposed cells, BoNT/A did not affect the survival (**a**) of cultured cytotoxic CD8^+^ T cells, nor their relative expression of IFNγ^+^ (**b**), TNF^+^ (**c**), IL-2^+^ (**d**) and PD-1^+^LAG3^+^TIM3^+^ (**e**). (**f**) B16F10 (1x10^5^ cells) were cultured for 24h and subsequently exposed to BoNT/A (1.6-50 pg/μL) or its vehicle for an additional 24h. BoNT/A did not trigger B16F10 cells apoptosis, as measured by the mean fluorescence intensity of Annexin V. (**g–n**) One and three days prior to tumour inoculation (*defined as prophylactic*), the skin of 8-week-old male and female mice was injected with BoNT/A (25 pg/μL; i.d.) or its vehicle. One day after the last injection, orthotopic B16F10-mCherry-OVA (5x10^5^ cells; i.d.) were inoculated into the area pre-exposed to BoNT/A. In another group of mice, BoNT/A was administered (25 pg/μL; i.d.) one and three days after the tumour reached a volume of ~200mm3 *(defined as therapeutic*). The effect of neuron silencing on tumour size and tumour-infiltrating CD8^+^ T cell exhaustion was measured. Nineteen days post tumour inoculation, we found that the tumour volume (**g**,**h**) and weight (**i**) were reduced in mice treated with BoNT/A (*Prophylactic group*). In parallel, we found that silencing tumour-innervating neurons increased the proportion of IFNγ^+^ (**k**), TNF^+^ (**l**), and IL-2^+^ (**m**) CD8^+^ T cells. BoNT/A had no effect on the total number of intratumoral CD8 T cells (**j**) or the relative proportion of PD-1^+^LAG3^+^TIM3^+^ (**n**) CD8^+^ T cells. (**o**) One and three days prior to tumour inoculation, the skin of 8-week-old male and female sensory neuron-intact or ablated mice was injected with BoNT/A (25 pg/μL; i.d.) or its vehicle. One day following the last injection, orthotopic YUMMER1.7 cells (5×10^5^ cells; i.d.) were inoculated into the area pre-exposed to BoNT/A. The effects of nociceptor neuron ablation on tumour size and volume were measured. Thirteen days post tumour inoculation, we found that the tumour growth was lower in mice treated with BoNT/A or in sensory neuron-ablated mice. BoNT/A had no additive effects when administered to sensory neuron-ablated mice. (**p**) One and three days prior to tumour inoculation, the skin of 8-week-old male and female mice was injected with BoNT/A (25 pg/μL; i.d.) or its vehicle. One day following the last injection, orthotopic B16F10-mCherry-OVA cells (5×10^5^ cells; i.d.) were inoculated into the area pre-exposed to BoNT/A. On days 7, 10, 13 and 16 post tumour inoculation, the mice were exposed to αPD-L1 (6 mg/kg, i.p.) or its isotype control. Eighteen days post tumour inoculation, we found that neuron silencing using BoNT/A potentiated αPD-L1-mediated tumour reduction. Data are shown as box-and-whisker plots (runs from minimal to maximal values; the box extends from 25^th^ to 75^th^ percentile and the middle line indicates the median), for which individual data points are given (**a–f; h–n**) or as mean ± S.E.M (**g**,**o**,**p**). N are as follows: **a-e**: n = 5/groups, **f**: n = 3/groups, **g–i**: vehicle (n = 12), BoNT/A therapeutic (n = 12), BoNT/A prophylactic (n = 10), **j**: vehicle (n = 11), BoNT/A therapeutic (n = 12), BoNT/A prophylactic (n = 8), **k–n**: vehicle (n = 10), BoNT/A therapeutic (n = 12), BoNT/A prophylactic (n = 8), **o**: intact + vehicle (n = 9), ablated + vehicle (n = 8), intact + BoNT/A (n = 10), ablated + BoNT/A (n = 8), **p**: vehicle (n = 7), αPD-L1 (n = 8), αPD-L1 + BoNT/A (n = 7). Experiments were independently repeated two (**a–f**, **o–p**) or four (**g–n**) times with similar results. P-values are shown in the figure and determined by one-way ANOVA posthoc Bonferonni (**a–f**, **h–n**) or two-way ANOVA post-hoc Bonferroni (**g**,**o**,**p**). Source Data

The neonatal or embryonic ablation of neuronal subsets may lead to compensatory changes. To circumvent this possibility, we silenced neurons using botulinum neurotoxin A (BoNT/A), a neurotoxic protein produced by *Clostridium botulinum*, which acts by cleaving SNAP25 (ref. ^32^). BoNT/A causes a long-lasting (20 days) abolition of neurotransmitter release from skin-innervating neurons^33^. BoNT/A reduces tumour growth in prostate cancer^2^ and blocks nociceptor-mediated modulation of neutrophils during skin infection^33^. BoNT/A does not affect the function of cultured B16F10 or CD8^+^ T cells in vitro (Extended Data Fig. 7a–f). When BoNT/A (25 pg μl^−1^, 50 µl, five i.d. sites) was administered one and three days before the B16F10-OVA cell inoculation, it reduced subsequent tumour growth and preserved the cytotoxic potential of intratumoral CD8^+^ T cells (Extended Data Fig. 7g–n; as measured 18 days after inoculation). Pre-treatment with BoNT/A also reduced the growth of YUMMER1.7 tumours and enhanced anti-PD-L1-mediated tumour regression (Extended Data Fig. 7o,p). When administered to mice with established tumours (around 200 mm^3^), BoNT/A had limited efficacy (Extended Data Fig. 7g–n). BoNT/A also did not affect tumour growth when given to mice in which TRPV1^+^ nociceptor neurons were genetically ablated (Extended Data Fig. 7o), which suggests that its anti-tumour effectiveness depends on the presence of tumour-innervating nociceptor neurons.

**Extended Data Fig. 8:**
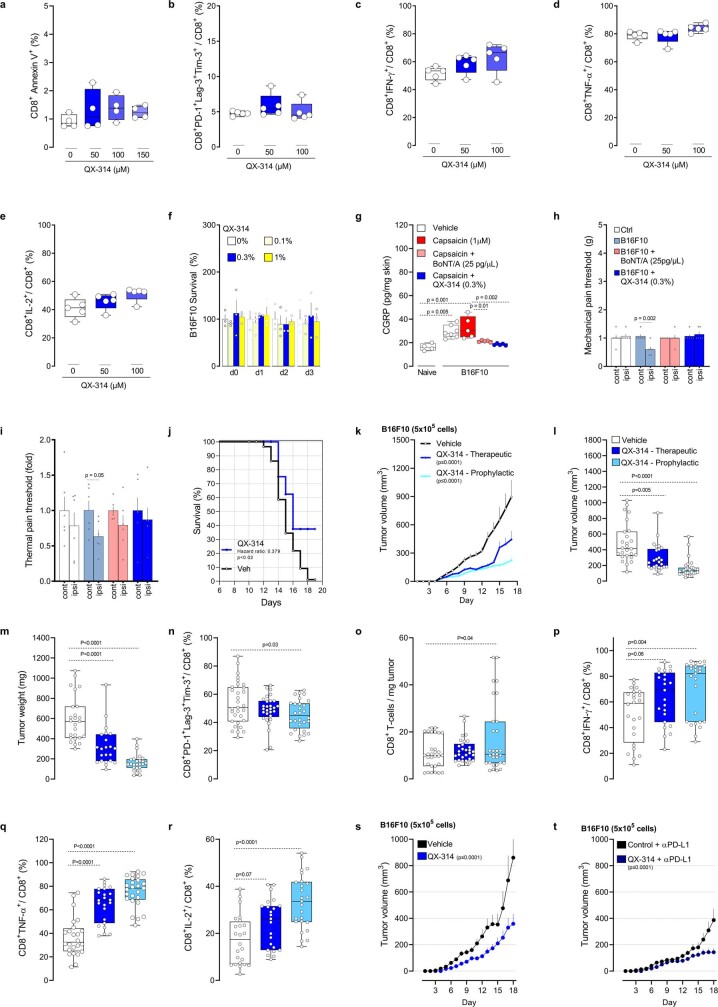
QX-314 silencing of B16F10-innervating neurons reduces tumour growth. (**a–e**) Splenocytes-isolated CD8^+^ T cells from naive C57BL6J mice were cultured under T_c1_-stimulating conditions (*ex vivo* activated by CD3 and CD28, IL-12, and anti-IL4) for 48h. The cells were then exposed to QX-314 (50–150 μM) for 24h, effects on apoptosis, exhaustion and activation were measured by flow cytometry. When compared to vehicle-exposed cells, QX-314 did not affect the survival of cultured cytotoxic CD8^+^ T cells (**a**), nor their relative expression of PD-1^+^LAG3^+^TIM3^+^ (**b**), IFNγ^+^ (**c**), TNF^+^ (**d**) and IL-2^+^ (**e**). (**f**) B16F10 (1x10^5^ cells) were cultured for 24h. The cells were then exposed or not to QX-314 (0.1-1%) for an additional 24-72h, and cell count was analysed by bright-field microscopy. QX-314 did not affect B16F10 cells’ survival, as measured by relative cell count changes (at each time point) in comparison to vehicle-exposed cells. (**g–i**) One and three days prior to tumour inoculation, 8-week-old male and female wild-type mice’s right hindpaws or flanks were injected with BoNT/A (25 pg/μL; i.d.) or its vehicle. On the following day, orthotopic B16F10 cells (**g**: 5x10^5^ cells; i.d.; **h–i**: 2x10^5^ cells; i.d.) were inoculated into the area pre-exposed to BoNT/A. Starting one day post inoculation, QX-314 (0.3%) or its vehicle was administered (i.d.) once daily in another group of mice. The effects of sensory neuron silencing were tested on neuropeptide release (**g**), as well as mechanical (**h**) and thermal pain hypersensitivity (**i**). First, CGRP levels were increased in B16F10 tumour surrounding skin explant (assessed on day 15) in comparison to control skin; an effect further enhanced by capsaicin (1 μM; 3h) but was absent in skin pre-treated with BoNT/A (25 pg/μL) or QX-314 (0.3%; **g**). We also found that B16F10 injection induced mechanical (**h**) and thermal pain hypersensitivities (**i**) fourteen days post tumour inoculation. These effects were stopped by sensory neuron silencing with QX-314 or BoNT/A (**h–i**). (**j**) Orthotopic B16F10-mCherry-OVA cells (5x10^5^ cells; i.d.) were inoculated into 8-week-old male and female mice. Starting one day post inoculation, QX-314 (0.3%; i.d.; 5 sites) was injected once daily around the tumour. The effect of nociceptor neuron silencing on tumour size and tumour-infiltrating CD8^+^ T cell exhaustion was measured. We found that silencing tumour-innervating neurons increased the mice’s median length of survival (~270% Mantel–Haenszel hazard ratio; measured on day 19). (**k–r**) Orthotopic B16F10-mCherry-OVA cells (5x10^5^ cells; i.d.) were inoculated into 8-week-old male and female mice. Starting one day post inoculation (*defined as prophylactic*), In other groups of mice, QX-314 daily injection started once the tumour reached a volume of ~200mm^3^ (*defined as therapeutic*). As measured seventeen days post tumour inoculation, silencing tumour innervation also decreased tumour volume (**k**,**l**) and weight (**m**), as well as the relative proportion of PD-1^+^LAG3^+^TIM3^+^ (**n**) CD8^+^ T cells. QX-314 treatment also increased the total number of intratumoral CD8^+^ T cells (**o**), as well as relative proportion of IFNγ^+^ (**p**), TNF^+^ (**q**), and IL-2^+^ (**r**) CD8^+^ T cells. (**s–t**) Orthotropic B16F10-mCherry-OVA cells (5x10^5^ cells, i.d.) were injected into mice treated with QX-314 (0.3%; i.d.) 2-3 days prior to being injected into vehicle-exposed mice. Mice from each group with similar tumour size (~100mm^3^) were selected and exposed to αPD-L1 (6 mg/kg, i.p.) once every 3 days for a total of 9 days. Eighteen days post tumour inoculation, we found that αPD-L1-reduced tumour growth was higher (~61%) in nociceptor silenced mice than was observed in isotype vehicle-exposed mice (~49%; **s-t**). Data are shown as box-and-whisker plots (runs from minimal to maximal values; the box extends from 25^th^ to 75^th^ percentile and the middle line indicates the median), for which individual data points are given (**a–e**, **g**, **l–r**), as mean ± S.E.M (**f**,**h**,**i**,**k**,**s**,**t**), or as Mantel–Cox regression analysis (**j**). N are as follows: **a**: n = 4/groups, **b–e**: n = 5/groups, **f**: n = 3/groups, **g**: naïve (n = 4), vehicle (n = 7), B16F10+vehicle (n = 5), B16F10+BoNT/A (n = 5), B16F10+QX-314 (n = 5), **h–i**: n = 6/groups, **j**: vehicle (n = 89), QX-314 (n = 12), **k**: vehicle (n = 21), QX-314 prophylactic (n = 21), QX-314 therapeutic (n = 17), **l**: vehicle (n = 26), QX-314 therapeutic (n = 26), QX-314 prophylactic (n = 28), **m**: vehicle (n = 25), QX-314 therapeutic (n = 22), QX-314 prophylactic (n = 25), **n**: vehicle (n = 31), QX-314 therapeutic (n = 29), QX-314 prophylactic (n = 28), **o**: n = 30/groups, **p–r**: vehicle (n = 24), QX-314 therapeutic (n = 23), QX-314 prophylactic (n = 25), **s**: vehicle (n = 9), QX-314 (n = 13), **t**: vehicle + αPD-L1 (n = 18), QX-314 + αPLD1 (n = 13). Experiments were independently repeated two (**a–i**, **s–t**) or four (**j–r**) times with similar results. P-values are shown in the figure and determined by one-way ANOVA posthoc Bonferonni (**a–g**, **l–r**), two-sided unpaired Student’s t-test (**h–i**), Mantel–Cox regression (**j**), or two-way ANOVA posthoc Bonferroni (**k**, **s–t**). Source Data

We next tested the anti-tumour efficacy of a proven nociceptor-selective silencing strategy^34^. This protocol uses large-pore ion channels (TRPV1) as cell-specific drug-entry ports to deliver QX-314—a charged and membrane-impermeable form of lidocaine—to block voltage-gated sodium (Na_V_) channels. During inflammation, similar to what we observed in tumour microenvironments, these large-pore ion channels open, which allows QX-314 to permeate the neurons and results in a long-lasting electrical blockade^17^. Although QX-314 did not affect cultured B16F10-mCherry-OVA cells or CD8^+^ T cell function in vitro (Extended Data Fig. 8a–f), we confirmed that it silences tumour-innervating nociceptors in vivo, as shown by reduced B16F10-induced release of CGRP and pain hypersensitivity (Extended Data Fig. 8g–i). We found that vehicle-exposed B16F10-mCherry-OVA-bearing mice succumbed at a 2.7-fold higher rate (*P* ≤ 0.02) than QX-314-exposed mice (Extended Data Fig. 8j; measured until day 19). As observed 17 days after tumour inoculation, QX-314-mediated silencing of sensory neurons (0.3%; daily i.d., surrounding the tumour) reduced melanoma growth and limited the exhaustion of intratumoral CD8^+^ T cells (Extended Data Fig. 8k–n). Nociceptor silencing also increased the intratumoral numbers of CD8^+^ T cells and preserved their cytotoxic potential (IFNγ^+^ or TNF^+^) as well as their proliferative capacity (IL-2^+^; Extended Data Fig. 8o–r). Similar to what was observed in nociceptor-ablated mice (Extended Data Fig. 6j–j), silencing tumour-innervating neurons with QX-314 enhanced anti-PD-L1-mediated tumour regression (Extended Data Fig. 8s,t). When administered to mice with an established (around 200 mm^3^) B16F10-mCherry-OVA tumour, QX-314 still reduced tumour growth and preserved the anti-tumour capacity of CD8^+^ T cells (Extended Data Fig. 8k–r), suggesting that it could be used as a therapeutic agent in cancer.

## CGRP attenuates the activity of RAMP1^+^ CD8^+^ T cells

**Fig. 4:**
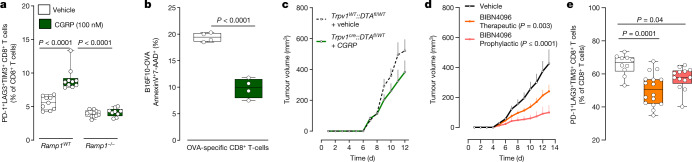
CGRP modulates the activation of CD8^+^ T cells. **a**,**b**, Splenocyte CD8^+^ T cells from wild-type (**a**), *Ramp1*^−/^^−^ (**a**) or naive OT-I (**b**) mice were cultured under T_c1_-stimulating conditions (ex-vivo-activated by CD3 and CD28, IL-12 and anti-IL4) for 48 h to generate cytotoxic CD8^+^ T cells. In the presence of IL-2 (10 ng ml^−1^), the cells were stimulated with CGRP (100 nM; challenged once every two days) for 96 h. Wild-type cytotoxic CD8^+^ T cells showed an increased proportion of PD-1^+^LAG3^+^TIM3^+^ cells; this effect was absent when treating cytotoxic CD8^+^ T cells that were collected from *Ramp1*^−/^^−^ mice (**a**). In co-culture (48 h), CGRP (100 nM; once daily) also reduced the ability of OT-I cytotoxic CD8^+^ T cells (4 × 10^5^ cells) to eliminate B16F10-mCherry-OVA cancer cells (**b**). **c**, Orthotopic B16F10-mCherry-OVA cells (5 × 10^5^ cells, i.d.) were inoculated into eight-week-old female mice with sensory neurons intact or ablated. In nociceptor-ablated mice, peritumoral recombinant CGRP injection (100 nM, i.d., once daily) rescues B16F10 growth (assessed until day 12). **d**,**e**, Orthotopic B16F10-mCherry-OVA cells (5 × 10^5^ cells, i.d.) were inoculated into eight-week-old male and female mice. Starting one day after inoculation (defined as prophylactic), the RAMP1 antagonist BIBN4096 (5 mg kg^−1^) was administered systemically (i.p.) once every two days. In another group of mice, BIBN4096 (5 mg kg^−1^, i.p., every two days) injections were started once the tumour reached a volume of around 200 mm^3^ (defined as therapeutic). Prophylactic or therapeutic BIBN4096 treatments decreased tumour growth (**d**) and reduced the proportion of intratumoral PD-1^+^LAG3^+^TIM3^+^ CD8^+^ T cells (**e**; assessed until day 13). Data are shown as box-and-whisker plots (as defined in Fig. 1b, c), for which individual data points are given (**a**,**b**,**e**), or as mean ± s.e.m. (**c**,**d**). *n* as follows: **a**: *Ramp1*^*WT*^ CD8 + vehicle (*n* = 9), *Ramp1*^*WT*^ CD8 + CGRP (*n* = 10), *Ramp1*^−/^^−^ CD8 + vehicle (*n* = 10), *Ramp1*^−/^^−^ CD8 + CGRP (*n* = 9); **b**: *n* = 4 per group; **c**: intact + vehicle (*n* = 15), ablated + CGRP (*n* = 11); **d**: vehicle (*n* = 13), BIBN prophylactic (*n* = 16), BIBN therapeutic (*n* = 18); **e**: vehicle (*n* = 10), BIBN prophylactic (*n* = 13), BIBN therapeutic (*n* = 16). Experiments were independently repeated three times with similar results. *P* values were determined by one-way ANOVA with post-hoc Bonferroni (**a**,**e**), two-sided unpaired Student’s *t*-test (**b**) or two-way ANOVA with post-hoc Bonferroni (**c**,**d**). Source Data

**Extended Data Fig. 9:**
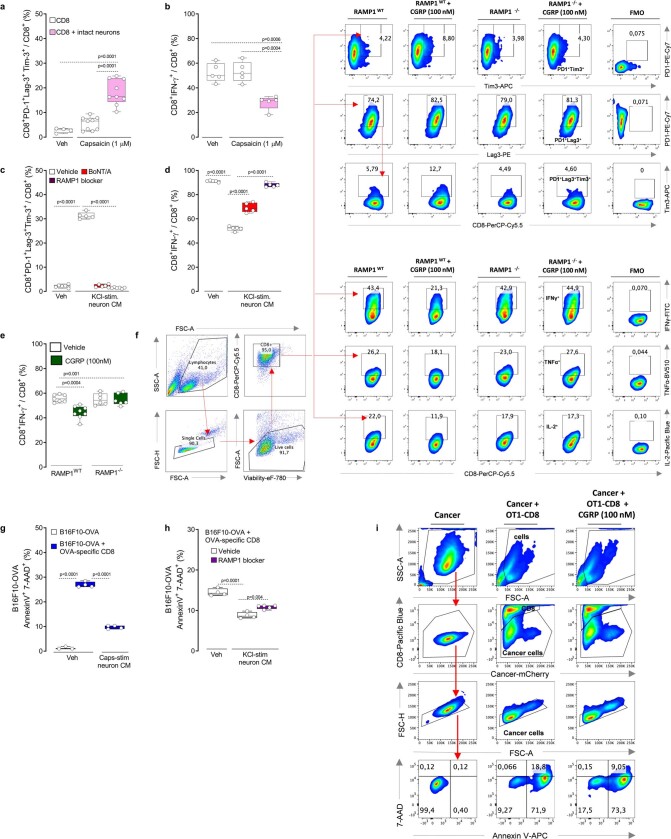
Nociceptor-released CGRP increases cytotoxic CD8^+^ T cell exhaustion. (**a–b**) Splenocytes-isolated CD8^+^ T cells were cultured under T_c1_-stimulating condition (*ex vivo* activated by CD3 and CD28, IL-12, and anti-IL4) for 48h. The cells were then cultured or not with wild-type DRG neurons and exposed to capsaicin (1 μM, challenged once every two days) or its vehicle. As measured after 4 days stimulation, capsaicin-stimulated intact neuron increased the proportion of PD-1^+^LAG3^+^TIM3^+^ (**a**) cytotoxic CD8^+^ T cells, while it decreased the one of IFNγ^+^ (**b**). (**c–d**) Splenocytes-isolated CD8^+^ T cells were cultured under T_c1_-stimulating conditions (*ex vivo* activated by CD3 and CD28, IL-12, and anti-IL4) for 48h. In the presence of peptidase inhibitors (1 μL/mL), naive DRG neurons were cultured in the presence of BoNT/A (50 pg/mL) or its vehicle for 24h. The cells were then washed, stimulated (30 min) with KCl (50mM), and the conditioned medium collected. On alternate days for 4 days, the cytotoxic CD8^+^ T cells were exposed or not to a RAMP1 blocker (CGRP_8–37_; 2  μg/mL) and challenge (1:2 dilution) with fresh KCl-induced conditioned medium from naive, or BoNT/A-silenced neurons. As measured after 4 days stimulation, KCl-stimulated neuron-conditioned medium increased the proportion of PD-1^+^LAG3^+^TIM3^+^ (**c**) cytotoxic CD8^+^ T cells, while it decreased the one of IFNγ^+^ (**d**). Such effect was absent when cytotoxic CD8^+^ T cells were co-exposed to the RAMP1 blocker CGRP_8–37_ or challenged with the neuron conditioned medium collected from BoNT/A-silenced neurons (**c–d**). (**e–f**) Splenocytes-isolated CD8^+^ T cells from wild-type and *Ramp1*^−/^^−^ mice were cultured under T_c1_-stimulating conditions (*ex vivo* activated by CD3 and CD28, IL-12, and anti-IL4) for 48h. On alternate days for 4 days, the cytotoxic CD8^+^ T cells were exposed to CGRP (0.1 μM) or its vehicle. As measured after 4 days stimulation, representative flow cytometry plots (**f**) show that CGRP decrease *Ramp*^*WT*^ cytotoxic CD8^+^ T cells expression of IFNγ^+^ (**e**,**f**), TNF^+^ (**f**), and IL-2^+^ (**f**) when exposed to CGRP. Inversely, CGRP increase the proportion of PD-1^+^LAG3^+^TIM3^+^ in *Ramp1*^*W*T^ cytotoxic CD8^+^ T cells (**f**). *Ramp1*^−/^^−^ cytotoxic CD8^+^ T cells were protected from the effect of CGRP (**e–f**). (**g–i**) Splenocytes-isolated CD8^+^ T cells from naive OT-I mice were cultured under Tc1-stimulating conditions (*ex vivo* activated by CD3 and CD28, IL-12, and anti-IL4) for 48h. B16F10-mCherry-OVA cells (1×10^5^ cells) were then cultured with or without OT-I cytotoxic CD8^+^ T cells (4×10^5^ cells). Tc1-stimulated OT-I-CD8^+^ T cells lead to B16F10-OVA cell apoptosis (AnnexinV^+^7AAD^+^; **g**, measured after 48h; **h–i**, measured after 24h). B16F10-mCherry-OVA cells elimination by cytotoxic CD8^+^ T cells was reduced when the co-cultures were challenged (1:2 dilution; once daily for two consecutive days) with fresh conditioned medium collected from capsaicin (1 μM)-stimulated naive DRG neurons (**g**; measured after 48h). Similarly, KCl (50mM)-stimulated naive DRG neurons conditioned medium (1:2 dilution) reduced B16F10-mCherry-OVA apoptosis (**h**; measured after 24h). This effect was blunted when the cells were co-exposed to the RAMP1 blocker CGRP_8-37_ (**h**; 2 μg/mL; measured after 24h). CGRP (0.1 μM) challenges also reduced OT-I cytotoxic CD8^+^ T cells elimination of B16F10-OVA cell (**i**; measured after 24h). Data are shown as box-and-whisker plots (runs from minimal to maximal values; the box extends from 25^th^ to 75^th^ percentile and the middle line indicates the median), for which individual data points are given (**a–e**, **g–h**), or representative FACS plot (**f**, **i**). N are as follows: **a**: CD8 + vehicle (n = 4), CD8 + capsaicin (n = 9), CD8 + neuron + capsaicin (n = 9), **b**: n = 5/groups, **c**: CD8 (n = 6), CD8 + KCl-induced neurons CM (n = 5), CD8 + KCl-induced neurons CM + CGRP_8-37_ (n = 6), CD8 + KCl-induced neurons CM + BoNT/A (n = 6), **d**: n = 5/groups, **e**: *Ramp1*^*WT*^ CD8 + vehicle (n = 7), *Ramp1*^*WT*^ CD8 + CGRP (n = 8), *Ramp1*^−/^^−^ CD8 + vehicle (n = 6), *Ramp1*^−/^^−^ CD8 + CGRP (n = 6), **g**: B16F10 (n = 3), B16F10 + OT-I CD8 (n = 4), B16F10 + OT-I CD8 + KCl-induced neuron CM (n = 4), **h**: B16F10 + OT-I CD8 (n = 4), B16F10 + OT-I CD8 + KCl-induced neuron CM (n = 4), B16F10 + OT-I CD8 + KCl-induced neuron CM + CGRP_8–37_ (n = 5). Experiments were repeated a minimum of three independent times with similar results. P-values are shown in the figure and determined by one-way ANOVA posthoc Bonferroni (**a–e**, **g–h**). Source Data

In breast cancer, tumour-specific sympathetic denervation downregulates the expression of PD-L1, PD-1 and FOXP3 in TILs^15^. Human and mouse cytotoxic CD8^+^ T cells express multiple neuropeptide receptors (10 or more), including the CGRP receptor RAMP1 (Extended Data Figs. 1b and 3b). Given that nociceptors readily interact with CD8^+^ T cells in culture and that the neuropeptides they release block anti-bacterial immunity^33,35–37^, we aimed to test whether these mediators drive the expression of immune checkpoint receptors in CD8^+^ T cells. First, splenocyte-isolated CD8^+^ T cells were cultured under type 1 (T_c1_) CD8^+^ T cell-stimulating conditions for two days and then co-cultured with DRG neurons for an additional four days. We found that nociceptor stimulation with capsaicin increased the proportion of PD-1^+^LAG3^+^TIM3^+^-expressing CD8^+^ T cells but decreased the levels of IFNγ^+^, TNF^+^ and IL-2^+^. Capsaicin had no measurable effect on CD8^+^ T cells in the absence of DRG neurons (Extended Data Fig. 9a,b). When T_c1_-activated CD8^+^ T cells were exposed to fresh conditioned medium (1:2 dilution) collected from KCl (50 mM)-stimulated DRG neurons, this treatment increased the proportion of PD-1^+^LAG3^+^TIM3^+^ cytotoxic CD8^+^ T cells and reduced that of IFNγ^+^ cells (Extended Data Fig. 9c,d; measured after four days of co-culture). These effects were prevented when the cytotoxic CD8^+^ T cells were challenged (1:2 dilution) with fresh KCl-induced conditioned medium from BoNT/A-silenced neurons (50 pg per 200 µl) or when they were co-exposed to the RAMP1 blocker CGRP_8–37_ (2 µg ml^−1^; Extended Data Fig. 9c,d). To confirm that nociceptor-released neuropeptides drive T cell exhaustion, we exposed T_c1_-activated CD8^+^ T cells to CGRP. CGRP-treated cells expressing wild-type RAMP1 showed increased exhaustion and limited cytotoxic potential. These effects were absent in CGRP-exposed CD8^+^ T cells that were collected from CGRP-receptor-knockout (*Ramp1*^−/^^−^) mice (Fig. 4a and Extended Data Fig. 9e,f).

We then assessed whether neuropeptides released by nociceptor neurons blunt the anti-tumour responses of cytotoxic CD8^+^ T cells through exhaustion. OT-I cytotoxic T cells induced robust apoptosis of cultured B16F10-mCherry-OVA cells (AnnexinV^+^7AAD^+^ B16F10-mCherry-OVA; Extended Data Fig. 9g–i). However, this apoptosis of B16F10-mCherry-OVA cells was decreased when the T cells were exposed to capsaicin- or KCl-stimulated neuron-derived conditioned medium, or when the cells were stimulated with CGRP (Fig. 4b and Extended Data Fig. 9g–i). OT-I cytotoxic T cells did not eliminate cultured B16F10-mCherry-OVA when co-exposed to KCl-induced neuron-conditioned medium supplemented with the RAMP1 blocker CGRP_8–37_ (2 µg ml^−1^; Extended Data Fig. 9h). When taken together with previous evidence that CGRP limits the activity of CD8^+^ T cells^12,38^, our data suggest that, through the CGRP–RAMP1 axis, nociceptors lead to the functional exhaustion of CD8^+^ T cells, as defined by a simultaneous loss of expression of cytotoxic molecules (that is, IFNγ and TNF) and proliferative capacity (IL-2), increased co-expression of several exhaustion markers (PD-1^+^LAG3^+^TIM3^+^) and a reduced capacity to eliminate malignant cells.

**Extended Data Fig. 10:**
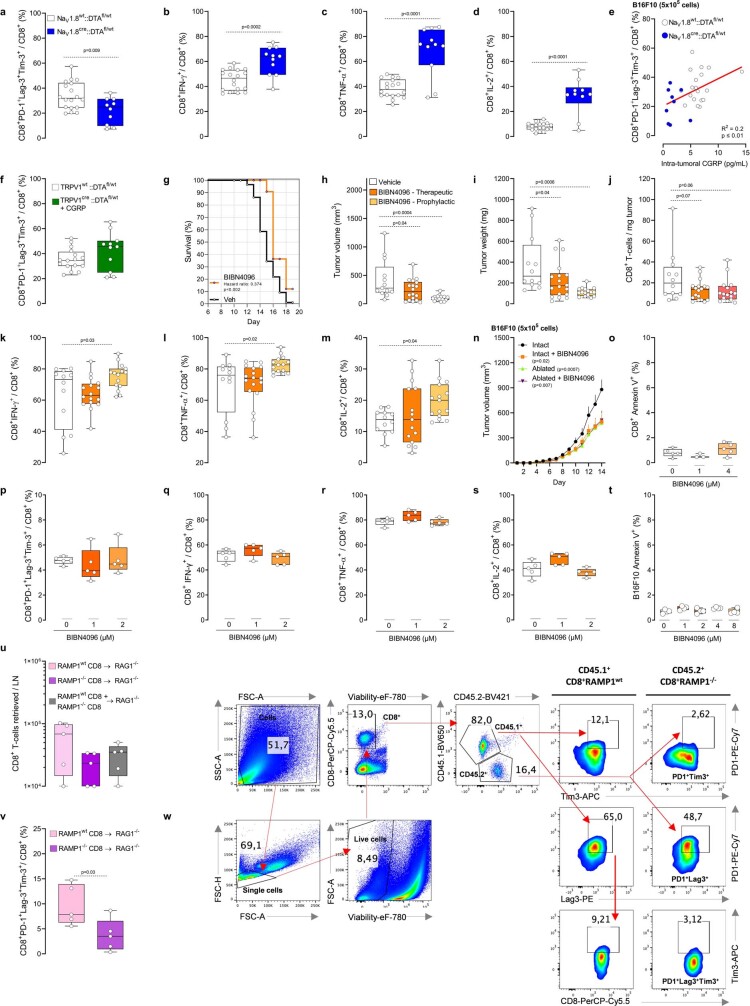
The CGRP–RAMP1 axis promotes intratumoral CD8^+^ T cell exhaustion. (**a–e**) Orthotopic B16F10-mCherry-OVA (5x10^5^ cells; i.d.) cells were injected to nociceptor intact (*Nav1.8*^*WT*^*::DTA*^*fl/WT*^) and ablated mice (*Nav1.8*^*cre*^*::DTA*^*fl/WT*^). As measured fifteen days post inoculation, Na_V_1.8^+^ nociceptor-ablated mice had lower proportion of PD-1^+^LAG3^+^TIM3^+^ (**a**) CD8^+^ T cells, but increased levels of IFNγ^+^ (**b**), TNF^+^ (**c**), IL-2^+^ (**d**) CD8^+^ T cells. B16F10-mCherry-OVA (5x10^5^ cells; i.d.)-tumour surrounding skin was also collected and capsaicin-induced CGRP release assessed by ELISA. Intratumoral CGRP levels positively correlate with the proportion of PD-1^+^LAG3^+^TIM3^+^ CD8^+^ T cells (**e**). (**f**) Orthotopic B16F10-mCherry-OVA cells (5x10^5^ cells; i.d.) were inoculated into 8-week-old female sensory neuron intact or ablated mice. In nociceptor-ablated mice, recombinant CGRP injection (100nM, i.d., once daily) rescues intratumoral CD8^+^ T cells exhaustion (PD-1^+^LAG3^+^TIM3^+^). (**g**) Orthotopic B16F10-mCherry-OVA cells (5x10^5^ cells; i.d.) were inoculated into 8-week-old male and female mice. Starting one day post inoculation, the RAMP1 antagonist BIBN4096 (5 mg/kg, i.p., every other day) was administered systemically. We found that blocking the action of CGRP on RAMP1-expressing cells, increased the mice’s median length of survival (~270% Mantel–Haenszel hazard ratio; measured on day 19). (**h–m**) Orthotopic B16F10-mCherry-OVA cells (5x10^5^ cells; i.d.) were inoculated into 8-week-old male and female mice. Starting one day post inoculation (*defined as prophylactic*), the RAMP1 antagonist BIBN4096 (5 mg/kg, i.p., every other day) was administered systemically. In another group of mice, BIBN4096 (5 mg/kg, i.p., every other day) injections were started once the tumour reached a volume of ~200mm^3^ (*defined as therapeutic*). The effect of nociceptor neuron-silencing on tumour size and tumour-infiltrating CD8^+^ T cell exhaustion was measured. As assessed thirteen days post tumour inoculation, BIBN4096 decreased tumour volume (**h**) and weight (**i**) but increased the relative proportion of IFNγ^+^ (**k**), TNF^+^ (**l**), and IL-2^+^ (**m**) CD8^+^ T cells. BIBN4096 had no effect on the number of intratumoral CD8^+^ T cells (**j**). When administered as therapeutic, BIBN4096 reduced tumour volume (**h**) and weight (**i**) but had limited effect on CD8^+^ T cells’ cytotoxicity (**j–m**). (**n**) Orthotopic B16F10-mCherry-OVA cells (5x10^5^ cells; i.d.) were inoculated into 8-week-old male and female sensory neuron-intact (*Trpv1*^*WT*^*::DTA*^*fl/WT*^) and ablated (*Trpv1*^*cre*^*::DTA*^*fl/WT*^) mice. Starting one day post inoculation, BIBN4096 (5 mg/kg) or its vehicle was administered (i.p.) on alternate days; effects on tumour volume were measured. Fourteen days post tumour inoculation, we found that tumour growth was reduced in sensory neuron-ablated mice and in BIBN4096-treated mice. BIBN4096 had no additive effect when given to sensory neuron-ablated mice. (**o–s**) Splenocytes-isolated CD8^+^ T cells from naïve C57BL6J mice were cultured under Tc1-stimulating conditions (*ex vivo* activated by CD3 and CD28, IL-12, and anti-IL4) for 48h. The cells were then exposed to BIBN4096 (1–4 μM) for 24h; effects on apoptosis, exhaustion and activation were measured by flow cytometry. When compared to vehicle-exposed cells, BIBN4096 did not affect the survival (**o**) of cultured cytotoxic CD8^+^ T cells, nor their relative expression of PD-1^+^LAG3^+^TIM3^+^ (**p**), IFNγ^+^ (**q**), TNF^+^ (**r**), and IL-2^+^ (**s**). (**t**) B16F10 cells (1x10^5^ cells) were cultured for 24h. The cells were then exposed (or not) to BIBN4096 (1-8 μM) for an additional 24h; effects on apoptosis were measured by flow cytometry. BIBN4096 did not trigger B16F10 cells apoptosis, as measured by the mean fluorescence intensity of Annexin V. (**u-w**) Naive splenocyte CD8^+^ T cells were FACS purified from *Ramp1*^*WT*^ (CD45.1^+^) or *Ramp1*^−/^^−^ (CD45.2^+^) mice, expanded and stimulated (*CD3 and CD28 + IL-2*) *in vitro*. 8-week-old female *Rag1*^−/^^−^ mice were transplanted (i.v., 2.5x10^6^ cells) with either *Ramp1*^−/^^−^ or *Ramp1*^*WT*^ CD8^+^ T cells or 1:1 mix of *Ramp1*^−/^^−^ and *Ramp1*^*WT*^ CD8^+^ T cells. One week post transplantation, the mice were inoculated with B16F10-mCherry-OVA cells (5x10^5^ cells; i.d.). Ten days post tumour inoculation, we retrieved a similar number of tumours draining lymph node CD8^+^ T cells across the three tested groups (**u**). The relative proportion of intra-tumour PD-1^+^LAG3^+^TIM3^+^ CD8^+^ T cells was lower in *Ramp1*^−/^^−^ transplanted mice (**v**). Within the same tumour, intratumoral CD8^+^ T cell exhaustion was immunophenotyped by flow cytometry (*representative panel shown in****w***) and showed that the relative proportion of PD-1^+^LAG3^+^TIM3^+^ CD8^+^ T cells was ~3-fold lower in *Ramp1*^−/^^−^ CD8^+^ T cells than in *Ramp1*^*WT*^ CD8^+^ T cells (**w**). Data are shown as box-and-whisker plots (runs from minimal to maximal values; the box extends from 25^th^ to 75^th^ percentile and the middle line indicates the median), for which individual data points are given (**a–d**, **f**, **h–m**, **o–v**), linear regression (**e**), Mantel–Cox regression (**g**), mean ± S.E.M (**n**), or as FACS plot (**w**). N are as follows **a–e**: *Nav1.8*^*WT*^*::DTA*^*fl/WT*^ (n = 18), *Nav1.8*^*cre*^*::DTA*^*fl/WT*^ (n = 10), **f**: *Trpv1*^*WT*^*::DTA*^*fl/WT*^ (n = 16), *Trpv1*^*cre*^*::DTA*^*fl/WT*^ +CGRP (n = 11), **g**: vehicle (n = 89), BIBN4096 (n = 16), **h–m**: Vehicle (n = 13), BIBN4096 therapeutic (n = 18), BIBN4096 prophylactic (n = 16), **n**: *Trpv1*^*WT*^*::DTA*^*fl/WT*^ + vehicle (n = 8), *Trpv1*^*WT*^*::DTA*^*fl/WT*^ + BIBN4096 (n = 9), *Trpv1*^*cre*^*::DTA*^*fl/WT*^ + vehicle (n = 7), *Trpv1*^*cre*^*::DTA*^*fl/WT*^ + BIBN4096 (n = 7), **o**: vehicle (n = 5), 1µM BIBN4096 (n = 3), 4 µM BIBN4096 (n = 5), **p–s**: n = 5/groups, **t**: n = 4/groups, **u–w**: n = 5/groups. Experiments were independently repeated twice (**a–f**, **n–w**) or four (**g–m**) times with similar results. P-values are shown in the figure and determined by two-sided unpaired Student’s t-test (**a–d**, **f**,**v**), simple linear regression analysis (**e**), Mantel–Cox regression (**g**), by one-way ANOVA posthoc Bonferroni (**h–m; o–u**), or two-way ANOVA post-hoc Bonferroni (**n**). Source Data

Nociceptor-produced neuropeptides reduce immunity against bacteria^37^ and fungi^39^, and promote cytotoxic CD8^+^ T cell exhaustion (Fig. 4a,b and Extended Data Fig. 9). Given that nociceptor-released CGRP is increased when cultured with B16F10 cells (Fig. 1c) or exposed to SLPI (Fig. 2f), and that tumour-infiltrating nociceptor neurons overexpress *Calca* (Fig. 1d,e), we next sought to test whether the intratumoral levels of CGRP correlate with CD8^+^ T cell exhaustion. To do this we used an *Nav1.8*^*cre*^ driver to ablate most mechano- and thermosensitive nociceptors with diphtheria toxin (*Nav1.8*^*cre*^*::DTA*^*fl/WT*^)^17,37^. When compared with melanoma-bearing littermate controls (*Nav1.8*^*WT*^*::DTA*^*fl/WT*^), the ablation of Na_V_1.8^+^ sensory neurons preserved the functionality of intratumoral CD8^+^ T cells (Extended Data Fig. 10a–d). In both groups of mice, the proportion of intratumoral CGRP directly correlated with the frequency of PD-1^+^LAG3^+^TIM3^+^ CD8^+^ T cells (Extended Data Fig. 10e).

We then set out to rescue CGRP levels (by daily intratumoral injection) in sensory-neuron-ablated mice and measured the effect on tumour growth and TIL exhaustion. At 11 days after inoculation, CGRP-treated sensory-neuron-ablated mice (*Trpv1*^*cre*^*::DTA*^*fl/WT*^) showed similar tumour growth and CD8^+^ T cell exhaustion to that of nociceptor-intact mice (Fig. 4c and Extended Data Fig. 10f). Next, we treated tumour-bearing mice with the selective RAMP1 antagonist BIBN4096 (5 mg kg^−1^, i.p., once every two days). The latter was previously found to block neuro–immune interactions during microorganism infections and rescues host anti-bacterial activity^35^. BIBN4096-exposed mice succumb at a rate 2.6-fold lower (*P* ≤ 0.02) than that of vehicle-exposed B16F10-bearing mice (Extended Data Fig. 10g; measured until day 19). As measured on day 13, BIBN4096 (5 mg kg^−1^, i.p., every other day) reduced B16F10 growth, tumour weight and frequency of PD-1^+^LAG3^+^TIM3^+^ CD8^+^ T cells (Fig. 4d-e and Extended Data Fig. 10h–m). As BIBN4096 showed no effect when administered to nociceptor-ablated mice and did not affect cultured B16F10 cells or CD8^+^ T cell function in vitro (Extended Data Fig. 10n–t), we conclude that the anti-tumour property of BIBN4096 relies on the presence of active nociceptor neurons.

**Fig. 5:**
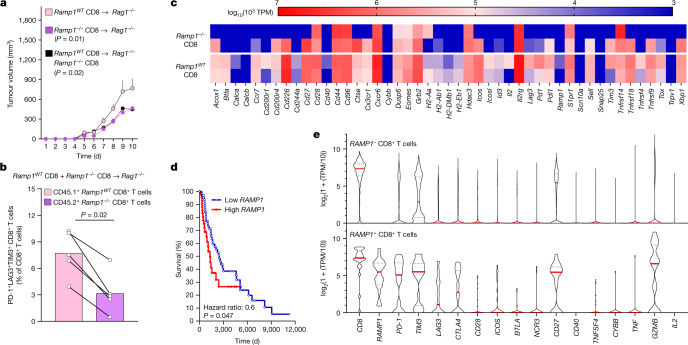
CGRP attenuates the anti-tumour immunity of RAMP1^+^ CD8^+^ T cells. **a**–**c**, Splenocyte CD8^+^ T cells were FACS purified from *Ramp1*^*WT*^ (CD45.1^+^) or *Ramp1*^−/^^−^ (CD45.2^+^) mice, expanded and stimulated (CD3 and CD28 + IL-2) in vitro. Eight-week-old female *Rag1*^−/^^−^ mice were transplanted (i.v., 2.5 × 10^6^ cells) with activated *Ramp1*^−/^^−^ or *Ramp1*^*WT*^ CD8^+^ T cells or a 1:1 mix of *Ramp1*^−/^^−^ and *Ramp1*^*WT*^ CD8^+^ T cells. One week after transplantation, the mice were inoculated with B16F10-mCherry-OVA cells (5 × 10^5^ cells, i.d.). Ten days after B16F10 inoculation, we observed greater tumour growth (**a**) in *Ramp1*^*WT*^ transplanted mice. Intratumoral *Ramp1*^−/^^−^ (CD45.2^+^) and *Ramp1*^*WT*^ (CD45.1^+^) CD8^+^ T cells were FACS purified, immunophenotyped (**b**) and RNA sequenced (**c**). *Ramp1*^−/^^−^ CD8^+^ T cells showed a lower proportion of PD-1^+^LAG3^+^TIM3^+^ CD8^+^ T cells (**b**) as well as reduced transcript expression of exhaustion markers (**c**). **d**, In silico analysis of The Cancer Genome Atlas (TCGA) data^40^ was used to correlate the survival rate of 459 patients with melanoma with the relative *RAMP1* expression (primary biopsy bulk RNA sequencing). In comparison to patients with low *RAMP1* expression, higher *RAMP1* levels correlate with decreased patient survival. **e**, In silico analysis of single-cell RNA sequencing of human melanoma^41^ reveals that intratumoral *RAMP1*-expressing CD8^+^ T cells strongly overexpress several immune checkpoint receptors (*PD-1* (also known as *PDCD1*) *TIM3*, *LAG3*, *CTLA4*) in comparison to *Ramp1*-negative CD8^+^ T cells. Data are shown as mean ± s.e.m. (**a**), slopegraph (**b**), as a heat map showing normalized gene expression (log_10_(10^3^ × TPM) (**c**), as a Mantel–Cox regression (**d**) or as a violin plot (**e**). *n* as follows: **a–c**: *n* = 5 per group; **d**: high (*n* = 45), low (*n* = 68); **e**: *RAMP1*^−^ CD8 (*n* = 1,732), *RAMP1*^+^ CD8 (*n* = 25). Experiments were independently repeated two (**a**,**b**) times with similar results. The sequencing experiment was not repeated (**c**). *P* values were determined by two-way ANOVA with post-hoc Bonferroni (**a**), two-sided unpaired Student’s *t*-test (**b**) or Mantel–Cox regression (**d**). Source Data

To directly address whether RAMP1 is the main driver of CD8^+^ T cell exhaustion, we transplanted *Rag1*^−/^^−^ mice with *Ramp1*^−/^^−^ or *Ramp1* wild-type (*Ramp1*^*WT*^) CD8^+^ T cells (intravenously (i.v.), 2.5 × 10^6^) or a 1:1 mixture of both. Although we retrieved similar numbers of CD8^+^ T cells across all three groups (Extended Data Fig. 10u), limited B16F10-OVA tumour growth (Fig. 5a) was found in mice that received the *Ramp1*^−/^^−^ CD8^+^ T cells—which are not responsive to CGRP. The relative proportion of intratumoral PD-1^+^LAG3^+^TIM3^+^ CD8^+^ T cells was also lower in *Ramp1*^−/^^−^-transplanted *Rag1*^−/^^−^ mice (Extended Data Fig. 10v). In *Rag1*^−/^^−^ mice co-transplanted with RAMP1-expressing and -non-expressing CD8^+^ T cells, we found that within the same tumour, the relative proportion of intratumoral PD-1^+^LAG3^+^TIM3^+^ CD8^+^ T cells was lower in *Ramp1*^−/^^−^ CD8^+^ T cells (Fig 5b and Extended Data Fig. 10w). Next, we RNA sequenced FACS-purified *Ramp1*^WT^ and *Ramp1*^−/^^−^ CD8^+^ T cells from these tumours. Compared to their *Ramp1*^*WT*^ counterparts, we found that intratumoral *Ramp1*^−/^^−^ CD8^+^ T cells expressed fewer pro-exhaustion transcription factors (*Tox* and *Eomes*) and markers (*Pdcd1* (encoding PD-1), *Lag3* and *Tim3* (also known as *Havcr2*); Fig. 5c). Overall, CGRP-unresponsive *Ramp1*^−/^^−^ CD8^+^ T cells are protected against undergoing nociceptor-induced exhaustion, which safeguards their anti-tumour responses.

**Extended Data Fig. 11:**
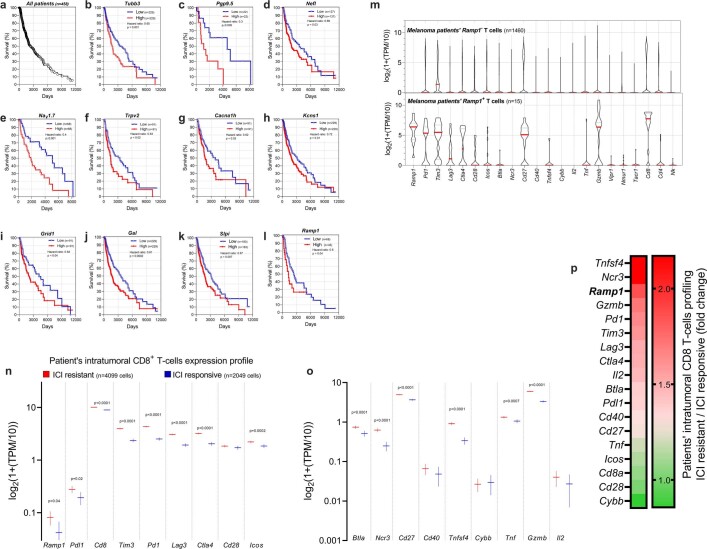
*RAMP1* expression in patient melanoma-infiltrating T cells correlates with worsened survival and poor responsiveness to ICIs. (**a–l**) In silico analysis of Cancer Genome Atlas (TCGA) data linked the survival rate among 459 patients with melanoma with their relative expression levels of various genes of interest (determined by bulk RNA sequencing of tumour biopsy). Kaplan–Meier curves show the patients’ survival after segregation in two groups defined by their low or high expression of a gene of interest. Increased gene expression (labelled as high; red curve) of *TUBB3* (**b**), *PGP9.5* (**c**), *Nav1.7* (**E**), *SLPI* (**k**) and *RAMP1* (**l**) in biopsy correlate with decreased patient survival (p≤0.05). The mantel–Haenszel hazard ratio and number of patients included in each analysis are shown in the figure (**a–l**). Experimental details were defined in Cancer Genome Atlas (TCGA)^40^. (**m**) In silico analysis of single-cell RNA sequencing of human melanoma-infiltrating T cells revealed that *RAMP1*^+^ T cells downregulated *Il-2* expression and strongly overexpressed several immune checkpoint receptors (*PD-1*, *TIM3*, *LAG3*, *CTLA4*, *CD28*, *ICOS*, *BTLA*, *CD27*) in comparison to *RAMP1*^-^ T cells. Individual cell data are shown as a log_2_ of 1 + (transcript per million / 10). Experimental details and cell clustering were defined in Tirosh et al^42^. N are defined in each panel. (**n–p**) On the basis of the clinical response of patients with melanoma to immune checkpoint blocker, patients were clustered into two groups defined as ICI-responsive or ICI-resistant^41^. *In silico* analysis of single-cell RNA sequencing of patients’ biopsies revealed that tumour-infiltrating CD8^+^ T cells from patients who were resistant to ICIs significantly overexpressed *RAMP1* (2.0-fold), *PD-1* (1.7-fold), *LAG3* (1.6-fold), *CTLA4* (1.6-fold), and *TIM3* (1.7-fold; **n–p**). Individual cell data are shown as a log_2_(1+(transcript per million/10). Experimental details and cell clustering were defined in Jerby-Arnon et al^41^. P-values are shown in the figure and determined by two-sided unpaired Student’s t-test. N are defined in each panel (**n–o**). Source Data

When compared with benign nevi, patient melanomas showed increased expression of *Calca* (Extended Data Fig. 1d). Along with other markers of nociceptor neurons, overexpression of *RAMP1* in these biopsies^40^ correlates (*P* ≤ 0.05) with reduced patient survival (Fig. 5d and Extended Data Fig. 11a–l). Whether RAMP1 does this by affecting intratumoral CD8^+^ T cell exhaustion is unknown. To answer this, we analysed two independent unbiased single-cell RNA-sequencing datasets of human melanomas^41,42^, and found that around 1.5% of tumour-infiltrating CD8^+^ T cells expressed *RAMP1*. The melanoma-infiltrating *RAMP1*^+^ CD8^+^ T cells of the patients overexpressed the immune checkpoint receptors *PD-1* (also known as *PDCD1*), *TIM3* (*HAVCR2*), *LAG3*, *CTLA4* and *CD27* (Fig. 5e and Extended Data Fig. 11m). This analysis also revealed that tumour-infiltrating CD8^+^ cells collected from patients who were resistant to ICIs markedly overexpressed *RAMP1* (Extended Data Fig. 11n–p). Such an expression profile resembles the functional exhaustion of effector CD8^+^ T cells and suggests that the CGRP receptor RAMP1 influences CD8^+^ T cell exhaustion and the clinical response to ICI in patients with melanoma.

Overall, the genetic ablation of nociceptor neurons decreases the growth of B16F10 tumours by preventing CD8^+^ T cells from undergoing exhaustion, whereas exogenous administration of CGRP has the opposite effect. These effects are restricted to immunogenic tumours and are not present in the absence of CD8 T cells. Similar to the pre-clinical modelling in mice, human data imply that RAMP1-expressing CD8^+^ T cells are more prone to exhaustion and are associated with lower responsiveness to ICIs.

**Extended Data Fig. 12:**
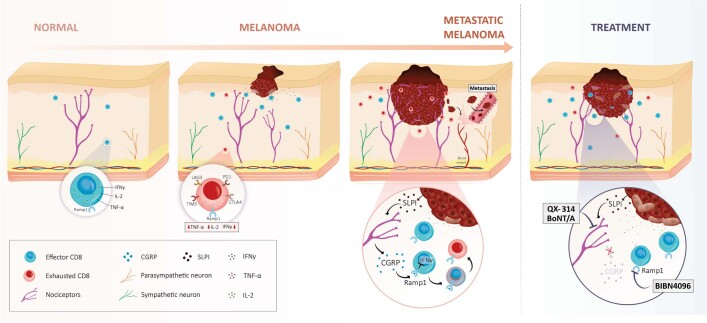
Melanoma-innervating nociceptors attenuate cancer immunosurveillance. Melanoma growth sets off anti-tumour immune responses, including the infiltration of effector CD8 T cells and their subsequent release of cytotoxic cytokines (i.e., IFNγ, TNF, Granzyme B). By acting on tissue-resident nociceptor neurons, melanoma-produced SLPI promotes pain hypersensitivity, tweaks the neurons’ transcriptome, and drives neurite outgrowth. These effects culminate in dense melanoma innervation by nociceptors and abundant release of immunomodulatory neuropeptides. CGRP, one such peptide, acts on tumour-infiltrating effector CD8^+^ T cells that express the CGRP receptor RAMP1, increasing their expression of immune checkpoint receptors (i.e., PD-1, LAG3, TIM3). Therefore, along with the immunosuppressive environment present in the tumour, nociceptor-produced CGRP leads to the functional exhaustion of tumour-infiltrating CD8^+^ T cells, which opens the door to unchecked proliferation of melanoma cells. Genetically ablating (i.e., TRPV1 lineage) or pharmacologically silencing (i.e., QX-314, BoNT/A) nociceptor neurons as well as blocking the action of CGRP on RAMP1 using a selective antagonist (i.e., BIBN4096) prevents effector CD8^+^ T cells from undergoing exhaustion. Therefore, targeting melanoma-innervating nociceptor neurons constitutes a novel strategy to safeguard host anti-tumour immunity and stop tumour growth.

Tumour-innervating nociceptors dampen the immune response to melanoma by upregulating multiple immune checkpoint receptors on cytotoxic CD8^+^ T cells. Blocking the CGRP–RAMP1 axis attenuates this immunomodulatory action of the nervous system on CD8^+^ T cells, thereby safeguarding the anti-tumour immunity of the host (Extended Data Fig. 12) and providing potential therapeutic opportunities by interrupting pro-cancerous neuro–immune links.

## Methods

### Secondary use of biopsies as research specimens

The ten melanoma samples used in this study were collected by Sanford Health and classified by a board-certified pathologist. Their secondary use as research specimens (fully de-identified formalin-fixed, paraffin-embedded (FFPE) blocks) was approved under Sanford Health IRB protocol 640 (titled ‘Understanding and improving cancer treatment of solid tumours’). As part of this Institutional Review Board (IRB)-approved retrospective tissue analysis, and in accordance with the US Department of Health and Human Services (HHS) secretary’s advisory committee on human research protections, no patient consent was necessary as these secondary use specimens were free of linkers or identifiers and posed no more than minimal risk to the human individuals.

### Immunohistochemistry and scoring

In compliance with all the relevant ethical regulations and as approved by Sanford Health IRB protocol 640, ten fully de-identified FFPE melanoma blocks were randomly selected for secondary use as research specimens. The notes of a board-certified pathologist on these specimens are provided in Supplementary Table 1. The specimens were stained using a BenchMark XT slide staining system (Ventana Medical Systems). The Ventana iView DAB detection kit was used as the chromogen, and slides were counterstained with haematoxylin and anti-TRPV1 (Alomone Labs, ACC-030; 1:100). Haematoxylin and eosin (H&E) staining followed standard procedures. TRPV1 immunohistochemistry-stained specimens were analysed on an Olympus BX51 bright-field microscope. Sections were viewed under 20× magnification. Five random fields per sample for both tumour and adjacent normal tissue were analysed and scored on a scale from 0 to 3. Scores were averaged. A score of 0 indicates no appreciated nerve fibres in the evaluated field; +1 indicates sparse nerve fibres; +2 indicates 5–20 nerve fibres; +3 indicates more than 20 nerve fibres.

### IACUC approval

The Institutional Animal Care and Use Committee (IACUC) of Boston Children’s Hospital and of the Université de Montréal (Comité de Déontologie de l’Expérimentation sur les Animaux; #21046; 21047) approved all animal procedures.

### Housing of mice

Mice were housed in standard environmental conditions (12-h light–dark cycle; 23 °C; food and water ad libitum) at facilities accredited by the Canadian Council of Animal Care (Université de Montréal) or the Association for Assessment and Accreditation of Laboratory Animal Care (Boston Children’s Hospital).

### IACUC end-points

As per our IACUC-approved protocol, the following end-points were used in all of the experiments and were not exceeded. Along with excessive body weight loss (maximum of 10%), the end-points include excessive tumour volume (10% of the mouse’s body weight; around 17 mm × 17 mm), skin ulceration, necrosis, bleeding, infection and self-inflicted injury, prostration, lethargy, unresponsiveness to stimulation and/or lack of grooming.

### Mouse lines

Six-to-twenty-week-old male and female C57BL6J (Jax, 000664); CD45.1^+^ C57BL6J (Jax, 002014), *Ramp1*^−/^^−^ (Jax, 031560), *Rag1*^−/^^−^ (Jax, 002216), OT-I (Jax, 003831)^43^, *Trpv1*^*cre*^ (Jax, 017769)^44^, *ChR2*^*fl/fl*^ (Jax, 012567)^45^, *tdTomato*^*fl/fl*^ (Jax, 007908)^46^, *DTA*^*fl/fl*^ (Jax, 009669)^47^ or *DTA*^*fl/fl*^ (Jax, 010527), *QuasAr2-dark mOrange2-CheRiff-eGFP*^*fl/fl*^ (referred to in the text as *CheRiff-eGFP*^*fl/fl*^; Jax, 028678)^48^ mice were purchased from the Jackson Laboratory. *Nav1.8*^*cre*^ mice^49^ were supplied by R. Kuner and J. Wood. Excluding CD45.1^+^ mice, all other lines were backcrossed for more than six generations on a C57BL6/J background (H-2Kb). Although Capecchi’s *DTA*^*fl/fl*^ (Jax, 010527) was created on a mixed C57BL6J/129 background, both haplotypes are H-2Kb. All these mice are therefore fully compatible with being transplanted with B16F10-derived cells (C57BL6/J background (H-2Kb)).

We used the Cre–*lox* toolbox to engineer the various mice lines used (*Trpv1*^*cre*^*::DTA*^*fl/WT*^, *Trpv1*^*cre*^*::CheRiff-eGFP*^*fl/WT*^, *Trpv1*^*cre*^*::tdTomato*^*fl/WT*^, *Nav1.8*^*cre*^*::DTA*^*fl/WT*^, *Nav1.8*^*cre*^*::tdTomato*^*fl/WT*^; *Nav1.8*^*cre*^*::ChR2*^*fl/WT*^ and littermate control) by crossing heterozygote Cre mice with homozygous *loxP* mice. Mice of both sexes were used for these crosses. All Cre driver lines used were viable and fertile, and abnormal phenotypes were not detected. Offspring were tail-clipped and tissue was used to assess the presence of the transgene by standard PCR, as described by The Jackson Laboratory or the donating investigators. Offspring of both sexes were used at 6–20 weeks of age.

### Cell lines

B16F0^50^ (ATCC, CRL-6322), B16F10^51^ (ATCC, CRL-6475), B16F10-mCherry-OVA^52^ (M. F. Krummel, UCSF), B16F10-eGFP (Imanis, CL053), YUMM1.7^29^ (ATCC, CRL-3362), and non-tumorigenic keratinocytes (CellnTEC, MPEK-BL6100) were cultured in complete Dulbecco’s modified Eagle’s medium high glucose (DMEM; Corning, 10-013-CV) supplemented with 10% fetal bovine serum (FBS; Seradigm, 3100) and 1% penicillin–streptomycin (Corning, MT-3001-Cl), and maintained at 37 °C in a humidified incubator with 5% CO_2_. YUMMER1.7^28^ (M. Bosenberg, Yale University) cells were cultured in DMEM F12 (Gibco, 11320033) supplemented with 10% FBS, 1% penicillin–streptomycin (Corning, MT-3001-Cl) and MEM nonessential amino acids (Corning, 25-025CI), and maintained at 37 °C in a humidified incubator with 5% CO_2_.

All the cell lines tested negative for mycoplasma, and none are listed by the International Cell Line Authentication Committee registry (v.11). Non-commercial cell lines (B16F10-OVA, B16F10-OVA-mCherry and B16F10-eGFP) were authenticated using antibodies (against OVA, eGFP and mCherry) and/or imaging as well as morphology and growth properties. Commercial cell lines were not further authenticated.

### Cancer inoculation and volume measurement

Cancer cells were resuspended in phosphate buffered saline (PBS; Corning, 21040CV) and injected into the mouse’s skin in the right flank (5 × 10^5^ cells, i.d., 100 μl) or hindpaw (2 × 10^5^ cells, i.d., 50 μl). Growth was assessed daily using a handheld digital caliper and tumour volume was determined by the formula (*L* × *W*^2^ × 0.52) (ref. ^53^), in which *L* = length and *W* = width.

### BoNT/A

BoNT/A^35^ (List Biological Labs, 130B) was injected (25 pg μl^−1^, i.d., five neighbouring sites injected with 20 µl) into the skin three days and one day before tumour inoculation (defined as prophylactic). BoNT/A (25 pg μl^−1^; i.d., five neighbouring sites injected with 20 µl) was injected around the tumour one day and three days after the tumour reached a volume of around 200 mm^3^ (defined as therapeutic) in other groups of C57BL/6J mice.

### QX-314

Starting one day after tumour inoculation (defined as prophylactic), QX-314 (ref. ^34^; Tocris, 2313; 0.3%) was injected (i.d., 100 µl) daily at five points around the tumour. In another group of mice, QX-314 daily injection started once the tumour reached a volume of around 200 mm^3^ (defined as therapeutic).

### BIBN4096

Starting one day after tumour inoculation, BIBN4096 (ref. ^33^; Tocris, 4561; 5 mg kg^−1^) was administered systemically (i.p., 50 µl) on alternate days to eight-week-old male and female mice (defined as prophylactic). In another group of mice, BIBN4096 (5 mg kg^−1^) was administered systemically (i.p., 50 µl) on alternate days once the tumour reached a volume of around 200 mm^3^ (defined as therapeutic).

### RTX

RTX (ref. ^33^; Alomone Labs, R-400) was injected (subcutaneously; s.c.) in three dosages (30, 70 and 100 μg kg^−1^) into the right flank of *Rag1*^−/^^−^ and C57BL/6J mice of around three weeks of age. Denervation was confirmed 28 days after RTX by an absence of pain withdrawal reflex (paw flinching) when exposed to heat (see ‘Thermal hypersensitivity’ for details on the test).

### Survival

In specific groups of mice, orthotopic B16F10-mCherry-OVA (5 × 10^5^ cells, i.d.) cells were administered to intact and nociceptor-ablated mice and survival was measured until day 22 and determined by the tumour reaching a volume of 1,000 mm^3^ or greater, or according to the ethical end-points described above. In B16F10-mCherry-OVA-inoculated mice treated with QX-314 or BIBN4096, survival was measured until day 19 and determined by the tumour reaching a volume of 800 mm^3^ or greater, or according to the ethical end-points described above. As the survival analysis of vehicle-injected, QX-314-treated and BIBN4096-treated mice was performed simultaneously, the same group of vehicle-injected mice is shown in the respective panels for QX-314 and BIBN4096.

### iDISCO imaging

Whole-mount immunohistochemistry of tumours was performed using an iDISCO protocol^54,55^ with methanol pre-treatment optimized for tumours. In brief, adult mice (eight weeks old) were perfused with 25 ml of PBS (HyClone) and 25 ml of 4% paraformaldehyde (PFA; Sigma) sequentially at room temperature. Tumours were post-fixed with 4% PFA for 6 h at 4 °C. For methanol pre-treatment, fixed tumours were washed sequentially in 50% methanol (in PBS) for 1 h and 100% methanol for 1 h, and then bleached in 5% H_2_O_2_ in 20% DMSO and methanol overnight at 4 °C. Tumours were subsequently rehydrated in 100% methanol for 1 h twice, 20% DMSO and methanol for 1 h twice, 50% methanol in PBS for 1 h, PBS for 1 h twice and PBS and 0.2% Triton X-100 for 1 h twice at room temperature. Tumours were then left in PBS, 0.2% Triton X-100, 20% DMSO and 0.3 M glycine (Sigma) overnight at room temperature and blocked in PBS, 0.2% Triton X-100, 10% DMSO, 6% donkey serum (Jackson ImmunoResearch) and anti-CD16/CD32 (Fc block; Bio X Cell) overnight at room temperature. Tumours were subsequently washed in PBS, 0.2% Tween-20 and 10 mg ml^−1^ heparin (PTwH; Sigma) for 1 h twice at room temperature before incubation with antibody mix (GFP (Aves Labs) at 1:500, mCherry (OriGene) at 1:500, in PTwH, 5% DMSO, 3% donkey serum and Fc block 1:100 for four days at room temperature). Tumours were extensively washed in PTwH at least six times over the course of one day at room temperature. Tumours were further incubated with a secondary panel of species-specific anti-IgG (H+L) Alexa Fluor 488 or 546-conjugated antibodies (Invitrogen or Jackson ImmunoResearch), all at 1:500, in PTwH, 5% DMSO, 3% donkey serum and Fc block 1:100 for three more days at room temperature. Tumours were washed in the same way as after primary antibody incubation for one day. Immunolabelled tumours were then processed for clearing, which included sequential incubation with 50% methanol for 1 h at room temperature, 100% methanol for 1 h three times at room temperature, and a mixture of one part benzyl alcohol (Sigma):two parts benzyl benzoates (Sigma) overnight at 4 °C. For tdTomato and GFP immunolabelling, mCherry and GFP antibodies were preabsorbed against tumours from *tdTomato*^−^ mice overnight at room temperature before use. Cleared whole-mount tissues were imaged in BABB between two cover glasses using Olympus FV3000 confocal imaging system.

### Tumour and tumour-draining lymph node digestion

Mice were euthanized when the tumour reached a volume of 800–1,500 mm^3^ (refs. ^50,51,56^). Tumours and their draining lymph nodes were collected. Tumours were enzymatically digested in DMEM + 5% FBS (Seradigm, 3100) + 2 mg ml^−1^ collagenase D (Sigma, 11088866001) + 1 mg ml^−1^ collagenase IV (Sigma, C5138-1G) + 40 μg ml^−1^ DNAse I (Sigma, 10104159001) under constant shaking (40 min, 37 °C). The cell suspension was centrifuged at 400*g* for 5 min. The pellet was resuspended in 70% Percoll gradient (GE Healthcare), overlaid with 40% Percoll and centrifuged at 500*g* for 20 min at room temperature with acceleration and deceleration at 1. The cells were aspirated from the Percoll interface and passed through a 70-μm cell strainer. Tumour-draining lymph nodes were dissected in PBS + 5% FBS, mechanically dissociated using a plunger, strained (70 μm) and washed with PBS.

### Immunophenotyping

Single cells were resuspended in FACS buffer (PBS, 2% fetal calf serum and EDTA), and stained with ZombieAqua (15 min, room temperature; BioLegend, 423102) or a Viability Dye eFluor 780 (15 min, 4 °C; eBioscience, 65-0865-14). The cells were washed and Fc-blocked (0.5 mg ml^−1^, 15 min, 4 °C; BD Biosciences, 553141). Finally, the cells were stained (30 min, 4 °C) with one of anti-CD45–BV421 (1:100, BioLegend, 103134), anti-CD45.1–BV421 (1:100, BioLegend, 110732), anti-CD45.2–BV650 (1:100, BioLegend, 109836), anti-CD45-Alexa Fluor 700 (1:100, BioLegend, 103128), anti-CD11b-APC/Cy7 (1:100, BioLegend, 101226), anti-CD8-AF700 (1:100, BioLegend, 100730), anti-CD8–BV421 (1:100, BioLegend, 100753), anti-CD8–PerCP/Cyanine5.5 (1:100, BioLegend, 100734), anti-CD8–Pacific Blue (1:100, BioLegend, 100725), anti-CD4–PerCP/Cyanine5.5 (1:100, BioLegend, 100540), anti-CD4-FITC (1:100, BioLegend, 100406), anti-PD-1–PE-Cy7 (1:100, BioLegend, 109110), anti-LAG3–PE (1:100, BioLegend, 125208), anti-LAG3–PerCP/Cyanine5.5 (1:100, BioLegend, 125212) or anti-TIM3–APC (1:100, BioLegend, 119706), washed and analysed using a LSRFortessa or FACSCanto II (Becton Dickinson). Antigen-specific CD8^+^ T cells were stained with H-2Kb/OVA257-264 (15 min, 37 °C; NIH tetramer core facility), washed and stained with surface markers. Cytokine expression was analysed after in vitro stimulation (PMA–ionomycin; see ‘Intracellular cytokine staining’).

### Intracellular cytokine staining

Cells were stimulated (3 h) with phorbol-12-myristate 13-acetate (PMA; 50 ng ml^−1^, Sigma-Aldrich, P1585), ionomycin (1 μg ml^−1^, Sigma-Aldrich, I3909) and Golgi Stop (1:100, BD Biosciences, 554724). The cells were then fixed and permeabilized (1:100, BD Biosciences, 554714) and stained with anti-IFNγ–APC (1:100, BioLegend, 505810), anti-IFNγ–FITC (1:100, BioLegend, 505806), anti-TNF–BV510 (1:100, BioLegend, 506339), anti-TNF–BV5711 (1:100, BioLegend, 506349), anti-TNF–PE (1:100, BioLegend, 506306), anti-IL2–Pecy7 (1:100, BioLegend, 503832), anti-IL-2–Pacific Blue (1:100, BioLegend, 503820), anti-IL-2–BV510 (1:100, BioLegend, 503833), and analysed using a LSRFortessa or FACSCanto II (Becton Dickinson).

### In vivo depletion of CD3 or CD8

Anti-mouse CD3 (200 μg per mouse, Bio X Cell, BE0001-1) or anti-mouse CD8 (200 μg per mouse, Bio X Cell, BP0061) were injected (i.p.) three days before B16F10-mCherry-OVA inoculation (5 × 10^5^ cells; i.d.) and continued every three days. Blood samples were taken twice weekly to confirm depletion, and tumour growth was measured daily.

### In vivo CGRP rescue experiment

*Trpv1*-ablated mice were injected (i.d.) once daily with recombinant CGRP (100 nM) at five points around the tumour (treatment began once the tumour was visible), and tumour growth was measured daily by a handheld digital caliper. Mice were euthanized, and tumour-infiltrating CD8^+^ cell exhaustion was immunophenotyped by flow cytometry using an LSRFortessa or a FACSCanto II (Becton Dickinson).

### Anti-PD-L1 treatment

Orthotopic B16F10-mCherry-OVA cells (5 × 10^5^ cells, i.d.) were inoculated into eight-week-old male and female sensory-neuron-intact or -ablated mice. On days 7, 10, 13 and 16 after tumour inoculations, the mice were treated with anti-PD-L1^14,57^ (Bio X Cell, BE0101, 6 mg kg^−1^; i.p., 50 µl) or isotype control. Nineteen days after tumour inoculation, the effect of anti-PD-L1 on tumour growth was analysed and TIL exhaustion was immunophenotyped using an LSRFortessa or a FACSCanto II (Becton Dickinson).

### Anti-PD-L1 treatment in mice with similar tumour sizes

Orthotopic B16F10-mCherry-OVA cells (5 × 10^5^ cells; i.d.) were injected into a cohort of nociceptor neuron-ablated mice three days before nociceptor-intact mice were injected. Mice from each group with a similar tumour size (around 85 mm^3^) were selected and exposed to anti-PD-L1^14,57^ (Bio X Cell, BE0101, 6 mg kg^−1^, i.p., 50 µl) or isotype control once every three days for a total of nine days. The effect of anti-PD-L1 treatment on tumour growth was analysed until day 18.

One and three days before tumour inoculation, the skin of eight-week-old male and female mice was injected with BoNT/A (25 pg μl^−1^, i.d., five neighbouring sites injected with 20 µl) or vehicle. One day after the last injection, orthotopic B16F10-mCherry-OVA cells (5 × 10^5^ cells, i.d.) were inoculated into the area pre-exposed to BoNT/A. On days 7, 10, 13 and 16 after tumour inoculation, the mice were exposed to anti-PD-L1 (6 mg kg^−1^, i.p.) or isotype control. Eighteen days after tumour inoculation, we found that neuron silencing using BoNT/A potentiated anti-PD-L1-mediated tumour reduction.

Orthotopic B16F10-mCherry-OVA (5 × 10^5^ cells, i.d.) were injected into mice treated with QX-314 (0.3%, i.d.) two to three days before being given to vehicle-exposed mice. Mice from each group with a similar tumour size (around 100 mm^3^) were selected and exposed to anti-PD-L1^14^ (Bio X Cell, BE0101, 6 mg kg^−1^, i.p.) or isotype control once every three days for a total of nine days. Eighteen days after tumour inoculation, the effect of anti-PD-L1 on tumour growth was analysed, and TIL exhaustion was immunophenotyped using an LSRFortessa or a FACSCanto II (Becton Dickinson).

### Adoptive transfer of *Ramp1*^*WT*^ or *Ramp1*^−/^^−^ CD8 T cells

Total CD8^+^ T cells were isolated from the spleen of wild-type (CD45.1^+^) or *Ramp1*^−/^^−^ (CD45.2^+^) mice, expanded and stimulated *in vitro* using a mouse T cell Activation/Expansion Kit (Miltenyi Biotec. #130-093-627). CD8^+^ cells from *Ramp1*^−/^^−^ and *Ramp1*^*WT*^ were injected separately or 1:1 mix through tail vein of *Rag1*^−/^^−^ mice. One week after, the mice were inoculated with B16F10-mCherry-OVA cancer cells (5 × 10^5^ cells; i.d.), and tumour growth was measured daily using a handheld digital caliper. On day 10, tumours were collected and *Ramp1*^−/^^−^ (CD45.2^+^) and *Ramp1*^*WT*^ (CD45.1^+^) CD8^+^ T cells were immunophenotyped using a FACSCanto II (Becton Dickinson) or FACS purified using a FACSAria IIu cell sorter (Becton Dickinson).

### RNA sequencing of adoptive transferred *Ramp1*^*WT*^ or *Ramp1*^−/^^−^ CD8 T cells

For FACS-purified cells, *Ramp1*^−/^^−^ and *Ramp1*^*WT*^ CD8^+^ T cell RNA-sequencing libraries were constructed using KAPA Hyperprep RNA (1 × 75 bp) following the manufacturer’s instructions. Nextseq500 (0.5 Flowcell High Output; 200 M defragments; 75 cycles single-end read) sequencing was performed on site at the Institute for Research in Immunology and Cancer (IRIC) genomic centre. Sequences were trimmed for sequencing adapters and low-quality 3′ bases using Trimmomatic v.0.35 and aligned to the reference mouse genome version GRCm38 (gene annotation from Gencode v.M23, based on Ensembl 98) using STAR v.2.5.1b (ref. ^58^). Gene expression levels were obtained both as a read count directly from STAR and computed using RSEM to obtain normalized gene and transcript level expression, in TPM values, for these stranded RNA libraries. DESeq2 v.1.18.1 (ref. ^59^) was then used to normalize gene read counts. Individual cell data are shown as a log_10_ of (TPM × 1,000). These data have been deposited in the National Center for Biotechnology Information (NCBI)’s Gene Expression Omnibus (GEO)^60^ (GSE205863).

### Adoptive T cell transfer in mice treated with RTX

CD8^+^ T cells were isolated from OT-I mice spleens and magnet sorted (StemCell; 19858). Naive CD8^+^ T cells (CD8^+^CD44^low^CD62L^hi^) cells were then purified by FACS using an FACSAria IIu cell sorter (Becton Dickinson) and injected (1 × 10^6^ cells, i.v., tail vein) into vehicle- or RTX-exposed *Rag1*^−/^^−^ mice.

### Mechanical hypersensitivity

B16F10-mCherry-OVA (2 × 10^5^ cells, i.d.) or non-cancerous keratinocytes (MPEK-BL6; (2 × 10^5^ cells, i.d.) were inoculated intradermally in the left hindpaw of the mice. On alternate days, mechanical sensitivity was evaluated using von Frey filaments (Ugo Basile, 52-37450-275). To do so, the mice were placed in a test cage with a wire mesh floor and allowed to acclimatize (three consecutive days: 1 h per session). Von Frey filaments of increasing size (0.008–2 g) were applied to the plantar surface and the response rate was evaluated using the up-down test paradigm^61^.

### Thermal hypersensitivity

To measure thermal sensitivity, the mice were placed on a glass plate of a Hargreaves’s apparatus (Ugo Basile)^62^ and stimulated using radiant heat (infrared beam). The infrared beam intensity was set at 44 and calibrated to result in a withdrawal time of around 12 seconds in acclimatized wild-type mice. An automatic cut-off was set to 25 s to avoid tissue damage. The radiant heat source was applied to the dorsal surface of the hindpaw and latency was measured as the time for the mouse to lift, lick or withdraw the paw^62^.

Before any treatment, the mice were allowed to acclimatize in the apparatus (minimum of three consecutive days: 1 h per session) and three baseline measurements were taken on the following day. In some instances, B16F10-mCherry-OVA (2 × 10^5^ cells; i.d.) or non-cancerous keratinocytes (MPEK-BL6; (2 × 10^5^ cells; i.d.) were inoculated intradermally to the mouse’s left hindpaw and thermal pain hypersensitivity was measured on alternate days (10:00). In other instances, SLPI (1 µg per 20 µl) or saline (20 µl) were injected in the left and right hindpaw, respectively, and thermal hypersensitivity was measured in both hindpaws at 1, 3 and 6 h after treatment.

### Kinetics of pain and intratumoral CD8 T cell exhaustion

We implanted B16F10-mCherry-OVA (2 × 10^5^ cells, i.d.) in several groups of littermate control (*Trpv1*^*WT*^*::DTA*^*fl/WT*^; *n* = 96) and nociceptor-ablated (*Trpv1*^*cre*^*::DTA*^*fl/WT*^; *n* = 18) mice. We then evaluated the level of thermal hypersensitivity (daily), tumour size (handheld digital caliper), and intratumoral CD8^+^ T cell exhaustion (flow cytometry) at the time of euthanasia (days 1, 4, 7, 8, 12, 13, 14, 19 and 22). We processed these data by determining the percentage change of each data point to the maximal value obtained in the pain, CD8^+^ T cell exhaustion and tumour size datasets, and then presented these data as percentages of the maximum (100%).

### Optogenetic stimulation

Orthotopic B16F10-mCherry-OVA cells (5 × 10^5^ cells, i.d.) were inoculated into the left flank of eight-week-old transgenic male mice expressing the light-sensitive protein channelrhodopsin 2 under the control of the *Nav1.8* promoter (*Nav1.8*^*cre*^*::ChR2*^*fl/WT*^). Optogenetic stimulation (3.5 ms, 10 Hz, 478 nm, 60 mW, in a 0.39-NA fibre placed 5–10 mm from the skin, for 20 min) started either when the tumour was visible (around 20 mm^3^; 5 days after inoculation) or when it reached a volume of 200 mm^3^ (8 days after inoculation) and lasted up to 14 days after tumour inoculation. The control mice (*Nav1.8*^*cre*^*::ChR2*^*fl/WT*^) were tumour-injected but not light-stimulated. Groups of littermate control (*Nav1.8*^*WT*^*::ChR2*^*fl/WT*^) mice were light-stimulated and showed no response (not shown).

### CGRP release from skin explant

Tumour-surrounding skin was collected using 10-mm punch biopsies from nociceptor-intact (*Nav1.8*^*WT*^*::DTA*^*fl/WT*^), nociceptor-ablated (*Nav1.8*^*cre*^*::DTA*^*fl/WT*^), light-sensitive nociceptor (*Nav1.8*^*cre*^*::ChR2*^*fl/WT*^) or wild-type mice 3 h after exposure to vehicle (100 μl), QX-314 (0.3%, 100 μl) or BoNT/A (25 pg μl^−1^, 100 μl). The biopsies were transferred into 24-well plates and cultured in DMEM containing 1 μl ml^−1^ of protease inhibitor (Sigma, P1860) and capsaicin (1 μM, Sigma, M2028). After a 30-min incubation (37 °C), the supernatant was collected and the release of CGRP was analysed using a commercial enzyme-linked immunosorbent assay (ELISA)^35^ (Cayman Chemical, 589001).

### CGRP release triggered by SLPI

1 × 10^4^ naive DRG neurons were cultured for 24 h in complete DMEM (10% FBS, 1% penicillin–streptomycin, 1 μl ml^−1^ protease inhibitor) and subsequently stimulated (3 h) with vehicle or SLPI (0.1–5.0 ng ml^−1^). After stimulation, the supernatant was collected and CGRP levels were measured using a commercial ELISA kit (Cayman Chemical, 589001).

### Neuron culture

Mice were euthanized, and dorsal root ganglia were dissected out into DMEM medium (Corning, 10-013-CV), completed with 50 U ml^−1^ penicillin and 50 μg ml^−1^ streptomycin (Corning, MT-3001-Cl) and 10% FBS (Seradigm, 3100). Cells were then dissociated in HEPES buffered saline (Sigma, 51558) completed with 1 mg ml^−1^ collagenase IV (Sigma, C0130) + 2.4 U ml^−1^ dispase II (Sigma, 04942078001) and incubated for 80 min at 37 °C. Ganglia were triturated with glass Pasteur pipettes of decreasing size in supplemented DMEM medium, then centrifuged over a 10% BSA gradient and plated on laminin (Sigma, L2020)-coated cell-culture dishes. Cells were cultured with Neurobasal-A medium (Gibco, 21103-049) completed with 0.05 ng μl^−1^ NGF (Life Technologies, 13257-019), 0.002 ng μl^−1^ GDNF (PeproTech, 450-51-10), 0.01 mM AraC (Sigma, C6645) and 200 mM l-glutamine (VWR, 02-0131) and B-27 supplement (Gibco, #17504044).

### Calcium imaging

L3–L5 DRG neurons were collected and co-cultured with B16F10, B16F0 or MPEK-BL6 for 24–48 h. The cells were then loaded with 5 mM Fura-2 AM (BioVision, 2243) in complete Neurobasal-A medium for 30 min at 37 °C, washed in Standard Extracellular Solution (SES, 145 mM NaCl, 5 mM KCl, 2 mM CaCl_2_, 1 mM MgCl_2_, 10 mM glucose and 10 mM HEPES, pH 7.5), and the response to noxious ligands (100 nM capsaicin, 100 μM AITC or 1 μM ATP) was analysed at room temperature. Ligands were flowed (15 s) directly onto neurons using perfusion barrels followed by buffer washout (105-s minimum). Cells were illuminated by a UV light source (Xenon lamp, 75 watts, Nikon), 340-nm and 380-nm excitation alternated by an LEP MAC 5000 filter wheel (Spectra services), and fluorescence emission was captured by a Cool SNAP ES camera (Princeton Instruments). The 340/380 ratiometric images were processed, background-corrected and analysed (IPLab software, Scientific Analytics), and Microsoft Excel was used for post-hoc analyses. Responsiveness to a particular ligand was determined by an increase in fluorescence (*F*_340_/*F*_380_) of at least 5–10% above baseline recording (SES). To test neuronal sensitivity in mice inoculated with B16F10 or non-tumorigenic keratinocytes, the mice were euthanized two weeks after inoculation (left hindpaw, i.d.), and L3–L5 DRG neurons were collected and cultured (3 h). Calcium flux to noxious ligands (1 μM capsaicin or 10 μM ATP) was subsequently tested. For SLPI, the DRG neurons were cultured for 24 h, loaded with 5 mM Fura-2 AM in complete Neurobasal-A medium for 45 min at 37 °C and washed into SES, and the responses to noxious ligands (0–10 ng ml^−1^ of mouse recombinant SLPI (LifeSpan BioSciences, LS-G13637-10), 1 μM capsaicin or 50 mM KCl) were analysed at room temperature.

### Immunofluorescence

A total of 2 × 10^3^ DRG neurons were co-cultured with 2 × 10^4^ B16F10-mCherry-OVA cells for 24–48 h. The cells were fixed (4% PFA; 30 min), permeabilized (0.1 % Triton X-100; 20 min), and blocked (PBS, 0.1% Triton X-100 and 5% BSA; 30 min). The cells were rinsed (PBS), stained, and mounted with vectashield containing DAPI (Vector Laboratories, H-1000). Images were acquired using a Ti2 Nikon fluorescent microscope (IS-Elements Advanced Research v.4.5).

### Neurite length and ramification index

TRPV1^+^ nociceptors (*Trpv1*^*cre*^*::tdTomato*^*fl/WT*^) were cultured alone (2 × 10^3^ cells) or co-cultured (2 × 10^4^ cells) with B16F10-GFP, B16F0 or non-tumorigenic keratinocytes (MPEK-BL6). After 48 h, cells were fixed (see ‘Immunofluorescence’), and images were acquired using a Ti2 Nikon fluorescent microscope. The neurite length of TRPV1^+^ (tdTomato) neurons was measured using a neurite tracer macro in ImageJ (Fiji, v.1.53c) developed by the Fournier laboratory^63^, and the Schoenen ramification index (SRI) was measured by a Sholl analysis^64^ macro in ImageJ (Fiji, v.1.53c).

### Isolation of CD8^+^ T cells

Six-to-eight-week-old male and female mice were euthanized, and their spleens were collected in ice-cold PBS (5% FBS) and mechanically dissociated. The cells were strained (70 μm), RBC lysed (Life Technologies, A1049201; 2 min), and counted using a haemocytometer. Total CD8^+^ T cells were magnet sorted (Stem Cell, 19853A) and cultured (DMEM + FBS 10%, penicillin–streptomycin 1% + nonessential amino acids (Corning, 25-025-Cl) + vitamin + β-mercaptoethanol (Gibco, 21985-023) + l-glutamine (VWR, 02-0131) + sodium pyruvate (Corning, 25-000-Cl)). Cell purity was systematically confirmed after magnet sorting and the numbers of CD8^+^CD62L^hi^ were immunophenotyped by flow cytometry.

To generate cytotoxic T lymphocytes, 2 × 10^5^ CD8^+^ T cells were seeded and stimulated for 48 h under T_c1_ inflammatory conditions (2 μg ml^−1^ plate bounded anti-CD3 and anti-CD28 (Bio X Cell, BE00011, BE00151) + 10 ng ml^−1^ rIL-12 (BioLegend, 577008) + 10 μg ml^−1^ of anti-IL-4 (Bio X Cell, BE0045).

### In vitro stimulation of cytotoxic CD8^+^ T cells with neuron-conditioned medium

Naive or ablated DRG neurons were cultured (48 h) in Neurobasal-A medium supplemented with 0.05 ng μl^−1^ NGF (Life Technologies, 13257-019) and 0.002 ng μl^−1^ GDNF (PeproTech, 450-51-10). After 48 h, the neurobasal medium was removed, neurons were washed with PBS and 200 µl per well of T cell medium supplemented with 1 μl ml^−1^ peptidase inhibitor (Sigma, P1860) and, in certain cases, capsaicin (1 μM) or KCl (50 mM) was added to DRG neurons. The conditioned medium or vehicle were collected after 30 min and added to T_c1_ CD8^+^ T cells for another 96 h. The expression of exhaustion markers (PD-1, LAG3 and TIM3) and cytokines (IFNγ, TNF and IL-2) by CD8^+^ T cells was analysed by flow cytometry using an LSRFortessa or a FACSCanto II (Becton Dickinson). Cytokine expression levels were analysed after in vitro stimulation (PMA–ionomycin; see ‘Intracellular cytokine staining’).

### In vitro stimulation of cytotoxic CD8^+^ T cells with CGRP

CD8^+^ T cells were isolated and stimulated under T_c1_ conditions in a 96-well plate. After 48 h, cells were treated with either CGRP (0.1 μM) or PBS in the presence of peptidase inhibitor (1 μM) for another 96 h. The expression of PD-1, LAG3 and TIM3, as well as IFNγ, TNF and IL-2, was immunophenotyped by flow cytometry using an LSRFortessa or a FACSCanto II (Becton Dickinson). Cytokine expression levels were analysed after in vitro stimulation (PMA–ionomycin; see ‘Intracellular cytokine staining’).

### In vitro silencing of DRG neurons with BoNT/A

Naive DRG neurons (2 × 10^4^) were seeded in a 96-well plate with neurobasal medium supplemented with NGF and GDNF. Neurons were pre-treated with 50 pg ml^−1^ of BoNT/A for 24 h. After 24 h, the culture medium was removed, neurons were washed with PBS and 200 μl per well of T cell medium supplemented with 1 μl ml^−1^ peptidase inhibitor, and KCl (50 mM) was added to DRG neurons. The conditioned medium or vehicle were collected after 30 min and added to T_c1_ CD8^+^ T cells for another 96 h.

### In vitro RAMP1 blockade

CD8^+^ T cells were treated with CGRP_8–37_ (Tocris, 1169) 6 h before being exposed to the neuron-conditioned medium. In other instances, the neuron-conditioned medium was incubated for 1 h with 2 μg ml^−1^ of CGRP_8–37_ before being added to the CD8^+^ T cells.

### Co-culture of CD8^+^ T cells and DRG neurons

Naive DRG neurons (2 × 10^4^) were seeded in a 96-well-plate with T cell medium (supplemented with 0.05 ng μl^−1^ NGF (Life Technologies, 13257-019) and 0.002 ng μl^−1^ GDNF (PeproTech, 450-51-10)). One day after, T_c1_ CD8^+^ cells (1 × 10^5^) were added to the neurons in the presence of IL-2 (BioLegend, 575408). In some instances, co-cultures were stimulated with either capsaicin (1 μM) or KCl (50 mM). After 96 h, the cells were collected by centrifugation (5 min at 1,300 rpm), stained and immunophenotyped by flow cytometry using an LSRFortessa or a FACSCanto II (Becton Dickinson). Cytokine expression levels were analysed after in vitro stimulation (PMA–ionomycin; see ‘Intracellular cytokine staining’).

### RNA sequencing of triple co-cultures and data processing

A total of 1 × 10^4^ naive *Trpv1*^*cre*^*::CheRiff-eGFP *^*fl/WT*^ DRG neurons were co-cultured with 1 × 10^5^ B16F10-mCherry-OVA overnight in T cell medium (supplemented with 0.05 ng μl^−1^ NGF (Life Technologies, 13257-019), 0.002 ng μl^−1^ GDNF (PeproTech, 450-51-10). One day after, 4 × 10^5^ stimulated OVA-specific CD8^+^ T cells under T_c1_ conditions were added to the co-culture. After 48 h, the cells were detached and TRPV1 neurons (CD45^−^eGFP^+^mCherry−), B16F10-mCherry-OVA (CD45^−^eGFP^−^mCherry^+^) and OVA-specific CD8^+^ T cells (eGFP^−^mCherry^−^CD45^+^CD3^+^CD8^+^) were FACS purified using a FACSAria IIu cell sorter (Becton Dickinson), and the cell supernatant was collected for ELISAs.

RNA-sequencing libraries were constructed using the Illumina TruSeq Stranded RNA LT Kit (Illumina) following the manufacturer’s instructions. Illumina sequencing was performed at Fulgent Genetics. Reads were aligned to the Mouse mm10 (GenBank assembly accession GCA_000001635.2) reference genome using STAR v.2.7 (ref. ^58^). Aligned reads were assigned to genic regions using the featureCounts function from subread v.1.6.4 (ref. ^65^). Gene expression levels were represented by TPM. Hierarchical clustering was computed using the heatmap.2 function (ward.D2 method) from the gplots R package (v.3.1.3). Differential gene expression analysis was performed using DeSeq2 v.1.28.1 (ref. ^59^). These data have been deposited in the NCBI’s GEO (ref. ^60^) (GSE205864).

### RNA sequencing of cancer and neuron co-cultures and data processing

A total of 1 × 10^4^ naive *Trpv1*^*cre*^*::-CheRiff-eGFP*^*fl/WT*^ DRG neurons were co-cultured with 5 × 10^4^ B16F10-mCherry-OVA cells overnight in complete DMEM (Corning, 10-013-CV) supplemented with 10% FBS (Seradigm, 3100), 1% penicillin–streptomycin (Corning, MT-3001-Cl), 0.05 ng μl^−1^ NGF (Life Technologies, 13257-019), 0.002 ng μl^−1^ GDNF (PeproTech, 450-51-10). After 48 h, the cells were detached and TRPV1 neurons (eGFP^+^mCherry^−^) and B16F10-mCherry-OVA (eGFP^−^mCherry^+^) were FACS purified using a FACSAria IIu cell sorter (Becton Dickinson), and the cell supernatant was collected for ELISAs.

RNA-sequencing libraries were constructed using the Illumina TruSeq Stranded RNA LT Kit (Illumina) following the manufacturer’s instructions. Illumina sequencing was performed at Fulgent Genetics. Reads were aligned to the mouse mm10 reference genome (GenBank assembly accession GCA_000001635.2) using STAR v.2.7 (ref. ^58^). Aligned reads were assigned to genic regions using the featureCounts function from subread v.1.6.4 (ref. ^65^). Gene expression levels were represented by TPM. Hierarchical clustering was computed using the heatmap.2 function (ward.D2 method) from the gplots R package (v.3.1.3). Differential gene expression analysis was performed using DeSeq2 v.1.28.1^59^. These data have been deposited in the NCBI’s GEO (ref. ^60^) (GSE205865).

### ELISA on co-cultures of B16F10 cells and DRG neurons

A total of 1 × 10^4^ naive DRG neurons were cultured (96 h) with and without 5 × 10^4^ B16F10 cells in complete DMEM (10% FBS, 1% penicillin–streptomycin, 1 μl ml^−1^ protease inhibitor). The cells were then challenged (30 min) with sterile PBS or KCl (40 mM) and the supernatant was collected. Neuropeptide releases were measured using commercial ELISAs for VIP (Antibodies Online, ABIN6974414), SP (Cayman Chemical, 583751) and CGRP (Cayman Chemical, 589001).

### ELISA on co-cultures of B16F10 cells, CD8^+^ T cells and DRG neurons

Levels of SLPI (R&D Systems, DY1735-05) were measured in the cells’ supernatant using a commercial ELISA.

### OT-I CD8^+^ T cell-induced B16F10 elimination

A total of 2 × 10^4^ naive *Trpv1*^*cre*^*::CheRiff-eGFP*^*fl/WT*^ DRG neurons were co-cultured with 1 × 10^5^ B16F10-mCherry-OVA cells overnight in T cell medium (supplemented with 0.05 ng μl^−1^ NGF (Life Technologies, 13257-019) and 0.002 ng μl^−1^ GDNF (PeproTech, 450-51-10)). One day after, 4 × 10^5^ stimulated OVA-specific CD8^+^ T cells under T_c1_ conditions were added to the co-culture. After 48 h, the cells were detached by trypsin (Gibco, 2062476) and collected by centrifugation (5 min at 1,300 rpm), stained using anti-Annexin V, 7-AAD (BioLegend, 640930) and anti-CD8 for 20 min at 4 °C, and immunophenotyped by flow cytometry using a FACSCanto II (Becton Dickinson). Cytokine expression levels were analysed after in vitro stimulation (PMA/ionomycin; see ‘Intracellular cytokine staining’).

### Effect of neuron-conditioned medium on OT-I CD8^+^ T cell-induced B16F10 elimination

A total of 4 × 10^5^ stimulated OVA-specific CD8^+^ T cells were added to 1 × 10^5^ B16F10-mCherry-OVA and treated with fresh condition medium (1:2 dilution). After 48 h, cells were stained using anti-Annexin V, 7-AAD (BioLegend, 640930) and anti-CD8 for 20 min at 4 °C, and were immunophenotyped by flow cytometry using an LSRFortessa or a FACSCanto II (Becton Dickinson). For CGRP, 4 × 10^5^ stimulated OVA-specific CD8^+^ T cells were added to 1 × 10^5^ B16F10- mCherry-OVA and treated with CGRP (100 nM). After 24 h, the cells were stained using anti-Annexin V, 7-AAD (BioLegend, 640930) and anti-CD8 for 20 min at 4 °C, and were immunophenotyped by flow cytometry using an LSRFortessa or a FACSCanto II (Becton Dickinson). Cytokine expression levels were analysed after in vitro stimulation (PMA–ionomycin; see ‘Intracellular cytokine staining’).

### Survival of B16F10 cells

A total of 1 × 10^5^ B16F10 cells were cultured in six-well plates and challenged with BoNT/A (0–50 pg μl^−1^) for 24 h, QX-314 (0–1%) for 72 h, BIBN4096 (1–8 μM) for 24 h or their vehicle. The survival of B16F10 cells was assessed using anti-Annexin V staining and measured by flow cytometry using an LSRFortessa or a FACSCanto II (Becton Dickinson), or counted using a haemocytometer.

### Effect of drugs on the function of CD8^+^ T cells

Splenocyte-isolated CD8^+^ T cells from naive C57BL6J mice were cultured under T_c1_-stimulating conditions (ex-vivo-activated by CD3 and CD28, IL-12 and anti-IL4) in 24-well plates for 48 h. The cells were then exposed to QX-314 (50–150 μM), BoNT/A (10–50 pg μl^−1^) or BIBN4096 (1–4 μM) for 24 h. Apoptosis, exhaustion and activation levels were measured by flow cytometry using an LSRFortessa or a FACSCanto II (Becton Dickinson).

### In silico analysis of neuronal expression profiles using RNA-sequencing and microarray datasets

Publicly available RNA gene expression data from seven datasets were downloaded from the NCBI GEO portal^66^. RNA gene expression values of genes of interest were extracted. Expression values from single-cell sequencing were averaged for all cells. To be able to compare expression from datasets that were generated using different techniques (single-cell RNA sequencing, bulk RNA sequencing and microarrays) and normalization methods (TPM, RPKM (reads per kilobase per million mapped reads), RMA (robust multiarray analysis) and UMI (unique molecular identifiers)), all genes of interest were ratioed over *TRPV1* expression, then multiplied by 100, and the log_10_ values of these values were plotted as a heat map^66^. Kupari et al.^67^ used single-cell RNA sequencing of JNC neurons, whereas Usoskin et al.^68^. and Li et al.^69^. used single-cell RNA sequencing of lumbar neurons. Chiu et al.^70^ measured gene expression by microarrays of whole and FACS-sorted Na_V_1.8^+^ lumbar neurons. Goswami et al.^71^ performed RNA sequencing of TRPV1^+^ lumbar neurons, whereas Ray et al.^72^ performed RNA sequencing of human lumbar neurons.

### In silico analysis of tumour expression profiles of patients with melanoma using single-cell RNA sequencing

Using the publicly available Broad Institute single-cell bioportal, we performed an in silico analysis of single-cell RNA sequencing of human melanoma biopsies. We assessed the gene profile of *RAMP1*-expressing and *RAMP1*-negative T cells in the tumours of patients with metastatic melanoma^42^. Similarly, we assessed the genetic program of *RAMP1*-expressing and *RAMP1*-negative CD8^+^ T cells in patients with melanoma^41^. The latter dataset was also used to analyse the genetic profile of CD8^+^ T cells in patients who were responsive to immune checkpoint blockers or unresponsive to such treatment, as well as the genetic profile of malignant melanoma cells (defined as CD90^−^CD45^−^) from the biopsies of ten different patients^41^. Individual cell data are shown as a log_2_-transformed 1 + (TPM/10). Experimental details and cell clustering have been defined in previous studies^41,42^.

### In silico analysis of the expression profiles of human immune cells

Publicly available RNA gene expression data from a previous study^73^ were downloaded from the NCBI GEO portal. Read counts normalized to transcripts per million protein-coding genes (pTPM) values for genes of interest were extracted. Expression values from single-cell sequencing were averaged for all cells. Experimental details and cell clustering have been defined before^73^.

### In silico analysis of the expression profiles of cultured B16F10 cells

Publicly available RNA gene expression data from a previous study^74^ were downloaded from the NCBI GEO portal. Read counts normalized to TPM for genes of interest were extracted. Experimental details and cell clustering have been defined before^74^.

### In silico analysis of the expression profiles of mouse immune cells using the ImmGen database

Using the publicly available ImmGen database, we performed an in silico analysis of RNA-sequencing data (DESeq2 data) of various mouse immune cells. As per ImmGen protocol, RNA-sequencing reads were aligned to the mouse genome GENCODE GRCm38/mm10 primary assembly (GenBank assembly accession GCA_000001635.2) and gene annotations vM16 with STAR 2.5.4a. The ribosomal RNA gene annotations were removed from the general transfer format file. The gene-level quantification was calculated by featureCounts. Raw read count tables were normalized by the median of ratios method with the DESeq2 package from Bioconductor and then converted to GCT and CLS format. Samples with fewer than 1 million uniquely mapped reads were automatically excluded from normalization. Experimental details can be found at https://www.immgen.org/Protocols/ImmGenULI_RNAseq_methods.pdf.

### Oncomine

In silico analysis of the expression profiles of biopsies from patients with melanoma using bulk microarray sequencing. As described previously^19^, samples from 45 cutaneous melanomas and 18 benign melanocytic skin nevus biopsies (around 5–20 μm) were collected and amplified, and their transcriptomes were profiled using Affymetrix U133A microarrays. Data were downloaded from the Oncomine database (https://www.oncomine.com/) as log_2_-transformed (median centred intensity) and genes of interest were shown as heat maps. Experimental details and cell clustering have been defined before^19^.

### Survival analysis of patients with melanoma

OncoLnc (http://www.oncolnc.org/) contains survival data for 8,647 patients from 21 cancer studies performed by TCGA^40^. Using OncoLnc, we assessed the transcript expression of a user-defined list of 333 neuronal-enriched genes (neuronal membrane proteins, neural stem cell markers, transcription factors, ion channel receptors and neuropeptides) in 459 skin cancer (SKCM) tumour biopsies from the TCGA database. Of these genes, 206 were expressed, and 108 were selected on the basis of their negative Cox coefficient value, indicating a link between lower gene expression and improved patient survival. Kaplan–Meier curves show the survival of the patients after segregation into two groups defined by their low or high expression of a gene of interest. Details of patients can be found in TCGA^40^ and computational analyses can be found at 10.7717/peerj-cs.67.

### Data collection and analysis

GraphPad Prism (v.9.0) and Microsoft Excel (v.2019) were used for data entry, graph construction and data analysis. Image analysis (neurite length and ramification index) was performed using ImageJ macros (Fiji, v.1.53c). Flow cytometry data were analysed using FlowJo (v.10.0.0). Calcium microscopy analysis was performed using a Nikon Eclipse Ti2 microscope (NIS-Elements Advanced Research v.4.5). Patient biopsy images were collected using an Olympus BX51 bright-field microscope and mouse tumour innervation images were acquired using an Olympus FV3000 confocal imaging system. For RNA sequencing, the reads were aligned to the mouse reference genome GRCm38/mm10 (GenBank assembly accession GCA_000001635.2) using STAR (versions used: 2.5.4a, 2.5.1b and 2.7). Aligned reads were assigned to genic regions using the featureCounts function from Subread (v.1.6.4 22). Hierarchical clustering was computed using the heatmap.2 function (ward.D2 method) from Gplots R package (v.3.1.3). Differential gene expression analysis was carried out by DeSeq2 (versions used: 1.18.1 and 1.28.1). TCGA data were accessed using Oncomine (https://www.oncomine.com/ for gene expression) and OncoLnc (http://www.oncolnc.org/ for survival). Single-cell RNA sequencing was analysed using the Broad Single-Cell Portal (https://singlecell.broadinstitute.org). Human and mouse immune cell gene profiles were respectively analysed using the Human Protein Atlas (https://www.proteinatlas.org/humanproteome/immune+cell) and Immunological Genome Project (https://www.immgen.org/).

### Sample size

Statistical methods were not used to predetermine sample size. The size of the cohort, based on similar studies in the field, was validated by pilot studies. All sample sizes are indicated in the figures and/or figure legends. All *n* values are indicated within the figure legends. In the only case in which a range is used (Fig. 2a,b), exact *n* values are provided in the source data files. For in vivo experiments, we used *n* > 8 mice. For in vitro experiments in which replicate samples were used, we repeated the experiments at least three independent times to confirm the findings. For other mouse experiments a minimum of five mice were used to ensure that proper statistics could be used. We determined this to be sufficient as per our pilot data, use of internal controls and/or the observed variability between within experimental groups.

### Replication

The number of replicates is indicated in the figures, figure legends and/or methods. On the graphs, individual dots represent individual samples or mice used. For each experiment, all attempts at replication were successful and our findings showed comparable results.

### Randomization

Breeding pairs and their offspring (nociceptor-intact and -ablated mice) were co-housed and, in respect with the ARRIVE guidelines^75^, were randomly allocated into each experimental group. For in vitro experiments, randomization was used for treatment selection. In some calcium microscopy experiments, the investigators performing the data collection were tasked to select all ligand-responsive cells for downstream analysis. In these rare cases. randomization was not used for cell selection.

### Blinding

Double blinding was used for all in vivo treatments. In calcium microscopy experiments involving co-culture (for example, nociceptors and cancer cells), the differences in cell morphology are obvious and, therefore, the investigator performing the experiment was not blind. However, this investigator was always blinded to the treatment being applied to the cells and a second blinded investigator performed the downstream data analysis.

### Data exclusions

No data were excluded.

### Statistics

Statistical significance was determined using GraphPad Prism (Dotmatics, v.9) and calculated using simple linear regression analysis, Mantel–Cox regression, one-way or two-way ANOVA for multiple comparisons and two-sided unpaired Student’s *t*-test for single variable comparison. In calcium imaging experiments, the *P* value is calculated on ligand-responsive neurons (calcium flux ≥ 5–10%). *P* values < 0.05 were considered significant.

### Antibodies

All of the antibodies used in this study are also listed in Supplementary Table 2.

### Reporting summary

Further information on research design is available in the Nature Research Reporting Summary linked to this article.

## Online content

Any methods, additional references, Nature Research reporting summaries, source data, extended data, supplementary information, acknowledgements, peer review information; details of author contributions and competing interests; and statements of data and code availability are available at 10.1038/s41586-022-05374-w.

## Supplementary information

Supplementary Tables This file contains Supplementary Tables 1-2

Reporting Summary

Peer Review File

## Data Availability

All data are readily available online (https://www.talbotlab.com/nature) and from the corresponding author. The RNA-sequencing datasets have been deposited in the NCBI’s GEO (GSE205863, GSE205864 and GSE205865). Source data are provided with this paper.

## Source data

Source Data Fig. 1
Source Data Fig. 2
Source Data Fig. 3
Source Data Fig. 4
Source Data Fig. 5
Source Data Extended Data Fig. 1
Source Data Extended Data Fig. 2
Source Data Extended Data Fig. 3
Source Data Extended Data Fig. 4
Source Data Extended Data Fig. 5
Source Data Extended Data Fig. 6
Source Data Extended Data Fig. 7
Source Data Extended Data Fig. 8
Source Data Extended Data Fig. 9
Source Data Extended Data Fig. 10
Source Data Extended Data Fig. 11
