# Supplementary material for: Nociceptor neurons affect cancer immunosurveillance
Source: Nature. 2022 Nov 2;611(7935):405–12. doi: 10.1038/s41586-022-05374-w (PMC9646485; doi:10.1038/s41586-022-05374-w)
Supplement: Supplementary file 1 — This file contains Supplementary Tables 1-2 [file 41586_2022_5374_MOESM1_ESM.pdf]

nature portfolio

https://doi.org/10.1038/s41586-022-05374-w

Supplementary information

Nociceptor neurons affect cancer immunosurveillance

In the format provided by the authors and unedited

Specimen	Stage	Type	Location	Breslow (mm)	Ulceration
HN480	pT4a	Nodular	R. temple	7 to 0.7	No
DERM 103	pT1a	Superficial spreading	L. post. shoulder	0.7	No
DERM 105	pT2a	Nevoid	R. flank	1.1	No
DERM 106	pT2a	NOS	L. thigh	1.2	No
DERM 107	pT2a	NOS	L. upper outer arm	1.6	No
DERM 110	pT1b	Nevoid	L. lat. upper arm	0.81	No
DERM 112	pT4b	Nodular	L. postaur. neck	11.3	Yes
DERM 113	pT4b	NOS	Vulva	at least 19	Yes
DERM 114	pT4a	Nodular	R. shoulder	16	No
DERM 115	pT2a	i.d. congenital nevus	L. back	2.8 and 0.8mm	No

Specimen	Clark level	TILs	Mitotic Index (per mm^2)	Distant Mets
HN480	IV	"Present, nonbrisk"	10	No
DERM 103	NC	NC	"Low"	No
DERM 105	III	"Slight to moderate"	"Low"	No
DERM 106	IV	NC	"Low"	No
DERM 107	IV	"Slight to moderate"	3 ("High")	No
DERM 110	IV	"Slight to moderate"	2 ("High")	No
DERM 112	V	"Present, nonbrisk"	11	Liver
DERM 113	NC	No	5 ("High")	Liver
DERM 114	V	"Slight to moderate"	4 ("High")	Liver, lung
DERM 115	IV	"Sparse"	1 "Low"	Brain

Supplementary Table 1. Board-certified pathologist notes on the specimens used in this study.

Antibody	Company		Clone/cat number	Dilution/concentration
anti-AnnexinV-APC and 7-AAD	BioLegend	cat no:	640930	1:100
anti-CD11b-APC/Cy7	BioLegend	cat no:	101226	1:100
anti-CD16/32	Biolegend	cat no:	156604	1:100
anti-CD28	Bio X Cell	cat no:	BE0015-5	1:4000
anti-CD3	Bio X Cell	cat no:	BE0001-1	200µg/mouse
anti-CD3	Bio X Cell	cat no:	BE00011	1:3000
anti-CD45.1-BV421	BioLegend	cat no:	110732	1:100
anti-CD45.2-BV650	BioLegend	cat no:	109836	1:100
anti-CD45-Alexa Fluor 700	BioLegend	cat no:	103128	1:100
anti-CD45-BV421	BioLegend	cat no:	103134	1:100
anti-CD4-FITC	BioLegend	cat no:	100406	1:100
anti-CD4-PerCP/Cyanine5.5	BioLegend	cat no:	100540	1:100
anti-CD8	Bio X Cell	cat no:	BP0061	200µg/mouse
anti-CD8-AF700	BioLegend	cat no:	100730	1:100
anti-CD8-BV421	BioLegend	cat no:	100753	1:100
anti-CD8-Pacific Blue	BioLegend	cat no:	100725	1:100
anti-CD8-PerCP/Cyanine5.5	BioLegend	cat no:	100734	1:100
anti-GFP	Aves Labs	cat no:	GFP-1020	1:500
anti-H-2Kb/OVA257-264	NIH tetramer core facility	cat no:	IEDB ID: 58560	1:100
anti-IFN-γ-APC	BioLegend	cat no:	505810	1:100
anti-IFN-γ-FITC	BioLegend	cat no:	505806	1:100
anti-IgG(H+L)-AF488	Invitrogen	cat no:	A28175	1:500
anti-IL-2-BV510	BioLegend	cat no:	503833	1:100
anti-IL-2-Pacific Blue	BioLegend	cat no:	503820	1:100
anti-IL2-Pecy7	BioLegend	cat no:	503832	1:100
anti-IL4	Bio X Cell	cat no:	BE0045	0.1:1000 - 1:1000
anti-Lag3-PE	BioLegend	cat no:	125208	1:100
anti-Lag3-PerCP/Cyanine5.5	BioLegend	cat no:	125212	1:100
anti-mCherry	OriGene	cat no:	AB0040-200	1:500
anti-PD1-PE-Cy7	BioLegend	cat no:	329917	1:100
anti-PDL1	Bio X Cell	cat no:	BE0101	6mg/kg
anti-Tim3-APC	BioLegend	cat no:	119706	1:100
anti-TNFα-BV510	BioLegend	cat no:	506339	1:100
anti-TNFα-BV711	BioLegend	cat no:	506349	1:100
anti-TNFα-PE	BioLegend	cat no:	506306	1:100
anti-TRPV1	Alomone Labs	cat no:	ACC-030	1:100
DAPI	Vector Laboratories	cat no:	H-1000	1:2000
Viability Dye-eFluor780	eBioscience	cat no:	65-0865-14	1:1000
ZombieAqua	BioLegend	cat no:	423102	1:100

Supplementary Table 2. A list of all the antibodies used for *in vitro* immune cell activation, *in vivo* cell depletion or neutralisation, flow cytometry, IHC or iDISCO experiments. The list details the clone/catalog number, commercial provider and dilution/concentration/dose used.
