# Supplementary material for: Nociceptor neurons affect cancer immunosurveillance
Source: Nature. 2022 Nov 2;611(7935):405–12. doi: 10.1038/s41586-022-05374-w (PMC9646485; doi:10.1038/s41586-022-05374-w)
Supplement: Supplementary file 2 — Reporting Summary [file 41586_2022_5374_MOESM2_ESM.pdf]

nature portfolio

Corresponding author(s): Sebastien Talbot

Last updated by author(s): Aug 13, 2022

Reporting Summary

Nature Portfolio wishes to improve the reproducibility of the work that we publish. This form provides structure for consistency and transparency in reporting. For further information on Nature Portfolio policies, see our Editorial Policies and the Editorial Policy Checklist.

Statistics

For all statistical analyses, confirm that the following items are present in the figure legend, table legend, main text, or Methods section.

n/a	Confirmed	
☐	☒	The exact sample size (*n*) for each experimental group/condition, given as a discrete number and unit of measurement
☐	☒	A statement on whether measurements were taken from distinct samples or whether the same sample was measured repeatedly
☐	☒	The statistical test(s) used AND whether they are one- or two-sided<br>*Only common tests should be described solely by name; describe more complex techniques in the Methods section.*
☐	☒	A description of all covariates tested
☐	☒	A description of any assumptions or corrections, such as tests of normality and adjustment for multiple comparisons
☐	☒	A full description of the statistical parameters including central tendency (e.g. means) or other basic estimates (e.g. regression coefficient) AND variation (e.g. standard deviation) or associated estimates of uncertainty (e.g. confidence intervals)
☐	☒	For null hypothesis testing, the test statistic (e.g. *F*, *t*, *r*) with confidence intervals, effect sizes, degrees of freedom and *P* value noted<br>*Give P values as exact values whenever suitable.*
☒	☐	For Bayesian analysis, information on the choice of priors and Markov chain Monte Carlo settings
☒	☐	For hierarchical and complex designs, identification of the appropriate level for tests and full reporting of outcomes
☐	☒	Estimates of effect sizes (e.g. Cohen's *d*, Pearson's *r*), indicating how they were calculated

Our web collection on statistics for biologists contains articles on many of the points above.

Software and code

Policy information about availability of computer code

Data collection	Cell were immunophenotyped using a LSRFortessa or FACSCanto II (Becton Dickinson).<br><br>Calcium microscopy analysis was performed using Nikon eclipse ti2 microscope (NIS-Elements Advanced Research version 4.5).<br><br>Patient biopsy images were collected using an Olympus BX51 bright-field microscope.<br><br>Mouse tumor innervation images were acquired using an Olympus FV3000 confocal imaging system.<br><br>GraphPad Prism v9 and Microsoft Excel (version 2019) was used for data entry, graph construction and data analysis.
Data analysis	GraphPad Prism (Version 9.0) and Microsoft Excel (version 2019) were used for data entry, graph construction, and data analysis.<br><br>Image analysis (neurite length, ramification index) was performed using ImageJ macros (Fiji; version 1.53c).<br><br>Calcium microscopy analysis was performed using Nikon eclipse ti2 microscope (NIS-Elements Advanced Research version 4.5).<br><br>Flow cytometry data were analyzed using FlowJo (version 10.0.0).<br><br>TCGA data were accessed via Oncomine (www.oncomine.com for gene expression) and OncoLnc (www.oncolnc.org for survival).<br><br>Single-cell RNA sequencing was analyzed using the Broad single-cell portal (https://singlecell.broadinstitute.org).<br><br>Human and mouse immune cell gene profiles were respectively analyzed using the human protein atlas (https://www.proteinatlas.org/humanproteome/immune+cell) and Immunological genome project (https://www.immgen.org/).

For RNA sequencing, the reads were aligned to the mouse reference genome GRCm38/mm10 (GenBank assembly accession GCA_000001635.2) using STAR (version used: 2.5.4a, 2.5.1b, 2.7).

Aligned reads were assigned to genic regions using the featureCounts function from Subread (version 1.6.4 22).

Hierarchical clustering was computed using the heatmap.2 function (ward.D2 method) from Gplots R package (version 3.1.3).

Differential gene expression analysis was carried out by DeSeq2 (version used: 1.18.1 or 1.28.1).

For manuscripts utilizing custom algorithms or software that are central to the research but not yet described in published literature, software must be made available to editors and reviewers. We strongly encourage code deposition in a community repository (e.g. GitHub). See the Nature Portfolio guidelines for submitting code & software for further information.

Data

Policy information about availability of data

All manuscripts must include a data availability statement. This statement should provide the following information, where applicable:

- Accession codes, unique identifiers, or web links for publicly available datasets
- A description of any restrictions on data availability
- For clinical datasets or third party data, please ensure that the statement adheres to our policy

All data are readily available online (https://www.talbotlab.com/nature), from the corresponding author (email: sebas.talbot@gmail.com) and within the paper and its Supplementary Information files.

The RNA sequencing datasets have been deposited in the National Center for Biotechnology Information's Gene Expression Omnibus (#GSE205863, #GSE205864, #GSE205865).

GSE205863 is available at: https://www.ncbi.nlm.nih.gov/geo/query/acc.cgi?acc=GSE205863

GSE205864 is available at: https://www.ncbi.nlm.nih.gov/geo/query/acc.cgi?acc=GSE205864

GSE205865 is available at: https://www.ncbi.nlm.nih.gov/geo/query/acc.cgi?acc=GSE205865

Mouse reference genome GRCm38/mm10 is available at www.ncbi.nlm.nih.gov/assembly/GCF_000001635.20

Oncomine is available at: www.oncomine.com

OncoLnc is available at: www.oncolnc.org

Broad single-cell bioportal is available at : https://singlecell.broadinstitute.org
Tirosh et al., is available at: https://singlecell.broadinstitute.org/single_cell/study/SCP11/melanoma-intra-tumor-heterogeneity
Jerby-Arnon is available at: https://singlecell.broadinstitute.org/single_cell/study/SCP109/melanoma-immunotherapy-resistance#study-summary

Human immune cell gene profiles are accessible at: www.proteinatlas.org/humanproteome/immune+cell

Mouse immune cell gene profiles are available at: www.immgen.org/

Meta-analysis of nociceptor neurons gene profiles are available at: www.talbotlab.com/dataset

Field-specific reporting

Please select the one below that is the best fit for your research. If you are not sure, read the appropriate sections before making your selection.

☒ Life sciences ☐ Behavioural & social sciences ☐ Ecological, evolutionary & environmental sciences

For a reference copy of the document with all sections, see nature.com/documents/nr-reporting-summary-flat.pdf

Life sciences study design

All studies must disclose on these points even when the disclosure is negative.

Sample size	Statistical methods were not used to predetermine sample size. The size of the cohort, based on similar studies in the field, was validated by pilot studies. All sample sizes are indicated in the figures and/or figure legends. All n values are clearly indicated within the figure legends. In the only case where a range is used (figure 2a/b), exact n values are provided in the source data files. For in vivo experiments we used n>8 animals. For in vitro experiments where replicate samples were used, we repeated the experiments at least 3 independent times to confirm the findings. For other mouse experiments a minimum of 5 mice were used to ensure proper statistics could be utilized. We determined this to be sufficient as per our pilot data, use of internal controls and/or the observed variability between within experimental groups.
Data exclusions	No data were excluded.

Replication	This is indicated in the figures, figure legends and/or methods. On the graphs individual dots represent individual samples/mice used. For each experiment, all attempts at replication were successful and our findings showed comparable results.
Randomization	Animals in a particular cohort were generated from one breeding pair and all offspring (e.g. nociceptor intact and ablated mice) were co-housed and, in respect with the ARRIVE guidelines, were randomly allocated into each experimental group. For in vitro experiments, randomization was used for treatment selection. In some calcium microscopy experiments, the investigators performing the data collection were tasked to select all ligands responsive cells for downstream analysis. In these rare cases. randomization was not used for cell selection.
Blinding	Double blind was used for all in vivo treatments. In calcium microscopy involving co-culture (e.g. nociceptors and cancer cells), the differences in cell morphology are obvious and, therefore, the investigator performing the experiment was not blind. However, these investigators were always blinded to the treatment being applied to the cells and a second blinded investigator performed the downstream data analysis.

Reporting for specific materials, systems and methods

We require information from authors about some types of materials, experimental systems and methods used in many studies. Here, indicate whether each material, system or method listed is relevant to your study. If you are not sure if a list item applies to your research, read the appropriate section before selecting a response.

Materials & experimental systems

n/a	Involved in the study
☐	☒ Antibodies
☐	☒ Eukaryotic cell lines
☒	☐ Palaeontology and archaeology
☐	☒ Animals and other organisms
☐	☒ Human research participants
☒	☐ Clinical data
☒	☐ Dual use research of concern

Methods

n/a	Involved in the study
☒	☐ ChIP-seq
☐	☒ Flow cytometry
☒	☐ MRI-based neuroimaging

Antibodies

Antibodies used

Antibody (company, catalog number, dilution, clone):

anti-AnnexinV-APC and 7-AAD (BioLegend), cat no: 640930, Dilution: 1:100
anti-CD11b-APC/Cy7 (BioLegend), cat no: 101226, Dilution: 1:100
anti-CD16/32 (Biolegend), cat no: 156604, Dilution: 1:100
anti-CD28 (Bio X Cell), cat no: BE0015-5, Dilution: 1:4000
anti-CD3 (Bio X Cell), cat no: BE0001-1, Dilution: 200µg/mouse
anti-CD3 (Bio X Cell), cat no: BE00011, Dilution: 1:3000
anti-CD45.1-BV421 (BioLegend), cat no: 110732, Dilution: 1:100
anti-CD45.2-BV650 (BioLegend), cat no: 109836, Dilution: 1:100
anti-CD45-Alexa Fluor 700 (BioLegend), cat no: 103128, Dilution: 1:100
anti-CD45-BV421 (BioLegend), cat no: 103134, Dilution: 1:100
anti-CD4-FITC (BioLegend), cat no: 100406, Dilution: 1:100
anti-CD4-PerCP/Cyanine5.5 (BioLegend), cat no: 100540, Dilution: 1:100
anti-CD8 (Bio X Cell), cat no: BP0061, Dilution: 200µg/mouse
anti-CD8-AF700 (BioLegend), cat no: 100730, Dilution: 1:100
anti-CD8-BV421 (BioLegend), cat no: 100753, Dilution: 1:100
anti-CD8-Pacific Blue (BioLegend), cat no: 100725, Dilution: 1:100
anti-CD8-PerCP/Cyanine5.5 (BioLegend), cat no: 100734, Dilution: 1:100
anti-GFP (Aves Labs), cat no: GFP-1020, Dilution: 1:500
anti-H-2Kb/OVA257-264 (NIH tetramer core facility), IEDB ID: 58560, Dilution: 1:100
anti-IFN-γ-APC (BioLegend), cat no: 505810, Dilution: 1:100
anti-IFN-γ-FITC (BioLegend), cat no: 505806, Dilution: 1:100
anti-IgG(H+L)-AF488 (Invitrogen), cat no: A28175, Dilution: 1:500
anti-IL-2-BV510 (BioLegend), cat no: 503833, Dilution: 1:100
anti-IL-2-Pacific Blue (BioLegend), cat no: 503820, Dilution: 1:100
anti-IL2-Pecy7 (BioLegend), cat no: 503832, Dilution: 1:100
anti-IL4 (Bio X Cell), cat no: BE0045, Dilution: 0.1:1000 - 1:1000
anti-Lag3-PE (BioLegend), cat no: 125208, Dilution: 1:100
anti-Lag3-PerCP/Cyanine5.5 (BioLegend), cat no: 125212, Dilution: 1:100
anti-mCherry (OriGene), cat no: AB0040-200, Dilution: 1:500
anti-PD1-PE-Cy7 (BioLegend), cat no: 329917, Dilution: 1:100
anti-PDL1 (Bio X Cell), cat no: BE0101, Dilution: 6mg/kg
anti-Tim3-APC (BioLegend), cat no: 119706, Dilution: 1:100
anti-TNFα-BV510 (BioLegend), cat no: 506339, Dilution: 1:100
anti-TNFα-BV711 (BioLegend), cat no: 506349, Dilution: 1:100
anti-TNFα-PE (BioLegend), cat no: 506306, Dilution: 1:100
anti-TRPV1 (Alomone Labs), cat no: ACC-030, Dilution: 1:100
DAPI (Vector Laboratories), cat no: H-1000, Dilution: 1:2000

Viability Dye-eFluor780 (eBioscience), cat no: 65-0865-14, Dilution: 1:1000
ZombieAqua (BioLegend), cat no: 423102, Dilution: 1:100

Validation

Anti-AnnexinV-APC and 7-AAD was validated for flow cytometry and for use in mouse cell lines by supplier, and previously used in ≥ 16 publications (Broggi A, et al. 2017. Nat Immunol. 18:1084)

Anti-CD11b-APC/Cy7 was validated for flow cytometry and for use in mouse cell lines by supplier, and previously used in ≥ 128 publications (Kleppe M et al. 2018. Cancer cell. 33(1):29-43)

Anti-CD16/32 was validated for flow cytometry and for use in mouse cell lines by supplier, and previously used in ≥ 12 publications (Oguri Y, et al. 2020. Cell. 182(3):563-577.e20)

Anti-CD28 was validated for in vitro T cell stimulation/activation by supplier, and previously used in ≥ 9 publications (Vegran F et al., 2014. Nat Immunology, 15(8):758-66)

Anti-CD3 was validated for in vitro T cell stimulation/activation by supplier, and previously used in ≥ 19 publications (Wendland K et al., 2018. J Immunol, 15;201(2):524-532)

Anti-CD3 was validated for in vivo T cell depletion in the mouse by supplier, and previously used in ≥ 19 publications (Peng B. et al., 2009. Blood, 12;114(20):4373-82)

Anti-CD45.1-BV421 was validated for flow cytometry and for use in mouse cell lines by supplier, and previously used in ≥ 22 publications (Phan TG, et al. 2007. Nature Immunol. 8:992)

Anti-CD45.2-BV650 was validated for flow cytometry and for use in mouse cell lines by supplier, and previously used in ≥ 11 publications (Kohlmeier JE, et al. 2008. Immunity. 29:101)

Anti-CD45-Alexa Fluor 700 was validated for flow cytometry and for use in mouse cell lines by supplier, and previously used in ≥ 96 publications (Radtke AJ, et al. 2022. Nat Protoc. 17:378-401)

Anti-CD45-BV421 was validated for flow cytometry and for use in mouse cell lines by supplier, and previously used in ≥ 50 publications (Haynes NM, et al. 2007. J. Immunol. 179:5099)

Anti-CD4-FITC was validated for flow cytometry and for use in mouse cell lines by supplier, and previously used in ≥ 100 publications (Zheng B, et al. 1996. J. Exp. Med. 184:1083)

Anti-CD4-PerCP/Cyanine5.5 was validated for flow cytometry and for use in mouse cell lines by supplier, and previously used in ≥ 48 publications (León-Ponte M, et al. 2007. Blood 109:3139)

Anti-CD8 was validated for cell depletion by supplier, and previously used in ≥ 19 publications (Vegran, F., et al. (2014) Nat Immunol 15(8): 758-766)

Anti-CD8-AF700 was validated for flow cytometry and for use in mouse cell lines by supplier, and previously used in ≥ 54 publications (Shih FF, et al. 2006. J. Immunol. 176:3438)

Anti-CD8-BV421 was validated for flow cytometry and for use in mouse cell lines by supplier, and previously used in ≥ 43 publications (Bouwer HGA, et al. 2006. P. Natl. Acad. Sci. USA 103:5102)

Anti-CD8-Pacific Blue was validated for flow cytometry and for use in mouse cell lines by supplier, and previously used in ≥ 38 publications (Ko SY, et al. 2005. J. Immunol. 175:3309)

Anti-CD8-PerCP/Cyanine5.5 was validated for flow cytometry and for use in mouse cell lines by supplier, and previously used in ≥ 110 publications (Bankoti J, et al. 2010. Toxicol. Sci. 115:422)

Anti-GFP was validated for flow cytometry and for use in mouse cell lines by supplier and previously used in ≥ 49 publications (Zimmerman A et al., 2019 Neuron. 102(2):420-434.e8)

Anti-H-2Kb/OVA257-264 was validated for flow cytometry by the NIH tetramer core and previously used in Crittenden et al., 2018, Sci Rep. 3;8(1):7012

Anti-IFN-γ-APC was validated for flow cytometry and for use in mouse cell lines by supplier and previously used in ≥ 142 publications (Ferrick D, et al. 1995. Nature 373:255)

Anti-IFN-γ-FITC was validated for flow cytometry and for use in mouse cell lines by supplier and previously used in ≥ 74 publications (Ko SY, et al. 2005. J. Immunol. 175:3309)

Anti-IgG(H+L)-AF488 was validated for immunofluorescence by supplier and previously used in ≥ 305 publications (Miao et al., 2022. J Exp Med. 5;219(9):e20220214)

Anti-IL-2-BV510 was validated for flow cytometry and for use in mouse cell lines by supplier and previously used in ≥ 2 publications (Dikiy S, et al. 2021. Immunity. 54(5):931-946.e11)

Anti-IL-2-Pacific Blue was validated for flow cytometry and for use in mouse cell lines by supplier and previously used in Mohammed RN, et al. 2019. Sci Rep. 4.185416667

Anti-IL2-Pecy7 was validated for flow cytometry and for use in mouse cell lines by supplier and previously used in ≥ 5 publications (Xu W, et al. 2021. Immunity. 54(3):526-541.e7)

Anti-IL4 was validated for neutralisation by supplier and previously used in ≥ 19 publications (Tang W, et al. Immunity. 2014 Oct 16;41(4):555-66)

Anti-Lag3-PE was validated for flow cytometry and for use in mouse cell lines by supplier and previously used in ≥ 14 publications (Dong MB, et al. 2020. Cell. 178(5):1189-1204.e23)

Anti-Lag3-PerCP/Cyanine5.5 was validated for flow cytometry and for use in mouse cell lines by supplier and previously used in ≥ 10 publications (Vardhana SA, et al. 2020. Nat Immunol. 1.584722222)

Anti-mCherry was validated for immunofluorescence by supplier and previously used in ≥ 12 publications (Abraira et al., Cell. 2017 168(1-2):295-310.e19)

Anti-PD1-PE-Cy7 was validated for flow cytometry and for use in mouse cell lines by supplier and previously used in ≥ 29 publications (Barili V, et al. 2020. Nat Commun. 0.877777778)

Anti-PDL1 was validated for in vivo PD-L1 blockade by supplier and previously used in ≥ 18 publications (Twyman-Saint Victor C, et al., Nature 2015. 16;520(7547):373-7)

Anti-Tim3-APC was validated for flow cytometry and for use in mouse cell lines by supplier and previously used in ≥ 6 publications (Mooney L, et al. 2002. Nature 415:536)

Anti-TNFα-BV510 was validated for flow cytometry and for use in mouse cell lines by supplier and previously used in ≥ 8 publications (Li C, et al. 2020. Immunity. 52(1):201-202)

Anti-TNFα-BV711 was validated for flow cytometry and for use in mouse cell lines by supplier and previously used in ≥ 6 publications (Routhu NK, et al. 2021. Immunity. 54(3):542-556.e9)

Anti-TNFα-PE was validated for flow cytometry and for use in mouse cell lines by supplier and previously used in ≥ 55 publications (Logan K Smith et al. 2018. Immunity. 48(2):299-312)

Anti-TRPV1 was validated for immunohistochemistry and for use in human sample staining by supplier and previously used in ≥ 167 publications (Nam, J.H. et al. (2015) Brain 138, 3610)

DAPI was validated for immunofluorescence and for use in mouse sample staining by supplier and previously used in ≥ 9300 publications (Bae E et al., 2022. Nature Comm. 25;13(1):4268)

Viability Dye-eFluor780 was validated for flow cytometry and for use in mouse cell lines by supplier and previously used in Mathur et al., JCI Insight. 2021. 22;6(24):e148510.

ZombieAqua was validated for flow cytometry and for use in mouse cell lines by supplier and previously used in ≥ 174 publications (Barry KC, et al. 2018. Nat Med. 24:1178)

Eukaryotic cell lines

Policy information about cell lines

Cell line source(s)

B16F0 (ATCC, #CRL-6322)
B16F10 (ATCC, #CRL-6475)
B16F10-OVA (Matthew F. Krummel, UCSF)
B16F10-OVA-mCherry (Matthew F. Krummel, UCSF)
B16F10-eGFP (Imanis, #CL053)
YUMM1.7 (ATCC, #CRL-3362)
YUMMER1.7 (Marcus Bosenberg, Yale U)
Non-tumorigenic keratinocytes (CellnTEC, #MPEK-BL6100)

Authentication

Non-commercial cell lines (B16F10-OVA, B16F10-OVA-mCherry and B16F10-eGFP) were authenticated using antibody (against OVA, eGFP, mCherry) and/or imaging as well as morphology and/or growth property. Commercial cell line (ATCC, Imanis, CellnTec) provides a certificate of analysis in which they validate the cell lines with specific test and procedures such as growth property, morphology, mycoplasma detection, species determination, and sterility test.

Mycoplasma contamination

All the cell lines tested negative for mycoplasma

Commonly misidentified lines
(See ICLAC register)

No commonly misidentified lines were used in this study.

Animals and other organisms

Policy information about studies involving animals; ARRIVE guidelines recommended for reporting animal research

Laboratory animals

Both males and females mice (mus musculus) were used equally, and they were used at 6 weeks of age up to 20 weeks of age. Mice were housed in standard environmental conditions (12h light/dark cycle; 23oC; food and water ad libitum) at facilities accredited by the Canadian Council of Animal Care (UdeM) or Association for Assessment and Accreditation of Laboratory Animal Care (BCH).

C57BL6J (Jax, #000664); CD45.1+ C57BL6J (Jax, #002014), RAMP1-/- (Jax, #031560), Rag1-/- (Jax, #002216), OT1 (Jax, #003831), TRPV1cre (Jax, #017769), ChR2fl/fl (Jax, #012567), td-tomatofl/fl (Jax, #007908), DTAfl/fl (Jax, #009669), QuASR2fl/fl (Jax, #028678) mice were purchased from Jackson Laboratory. NaV1.8cre mice were generously supplied by Professor Rohini Kuner (Heidelberg University) and Professor John Wood (UCL). Excluding CD45.1+ mice, all other lines were backcrossed >6 generations on C57BL6/J background.

We used the cre/lox toolbox to engineer the various mice lines used (TRPV1cre::DTAfl/wt, TRPV1cre::QuASR2fl/wt, TRPV1cre::Tdtomatofl/wt, NaV1.8cre::DTAfl/wt, NaV1.8cre::ChR2fl/wt and littermate control) by crossing heterozygote Cre mice with homozygous loxP mice. Mice of both sexes were used in the various cross. All Cre driver lines used were viable and fertile, and abnormal phenotypes were not detected. Offspring were tail-clipped; tissue was used to assess the presence of transgene by standard PCR, as described by Jackson Laboratory or the donating investigator.

Wild animals	The study did not involve any wild animals
Field-collected samples	The study did not involve any field-collected samples
Ethics oversight	The Institutional Animal Care and Use Committees of Boston Children's Hospital and the Université de Montréal (CDEA: #21046; #21047) approved all animal procedures.

Note that full information on the approval of the study protocol must also be provided in the manuscript.

Human research participants

Policy information about studies involving human research participants

Population characteristics

In compliance with all the relevant ethical regulation and as approved by Sanford Health IRB protocol #640, ten fully de-identified FFPE melanoma blocks were randomly selected for secondary use research specimens. Described below is the clinical characteristics of these specimens:

DERM103 patient sample was from a malignant melanoma of the shoulder with evidence of dense lymphohiostiocytic inflammatory infiltrate; mitotic index was low, staging was pT1a with negative lymph nodes

DERM105 patient sample was from a malignant melanoma of the posterior shoulder with slight to moderate inflammatory infiltrate; mitotic index was low, staging was pT2a with negative lymph nodes

DERM106 patient sample was a malignant melanoma of the thigh with negative lymph nodes. Breslow's thickness 1.2mm and negative lymph nodes. Mitotic index was low, staging pT2a with negative lymph nodes

DERM107 patient sample was a malignant melanoma from the upper arm, Breslow's thickness 1.6mm, Clark level IV, lymph nodes were negative, margins were clear, mitotic index was high, staging pT2a with negative lymph nodes

DERM110 patient sample is a malignant melanoma of the arm, Breslow's thickness 0.81 mm, Clark level IV, negative lymph nodes, staging pT1b, pN0, mitotic index high

DERM112 patient sample is a metastatic malignant melanoma which metastasized to the neck and liver, mitotic index high, staging pT4b with negative lymph nodes.

DERM113 patient sample is a metastatic malignant melanoma which metastasized to the vulva and liver, mitotic index high, staging pT4b with negative lymph nodes.

DERM114 patient sample is a metastatic malignant melanoma which metastasized to the shoulder, lung and liver. Mitotic index was high with positive lymph nodes. Staging pT4a.

DERM115 patient sample is a metastatic malignant melanoma which metastasized to the back and brain. Mitotic index was low with positive lymph nodes, staging pT2a.

HN480 patient sample is a malignant melanoma of the right temple, Breslow's thickness 7.0 mm, Clark level IV, negative lymph nodes, staging pT4a, mitotic index of 10

Recruitment	No patients were recruited for this study.
Ethics oversight	The ten melanoma samples used in this study were collected by Sanford Health and classified by a board-certified pathologist. Their secondary use as research specimen (fully de-identified FFPE blocks) was approved under Sanford Health IRB protocol #640 (Titled: understanding and improving cancer treatment of solid tumors). As part of this IRB-approved retrospective tissue analysis, and in accordance with US Department of Health and human services (HHS) secretary's advisory committee on human research protections, no patient consent was necessary as these secondary use specimens were free of linkers/identifiers and posed no more than minimal risk to human subjects.

Note that full information on the approval of the study protocol must also be provided in the manuscript.

Flow Cytometry

Plots

Confirm that:

☒ The axis labels state the marker and fluorochrome used (e.g. CD4-FITC).

☒ The axis scales are clearly visible. Include numbers along axes only for bottom left plot of group (a 'group' is an analysis of identical markers).

☒ All plots are contour plots with outliers or pseudocolor plots.

☒ A numerical value for number of cells or percentage (with statistics) is provided.

Methodology

Sample preparation

Immunophenotyping tumor and tumor-draining lymph node. Mice were euthanized when the tumor reached a volume of 800-1500 mm3. Tumors and their draining lymph nodes (tdLN) were harvested. Tumors were enzymatically digested in DMEM + 5% FBS (Seradigm, #3100) + 2 mg/mL collagenase D (Sigma, #11088866001) + 1 mg/mL Collagenase IV (Sigma, #C5138-1G) + 40 ug/mL DNAse I (Sigma, #10104159001) under constant shaking (40 min, 37oC). The cell suspension was centrifuged at 400 g for 5 min. The pellet was resuspended in 70% Percoll gradient (GE Healthcare), overlaid with 40% Percoll, and centrifuged at 500g for 20 min at room temperature with acceleration and deceleration at 1. The cells were aspirated from the Percoll interface and passed through a 70-µm cell strainer.

Tumor-draining lymph nodes were dissected in PBS + 5% FBS, mechanically dissociated using a plunger, strained (70µm), and washed with PBS.

Single cells were resuspended in FACS buffer (PBS, 2% FCS, EDTA), Fc blocked (0.5 mg/mL, 15 min; BD Biosciences, #553141) and stained (15 min, RT) with ZombieAqua (BioLegend, #423102) or (15 min, RT) a Viability Dye eFluor 780 (eBioscience, #65-0865-14). The cells were then stained (30min, 4oC) with either of anti-CD45-BV421 (1:100, BioLegend, #103134), anti-CD45.1-BV421 (1:100, BioLegend, #110732), anti-CD45.2-BV650 (1:100, BioLegend, #109836), anti-CD45-Alexa Fluor 700 (1:100, BioLegend, #103128), anti-CD11b-APC/Cy7 (1:100, BioLegend, #101226), anti-CD8-AF700 (1:100, BioLegend, #100730), anti-CD8-BV421 (1:100, BioLegend, #100753), anti-CD8-PerCP/Cyanine5.5 (1:100, BioLegend, #100734), anti-CD8-Pacific Blue (1:100, BioLegend, #100725), anti-CD4-PerCP/Cyanine5.5 (1:100, BioLegend, #100540), anti-CD4-FITC (1:100, BioLegend, #100406), anti-PD-1-PE-Cy7 (1:100, BioLegend, #109110), anti-Lag3-PE (1:100, BioLegend, #125208), anti-Lag3-PerCP/Cyanine5.5 (1:100, BioLegend, #125212), anti-Tim-3-APC (1:100, BioLegend, #119706), washed and analyzed using a LSRFortessa or FACSCanto II (Becton Dickinson). Antigen specific CD8+ T cells were stained with H-2Kb/OVA257-264 (NIH tetramer core facility) for 15 minutes at 37̊C and were than stained with surface markers.

Intracellular cytokine staining. Cells were stimulated (3h) with phorbol-12-myristate 13-acetate (PMA; 50 ng/mL, Sigma-Aldrich, #P1585), Ionomycin (1 µg/mL, Sigma-Aldrich, #I3909) and Golgi Stop (1:100, BD Biosciences, #554724). The cells were then fixed/permeabilized (1:100, BD Biosciences, #554714) and stained with anti-IFN-γ-APC (1:100, BioLegend, #505810), anti-IFN-γ-FITC (1:100, BioLegend, #505806), anti-TNFα-BV510 (1:100, BioLegend, #506339), anti-TNFα-BV5711 (1:100, BioLegend, #506349), anti-TNFα-PE (1:100, BioLegend, #506306), anti-IL2-Pecy7 (1:100, BioLegend, #503832), anti-IL-2-Pacific Blue (1:100, BioLegend, # 503820), anti-IL-2-BV510 (1:100, BioLegend, #503833), and analyzed using a LSR Fortessa or FACSCanto II (Becton Dickinson).

B16F10 survival. 1x105 B16F10 cells were cultured in 6-well-plate and challenged with BoNT/A (0-50 pg/µL) for 24h, QX-314 (0-1%) for 72h, BIBN4096 (1-4 µM) for 24h or their vehicle. B16F10 cell survival was assessed using anti-annexin V staining and measured by flow cytometry using a LSRFortessa or FACSCanto II (Becton Dickinson).

Drugs impact on CD8+ T-cells function. Splenocytes-isolated CD8+ T-cells from naive C57BL6 mice were cultured under Tc1-stimulating conditions (ex vivo activated by CD3/CD28, IL-12, and anti-IL4) in 24-well plate for 48h. The cells were then exposed to QX-314 (50-150 µM), BoNT/A (10-50 pg/µL) or BIBN4096 (1-4 µM) for 24h. Apoptosis, exhaustion and activation were measured by flow cytometry using a LSRFortessa or FACSCanto II (Becton Dickinson).

In vitro cytotoxic CD8+ T-cell stimulation with CGRP. CD8+ T-cells were isolated and stimulated under Tc1 condition in 96 wells plate. After 48h, cells were treated with either CGRP (0.1 µM) or PBS in the presence of peptidase inhibitor (1 µM) for another 96h. Expression of PD-1, Lag-3, and Tim-3, as well as IFN-γ, TNF-α, and IL-2, was immunophenotyped by flow cytometry using a LSRFortessa or FACSCanto II (Becton Dickinson). Cytokines expression levels were analyzed after in vitro stimulation (PMA/ionomycin; see Intracellular cytokine staining).

In vitro cytotoxic CD8+ T-cell stimulation with neuron-conditioned media. Naive or ablated DRG neurons were cultured (72h) in Neurobasal-A medium supplemented with 0.05 ng/µL NGF (Life Technologies, #13257-019) and 0.002 ng/µL GDNF (PeproTech, #450-51-10). After 48h, the neurobasal medium was removed, neurons were washed with PBS, and 200 µL/well of T-cell media supplemented with 1 µL/mL peptidase inhibitor (Sigma, #P1860) and, in certain cases, capsaicin (1µM) or KCl (50mM) was added to DRG neurons. The conditioned media or vehicle were collected after 30min and added to Tc1 CD8+ T-cells for another 96h. The CD8+ T-cells expression of exhaustion markers (PD-1, Lag-3, Tim-3) and cytokine (IFN-γ, TNF-α, IL-2) were analyzed by flow cytometry using a LSRFortessa or FACSCanto II (Becton Dickinson). Cytokines expressions were analyzed after in vitro stimulation (PMA/ionomycin; see Intracellular cytokine staining).

CD8+ T-cell and DRG neurons co-culture. Naive DRG neurons (2×104) were seeded in a 96-well-plate with T-cell media (supplemented with 0.05 ng/µL NGF (Life Technologies, #13257-019), 0.002 ng/µL GDNF (PeproTech, #450-51-10). One day after, Tc1 CD8+ cells (1×105) were added to the neurons in the presence of IL-2 (BioLegend, #575408). In some instances, co-cultures were stimulated with either capsaicin (1 µM) or KCl (50mM). After 96h, the cells were collected by centrifugation (5

min at 1300 rpm), stained, and immunophenotyped by flow cytometry using a LSRFortessa or FACSCanto II (Becton Dickinson). Cytokines expression were analyzed after in vitro stimulation (PMA/ionomycin; see Intracellular cytokine staining).

OT1 CD8+ T-cells induced B16F10 elimination. 2×104 naive TRPV1Cre::QuASR2-eGFPfl/wt DRG neurons were co-cultured with 1×105 B16F10-mCherry-OVA overnight in T-cell media (supplemented with 0.05 ng/µL NGF (Life Technologies, #13257-019), 0.002 ng/µL GDNF (PeproTech, #450-51-10). One day after, 4 ×105 stimulated OVA-specific CD8+ T-cells under Tc1 condition were added to the co-culture. After 48h, the cells were detached by trypsin (Gibco, #2062476) and collected by centrifugation (5 min at 1300 rpm), stained using anti-Annexin V, 7-AAD (BioLegend, #640930), and anti-CD8 for 20 minutes at 4oC, and were immunophenotyped by flow cytometry using a FACSCanto II (Becton Dickinson). Cytokines expression were analyzed after in vitro stimulation (PMA/ionomycin; see Intracellular cytokine staining).

Neuron's conditioned media impact on OT1 CD8+ T-cells induced B16F10 elimination, 4×105 stimulated OVA-specific CD8+ T-cells were added to 1×105 B16F10-mCherry-OVA and treated with fresh condition media (1:2 dilution). After 48h, cells were stained using anti-Annexin V, 7-AAD (BioLegend, #640930), and anti-CD8 for 20 minutes at 4oC, and were immunophenotyped by flow cytometry using a LSRfortessa or FACSCanto II (Becton Dickinson). For CGRP, 4×105 stimulated OVA-specific CD8+ T-cells were added to 1×105 B16F10- mCherry-OVA and treated with CGRP (100nM). After 24h the cells were stained using anti-Annexin V, 7-AAD (BioLegend, #640930), and anti-CD8 for 20 minutes at 4oC, and were immunophenotyped by flow cytometry using a LSRFortessa or FACSCanto II (Becton Dickinson). Cytokines expression were analyzed after in vitro stimulation (PMA/ionomycin; see Intracellular cytokine staining).

Adoptive transfer of RAMP1wt or RAMP1-/- CD8 T-cells. Total CD8+ T-cells were isolated from the spleen of wild-type (CD45.1+) or RAMP1-/- (CD45.2+) mice, expanded and stimulated in vitro using a mouse T-cell Activation/Expansion Kit (Miltenyi cat #130-093-627). CD8+ cells from RAMP1-/- and RAMP1wt were injected separately or 1:1 mix through tail vein of RAG1-/- mice. One week after, the mice were inoculated with B16F10-mCherry-OVA cancer cells (5×105 cells; i.d.). On day 10, tumors were harvested and RAMP1-/- (CD45.2+) and RAMP1wt (CD45.1+) CD8+ T-cells were immunophenotyped using a FACSCanto II (Becton Dickinson) or FACS-purified using a FACSAria IIu cell sorter (Becton Dickinson).

Adoptive T-cell transfer in RTX-exposed mice. CD8+ T-cells were isolated from OT-1 mice spleens and magnet-sorted (StemCell; #19858). Naïve CD8+ T-cells (CD8+CD44lowCD62Lhi) cells were then FACS-purified using a FACSAria IIu cell sorter (Becton Dickinson) and injected (1x106 cells; i.v., tail vein) to vehicle- or RTX-exposed RAG1-/- mice.

RNA sequencing of triple co-culture and data processing. 1×104 naive TRPV1Cre::QuASR2fl/wt DRG neurons were co-cultured with 1×105 B16F10-mCherry-OVA overnight in T-cell media (supplemented with 0.05 ng/µL NGF (Life Technologies, #13257-019), 0.002 ng/µL GDNF (PeproTech, #450-51-10). One day after, 4 ×105 stimulated OVA-specific CD8+ T-cells under Tc1 condition were added to the co-culture. After 48h, the cells were detached and TRPV1 neurons (CD45- eGFP+ mCherry-), B16F10-mCherry-OVA (CD45- eGFP- mCherry+), and OVA-specific CD8+ T-cells (eGFP- mCherry-CD45+ CD3+ CD8+) were FACS-purified using a FACSAria IIu cell sorter (Becton Dickinson) prior to sequencing.

RNA sequencing of cancer and neurons co-culture and data processing. 1×104 naive TRPV1Cre::QuASR2-eGFPfl/wt DRG neurons were co-cultured with 5×104 B16F10-mCherry-OVA overnight in complete Dulbecco's Modified Eagle's Medium high glucose (DMEM, Corning, #10-013-CV) supplemented with 10% fetal bovine serum FBS, Seradigm, #3100), 1% penicillin/streptomycin (Corning, #MT-3001-Cl), 0.05 ng/µL NGF (Life Technologies, #13257-019), 0.002 ng/µL GDNF (PeproTech, #450-51-10). After 48h, the cells were detached and TRPV1 neurons (eGFP+ mCherry-), and B16F10-mCherry-OVA (eGFP-mCherry+) were FACS-purified using a FACSAria IIu cell sorter (Becton Dickinson) prior to sequencing.

Instrument

Cells were immunophenotyped using a LSRFortessa (Becton Dickinson) or FACSCanto II (Becton Dickinson).
Cells were purified using a FACSAria IIu cell sorter (Becton Dickinson)

Software

Data were analyzed using FlowJo v10.0.0 software (Tree Star)

Cell population abundance

For sorting experiments (i.e. CD8, neurons or B16F10 cells), flow cytometry was used and >96% purity was achieved

Gating strategy

Relevant gating strategy are provided as extended data figure 6A, 9F, 10W

To gate samples for FACS analysis, cell were initially gated by FSC-A vs SCS for the exclusion of debris and identification of relevant population (lymphocytes) by size and granularity. For single cells, samples were further gated by FSC-A vs FSC-H. Live cells were finally gated and identified by using fixable dye or fluorescent reporters.

☒ Tick this box to confirm that a figure exemplifying the gating strategy is provided in the Supplementary Information.
